
JOURNAL INFORMATION
==============================
NLM Title Abbreviation: Insights Imaging
Journal ID: Insights Imaging
NLM Journal ID: 101532453

Insights into Imaging

EISSN: 1869-4101
Publisher: Springer

ARTICLE INFORMATION
==============================
PMCID: PMC10692046
DOI: 10.1186/s13244-023-01518-2
Article ID: 1518
Article version: 1
Subjects: Meeting Abstracts

ESHNR 2023 Book of Abstracts

Electronic publication date: 2023 Oct 31
Publication date: 2023 Oct
Volume: 14
Issue: Suppl 3
Issue sponsor: Publication costs were funded by the European Society of Head and Neck Radiology.
Electronic Location ID: 175
Copyright: © The Author(s) 2023
License: Open Access This article is licensed under a Creative Commons Attribution 4.0 International License, which permits use, sharing, adaptation, distribution and reproduction in any medium or format, as long as you give appropriate credit to the original author(s) and the source, provide a link to the Creative Commons licence, and indicate if changes were made. The images or other third party material in this article are included in the article's Creative Commons licence, unless indicated otherwise in a credit line to the material. If material is not included in the article's Creative Commons licence and your intended use is not permitted by statutory regulation or exceeds the permitted use, you will need to obtain permission directly from the copyright holder. To view a copy of this licence, visit http://creativecommons.org/licenses/by/4.0/.
License URL: https://creativecommons.org/licenses/by/4.0/

Conference name: ESHNR 2023: 35th Annual Meeting and Refresher Course
Conference location: St. Julian’s, Malta
Conference date: 5-7 October 2023
issue-copyright-statement: © European Society of Radiology (ESR) 2023

==============================

eshnr
european society of
head and neck radiology

### Insights into Imaging

Education and strategies in European radiology

ESHNR 2023 Book of Abstracts / Volume 14 / Supplement 3 / October 2023

ESHNR 2023 / 35th Annual Meeting and Refresher Course
October 5–7 / St. Julian's, Malta

ESR
European Society of Radiology eshnr
european society of
head and neck radiology

### Masterclass in H&N radiology

December 07-09, 2023
Leiden, The Netherlands

Register now!

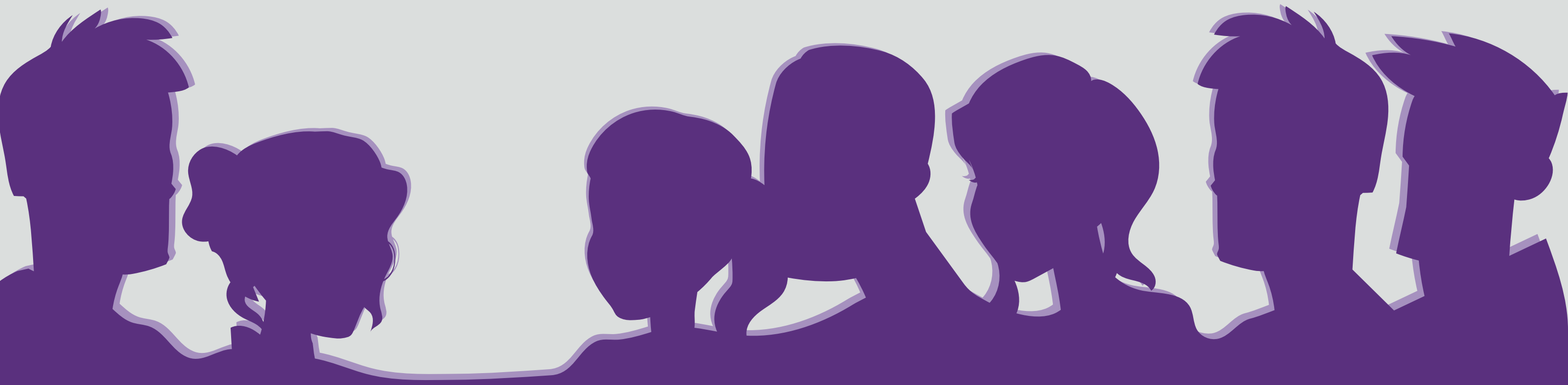

www.eshnr.eu

### Congress Programme

The Scientific Programme will offer a wide spectrum of lectures covering the scope of head and neck radiology, including spatial anatomy, cancer imaging, the orbit, sinonasal and nasopharyngeal region, temporal bone, larynx and hypopharynx, thyroid, parathyroid and salivary glands, led by distinguished speakers from our society and worldwide. As always, there will be sessions dedicated to scientific presentations where you can familiarize yourself with the latest developments in head and neck radiology.

### Organising Secretariat

Education Congress Research GmbH/ESHNR
Am Gestade 1
1010 Vienna, Austria
office@eshnr.eu | www.eshnr.eu

### Confirmation of Attendance

Each participant who successfully registered and attended the live event will receive a confirmation of attendance after the meeting.

### Disclaimer

The ESHNR 2023 Book of Abstracts summarises the presentations accepted to be held at the Annual Scientific Meeting – St. Julian's/Malta, October 2023. Abstracts were submitted by the authors warranting that good scientific practice, copyrights and data privacy regulations have been observed and relevant conflicts of interest declared. Abstracts reflect the authors' opinions and knowledge. The ESHNR does not give any warranty about the accuracy or completeness of medical procedures, diagnostic procedures or treatments contained in the material included in this publication. The views and opinions presented in all given abstracts and presentations, including scientific, educational and professional matters, do not necessarily reflect the views and opinions of the ESHNR. In no event will the ESHNR be liable for any direct or indirect, special, incidental, consequential, punitive or exemplary damages arising from the use of these abstracts.

In preparing this publication, every effort has been made to provide the most current, accurate, and clearly expressed information possible. Nevertheless, inadvertent errors in information can occur. The ESHNR is not responsible for typographical errors, accuracy, completeness or timeliness of the information contained in this publication.

Insights Imaging (2023) 14 (Suppl 3)
https://doi.org/10.1186/s13244-023-01518-2
Published: 04 October 2023.

eshnr

### ESHNR Membership

**The European Society of Head and Neck Radiology, Europe's leading head and heck imaging society, welcomes anyone with a substantial interest in head and neck imaging.**

### Membership benefits

» **Reduced registration fee for**
- the ESHNR Annual Meeting and Refresher Course
- the ESHNR Masterclass
- all additional ESHNR events

» **Eligibility to apply for**
- the ESHNR Mentoring Programme
- the European Board in Head and Neck Radiology Diploma
- the ESOR-ESHNR fellowship programme

» **Free access to**
- live ESHNR webinars (incl. CME certificate)
- all ESHNR webinars recordings
- live ESHNR Case webinars ***NEW***
- all ESHNR Case webinars recordings ***NEW***
- live ASHNR-ESHNR joint webinars (incl. CME certificate)
- all ASHNR-ESHNR joint webinars recordings

**plus** » Membership certificate » ESHNR newsletter » and much more!

**Apply now on www.eshnr.eu!**

eshnr

### ESHNR Mentoring Programme

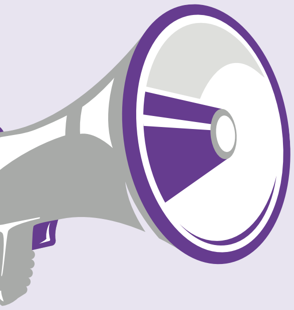

new support initiative

*

one year mentoring programme

**ESHNR calls for mentors and mentees and will match and introduce them**

**Programme coordinators**
Steve Connor, London/UK
Soraya Robinson, Vienna/AT

**Become a mentee or mentor**

www.eshnr.eu

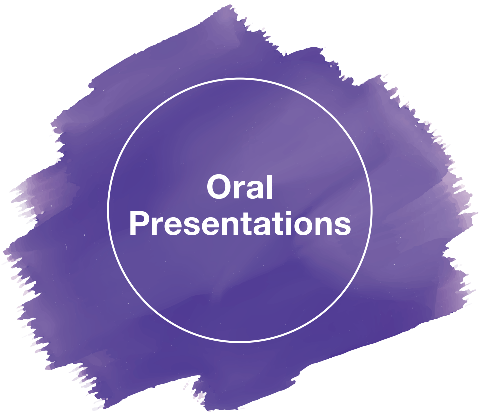

### A "head to head" comparison of delayed post gadolinium MRI descriptors for the diagnosis of Meniere's Disease and the added diagnostic value of vestibular aqueduct signal discontinuity

*Authors: S. Connor[1], P. Touska[2], I. Pai[2]; [1]King's College Hospital, Neuroradiology, London, United Kingdom, [2]Guy's and St Thomas' Hospital, London, United Kingdom*

**Purpose/Objectives:** The MRI diagnosis of MD is based on the evaluation of descriptors on delayed post-gadolinium imaging. Since only a limited number of MRI features have been applied in previous individual studies, this introduces bias when comparing their overall diagnostic performance. The purpose of this study was to evaluate the diagnostic performance and reliability of MRI descriptors used for the detection of Menière's Disease (MD) ears on delayed post-gadolinium MRI. In addition, it was aimed to determine which combination of descriptors should be optimally applied and whether analysis of the vestibular aqueduct contributes to the diagnosis.
**Methods and materials:** This retrospective single centre case-control study evaluated consecutive delayed post-gadolinium MRI of patients with Menièriform symptoms between Dec 2017-March 2023. A comprehensive range of MRI descriptors of endolymphatic hydrops were derived from 4 established grading systems whilst novel descriptors (visibility, hyperintensity and continuity of vestibular aqueduct signal) were also defined. Perilymphatic enhancement (PLE) was analysed qualitatively and quantitatively. Two observers evaluated 17 MRI descriptors of MD and quantified perilymphatic enhancement (PLE) in the cochlea. Definite MD ears according to the 2015 Barany Society criteria were compared to control ears. Cohen's kappa and diagnostic odds ratio (DORs) were calculated for each descriptor. Forward step wise logistic regression determined which combination of MRI descriptors would best predict MD ears, and the area under the ROC curve for this model was measured.
**Result:** 227 patients (mean age 48.3 ± 14.6, 99 men) with 96 MD and 78 control ears were evaluated. The presence of any saccular abnormality (absent, as large as or confluent with the utricle) performed best with a DOR of 292.6 (95% CI, 38.305-2235.058). All vestibular aqueduct descriptors demonstrated excellent reliability and with DORs of 7.761 (95% CI, 3.517-17.125) to 18.1(95% CI, 8.445-39.170). Combining these saccular abnormalities with asymmetric cochlear PLE and an incompletely visualised vestibular aqueduct, correctly classified 90.2% of cases (sensitivity 84.4%, specificity 97.4%, AUC 0.938).
**Conclusion:** The combination of either absent, enlarged or confluent saccules are the best predictors of MD, however incomplete visualisation of the vestibular aqueduct adds value to the diagnosis.

**Keywords:** Meniere's Disaese; MRI; endolymphatic hydrops; gadolinium

References:
[1] Lopez-Escamez JA, Carey J, Chung WH et al, (2015), Diagnostic criteria for Menière's disease, Journal of Vestibular Research, 25(1), 1-7
[2] Committee on Hearing and Equilibrium, (1972), Report of Subcommittee on Equilibrium and its measurement. Meniere's disease: criteria for diagnosis and evaluation of therapy for reporting., Trans Am Acad Ophthalmol Otolaryngol, 76(6), 1462-4
[3] Pearson BW, Brackmann DE, (1985), Committee on Hearing and Equilibrium Guidelines for Reporting Treatment Results in Meniere's Disease., Otolaryngology–Head and Neck Surgery, 6;93(5), 579-81
[4] Committee on Hearing and Equilibrium Guidelines for the Diagnosis and Evaluation of Therapy in Meniere's Disease, (1995), Otolaryngology–Head and Neck Surgery, 113(3), 181-5

### Accuracy of dynamic contrast-enhanced (DCE) MRI generated Time- signal intensity curves in diagnosis and differentiation of salivary neoplasms

*Author: M. S. Swarup; Vardhman Mahavir Medical College and Safdarjung Hospital, Radiology, New Delhi, India*

**Purpose/Objectives:** To evaluate accuracy of time-signal intensity curves (TIC) generated from dynamic contrast enhanced (DCE) MRI and to assess it's role in diagnosis and differentiation of various salivary neoplasms
**Methods and materials:** A total of 28 patients with neoplastic mass lesions of various salivary glands were included. All the patients underwent multiparametric MRI of the involved region on a 3T MRI scanner according to the standard departmental protocol including dynamic contrast enhanced MR imaging. DCE MRI data set were analysed on the work station by vendor provided software to obtain time-signal intensity curves (TIC). MRI findings were correlated with histopathological examinations. TICs were classified into three types based on peak enhancement time (PET) and washout ratio (WR). Type I pattern defines persistent and progressive enhancement (PET > 150 seconds with no washout). Type II pattern includes early enhancement with prominent washout (PET < 150 seconds, WRs > 30%). Type III pattern had PET equal to or shorter than 150 seconds, WRs < 30% (rapid enhancement with low washout).

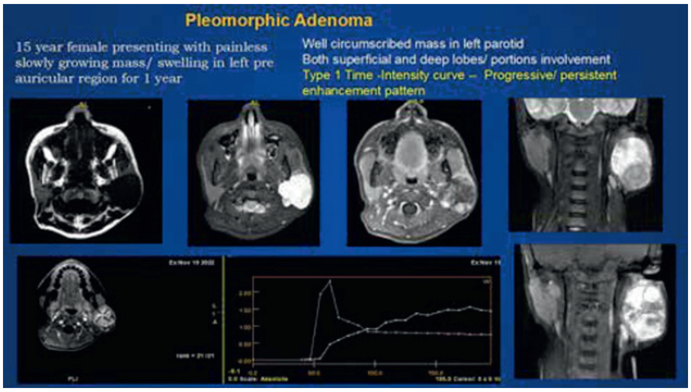

Pleomorphic Adenoma

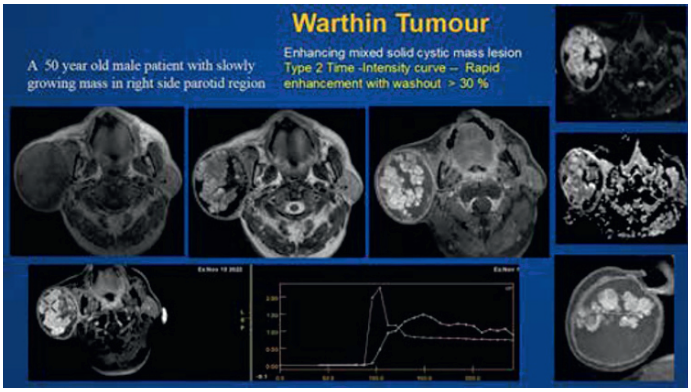

Warthin tumor

**Result:** Out of the 28 cases, 22 were parotid neoplasms, 4 were submandibular gland origin and 2 were of minor salivary gland origin. Most of the pleomorphic adenomas (PA) and Warthin tumors (WT) reveal type I and type II enhancement patten (TICSs) respectively. Type III enhancement pattern was more commonly seen in malignant salivary neoplasms. Two cases of lymphomatous involvement demonstrated type II TIC. Statistical analysis revealed more than 85 % accuracy for differentiation of benign (PA and WT) and malignant salivary neoplasms, which is further increased with consideration of conventional and diffusion MR imaging features.
**Conclusion:** TICs obtained from DCE MRI improve the diagnostic accuracy and are helpful in differentiation of various salivary neoplasms complimentary to conventional and diffusion MRI. It can accurately predict the behaviour of those salivary gland neoplasms, which are indeterminate or borderline on conventional imaging.

**Keywords:** Salivary gland, tumors, parotid, pleomorphic adenoma

### Benign lymphoepithelial cysts (BLECs) in HIV-infected patients

*Authors: F. Pandolfo[1], F. Bencivinni[2], A. Cascio[3], V. Galbo[3], G. La Tona[1], A. Lo Casto[1]; [1]Sezione di Scienze radiologiche, BIND, Università degli studio di Palermo, AOUP P Giaccone, Palermo, Italy, [2]UOS Radiologia Odontoiatrica e Maxillo-facciale, AUOP P. Giaccone, Palermo, Italy, [3]UOC Malattie infettive, Promise, AOUP P. Giaccone, Palermo, Italy*

**Purpose/Objectives:** HIV-associated head and neck lesions develop in more than 50% of HIV-positive patients, and occur in almost 80% of all AIDS patients.
In 1-10% of HIV+ patients there is swelling of the salivary glands, often related to inflammation, infection, tumours and BLECs. These last are lesions of the parotid gland, mono- or bi-lateral, single or multiple, of variable size, with liquid content and vascularised wall, associated with cervical lymphadenopathy. Their aetiology is based on two hypotheses, origin from entrapment of the parotid glandular epithelium in HIV-colonised intraparotid lymph nodes, or from migration of infected cells into the parotid gland, with lymphatic proliferation and obstructing ductal dysplasia, with cyst formation, reservoir of p24 viral antigen and with RNA concentrations a thousand times higher than in plasma. Clinically there is a gradual, non-painful, bilateral swelling of the parotids with diffuse cervical lymphadenopathy. A case history of HIV+ patients in whom BLECs were detected is described.
**Methods and materials:** 70 (16 women and 54 men) HIV+ patients from the Department of Infectious disease of our hospital, who underwent CT or MR exams between 2015 and 2021 were retrospectively analysed.
**Result:** BLECs were found in 6 HIV+ patients, men, age range 30-57 years. 3/6 patients presented bilateral involvement, 1/6 parotid calcifications, in 4/6 there were associated mediastinal and/or abdominal lymphadenopathies, 3/6 pulmonary or cerebral infections by opportunistic pathogens, 1/6 Kaposi's sarcoma. In 1/6 patients there was complete resolution of the lesions at follow up after antiretroviral therapy.

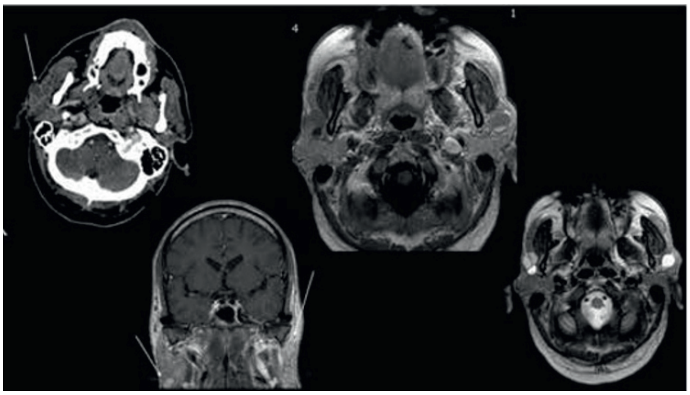

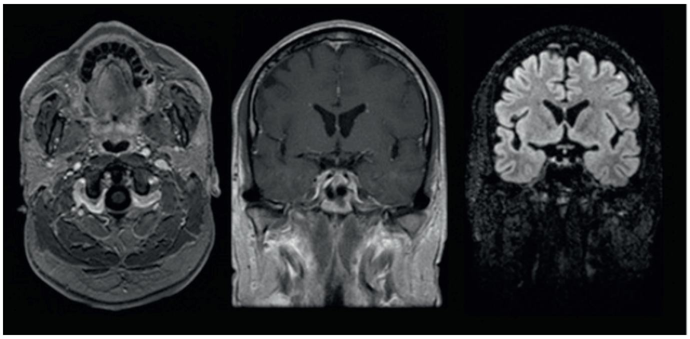

**Conclusion:** BLECs, despite being benign lesions, are of considerable importance both because they constitute a reservoir of the virus within the body and because, by causing parotid swelling, they can represent the onset symptom of HIV, entering into differential diagnosis with other parotid diseases.

### Can Intraoral Ultrasound Predict Oral Squamous Cell Carcinoma Histological Risk Factors? A preliminary experience

*Authors: S. Caprioli[1,2], G. Giordano[3], C. Conforti[1], F. Marchi[3,4], A. Iandelli[4], F. Borda[5], M. Fiannacca[6], G. Cittadini[1]; [1]IRCCS Ospedale Policlinico San Martino, Radiology, Genova, Italy, [2]University of Genova, DIMI, Genova, Italy, [3]University of Genova, DISC, Genova, Italy, [4]IRCCS ospedale policlinico San Martino, Otorhinolaryngology, Genova, Italy, [5]University of Genova, DISSAL, Genova, Italy, 6Ente Ospedaliero "Ospedali Galliera", Neuroradiology, Genova, Italy*

**Purpose/Objectives:** Over the past two decades, substantial progresses have been made in multidisciplinary care for oral cavity squamous cell carcinoma (OSCC). A significant improvement was the introduction of depth of invasion (DOI) into the 8th edition of TNM staging system for OSCC[1]. However, an increasing number of studies is showing that DOI is not completely adequate for risk stratification as local and nodal recurrences seem to have a stronger correlation to histopathological features, such as perineural invasion, lymph-vascular invasion and worse pattern of invasion. Brandwein-Gensler (BG) et al. introduced a histological risk model for predicting outcome in OSCC[2][3]. Recently, considerable progresses have been made in ultrasonography technology: high frequently probes have increasingly been used for better depicting superficial structures, as oral mucosa. Several studies used intraoral ultrasound (IOUS) for predicting DOI in OSCC, but no studies investigated if IOUS may be able to predict histological features of tumor-host interface[4]. This study aims to investigate whether there is a correlation between the ultrasonographic appearance of front of infiltration (FOI) and histological risk factors.
**Methods and materials:** Patients affected by biopsy-proven OSCC who underwent IOUS, surgery and histopathological assessment of DOI and risk factors were enrolled. IOUS was performed with a 22-8 MHz hockey-stick probe. During IOUS, ultrasonographic DOI (usDOI) and FOI appearance were assessed.

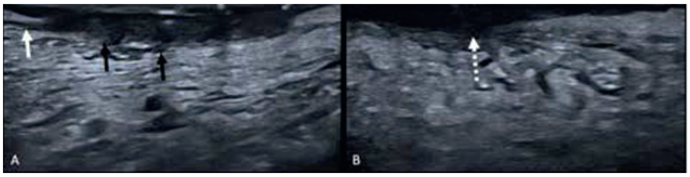

A: pT2 squamous cell carcinoma of the lateral border of the tongue with spiculated margins (black arrow), infiltrating superior longitudinal muscle (white arrow); Brandwein-Gensler (BG) score was 3. B: pT1 squamous cell carcinoma of the lateral border of the tongue with smooth margins (white dotted arrow). BG score was 0.

On histology, BG scoring system was used to evaluate risk factor, with a score of 0 indicating low-risk lesions, 1-2 indicating intermediate risk, and ≥3 indicating high risk. Fisher's exact test was used to analyze the relationship between margins appearance and individual risk factors, as well as margins appearance on US and BG score ≥3. Specificity, sensitivity, positive and negative predictive values were calculated for margins appearance on IOUS to predict BG risk predictive score ≥3. Receiving operator characteristics (ROC) curve was calculated. Agreement between DOI and usDOI was assessed using Bland Altman plot.
**Result:** A significant relationship between BG score and margins appearance on ultrasound was found (Fisher exact test, P<0,0001). Sensitivity, specificity, positive predictive value and negative predictive value were 84,6%, 93,3%, 91,7% and 87,5%. Area under ROC curve was 0,896. A high agreement between the usDOI and pDOI, even though a mean systematic error was found between the usDOI and pDOI (0,5 mm).
**Conclusion:** Our preliminary experience showed that IOUS may be able to predict BG score, making it a promising tool not only for OSCC staging, but also for prognostic evaluations. However, further studies have to confirm our results in the future.

**Keywords:** Oral squamous cell carcinoma; ultrasound; prognosis; Brandwein-Gensler score

References:
[1] Amin M.B., Edge S., Greene F., Byrd D.R., Brookland R.K., Washington M.K., Gershenwald J.E., Compton C.C., Hess K.R., Sullivan D.C. , (2017), AJCC Cancer Staging Manual. 8th ed. , Springer; New York, NY, USA
[2] Brandwein-Gensler M, Teixeira MS, Lewis CM, Lee B, Rolnitzky L, Hille JJ, (2005), Oral squamous cell carcinoma: histologic risk assessment, but not margin status, is strongly predictive of local disease-free and overall survival, Am J Surg Pathol, 167–78, 29
[3] Li Y, Bai S, Carroll W, Dayan D, Dort J, Heller K, et al. , (2013), Validation of the risk model: high risk classification and tumour pattern of invasion predict outcome for patients with low stage oral cavity squamous cell carcinoma, Head Neck Pathol, 211–23, 7
[4] Caprioli S, Casaleggio A, Tagliafico AS, Conforti C, Borda F, Fiannacca M, Filauro M, Iandelli A, Marchi F, Parrinello G, Peretti G, Cittadini G, (2022), High-Frequency Intraoral Ultrasound for Preoperative Assessment of Depth of Invasion for Early Tongue Squamous Cell Carcinoma: Radiological-Pathological Correlations, Int J Environ Res Public Health, 14900, 19(22)

### Cartilage invasion in squamous cell carcinoma of the larynx: CT and MR assessment

*Authors: T. Perillo[1], L. Ugga[2], M. Fedele[2], B. Valente[2], G. Valente[2], G. Rossi[3], R. Cuocolo[4], R. Palladino[3], A. Manto[1]; [1]"Umberto I" Hospital, Department of Neuroradiology, Nocera Inferiore, Italy, [2]University of Naples Federico II, Department of Advanced Biomedical Sciences, Naples, Italy, [3]"Umberto I" Hospital, Otolaryngology, Nocera Inferiore, Italy, [4]University of Salerno, Department of Medicine, Surgery and Dentistry, Salerno, Italy*

**Purpose/Objectives:** Evaluation of cartilage invasion in squamous cell carcinoma of the larynx is crucial for surgical strategy, aimed to laryngeal function preservation when possible[1]. Both CT and MR can be used to assess cartilage infiltration, although the evaluation is not always easy, especially for differentiation of reactive changes from tumor[2][3].
**Methods and materials:** In this retrospective analysis, consecutive patients with squamous cell carcinoma of the larynx who underwent surgery within 7 days from imaging at our Institution have been included. Histologic report was available for all patients. Imaging examinations have been assessed by two readers: a radiology resident in its second year of residency (reader 1) and a radiologist with 5 years experience in head and neck imaging (reader 2). Both of them were blind to the histologic report. With regard to MR scans, a first evaluation of conventional sequences was followed by a second assessment with DWI inclusion after 2 weeks.
**Result:** We included 83 patients (M=69; F=14 ), of whom 66 underwent CT and 17 MR for laryngeal cancer. Regarding MR assessment, reader 1 accuracy in identifying cartilage invasion without DWI was 52.9% (9/17), whereas with DWI it raised to 64.7% (11/17). For reader 2, accuracy was equal to 76.5% (13/17) without DWI and 94.1% (16/17) when this sequence was included. With regard to CT examination, the accuracy values of readers 1 and 2 were 62% (41/66) and 80.3% (53/66), respectively.
**Conclusion:** CT and MR are both useful for cartilage invasion identification in patients with squamous cell tumors of the larynx. The inclusion of DWI in the MR protocol can increase the accuracy reducing the false positive rate, which is mainly due to peritumoral inflammatory changes possibly misinterpreted as tumor invasion, independently from the radiologist experience.

**Keywords:** Laryngeal Cancer; MR, CT, DWI

References:
[1] Li B, Bobinski M, Gandour-Edwards R, Farwell DG, Chen AM, (2011), Overstaging of cartilage invasion by multidetector CT scan for laryngeal cancer and its potential effect on the use of organ preservation with chemoradiation, British Journal of Radiology, https://doi.org/10.1259/bjr/66700901
[2] Gilbert K, Dalley RW, Maronian N, Anzai Y. , (2010), Staging of laryngeal cancer using 64-channel multidetector row CT: comparison of standard neck CT with dedicated breath-maneuver laryngeal CT, AJNR, https://doi.org/10.3174/ajnr.A1796
[3] Becker M, Zbaren P, Casselman JW, Kohler R, Dulguerov P, Becker CD. , (2008), Neoplastic invasion of laryngeal cartilage: reassessment of criteria for diagnosis at MR imaging. Radiology. , Radiology, https://doi.org/10.1148/radiol.2492072183

### Correlation of Gene Expression with Magnetic Resonance Imaging Features of Retinoblastoma: A Multi-Center Radiogenomics Validation Study

*Authors: R. Jansen, C. de Bloeme, M. de Jong, P. de Graaf; Amsterdam UMC, Radiology, Amsterdam, The Netherlands*

**Purpose/Objectives:** To validate associations between MRI features and gene expression profiles in retinoblastoma, thereby evaluating the repeatability of radiogenomics in retinoblastoma.
**Methods and materials:** In this retrospective multicenter cohort study, retinoblastoma patients with gene expression data and MRI were included. MRI features and matched genome-wide gene expression data were used to perform radiogenomic analysis. Expression data from each center were first separately processed and analyzed. The end product normalized expression values from different sites were subsequently merged by their Z-score to permit cross-sites validation analysis. In the radiogenomic analysis, the MRI features were correlated with expression of photoreceptorness, a gene-expression signature informing on disease progression. Outcomes were compared to outcomes in a previous described cohort.
**Result:** Thirty-six retinoblastoma patients were included, 15 were female (42%) and mean age was 24 (SD 18) months. Similar to the prior evaluation, this validation study showed that low photoreceptorness gene expression was associated with advanced stage imaging features. Validated imaging features associated with low photoreceptorness were multifocality, a tumor encompassing the entire retina or entire globe, and a diffuse growth pattern (all $P < 0.05$). There was a considerable amount of radiogenomic associations that were not validated.

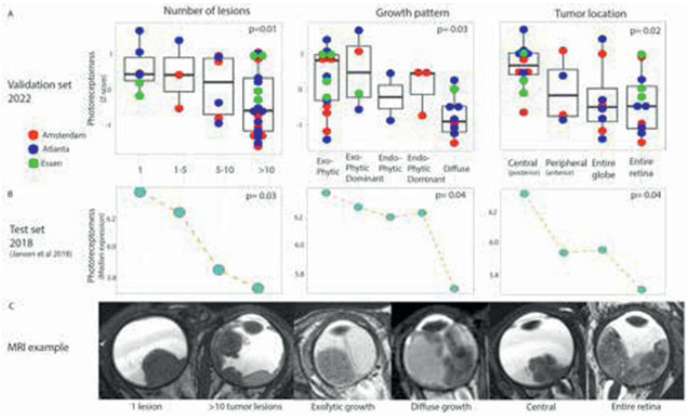

Boxplots showing correlations between MR imaging features and gene expression of the photoreceptorness profile with similarities between the test set (panel A) and the validations set (panel C). Imaging feature examples are shown in panel C.

**Conclusion:** A substantial part of associations could not be validated, underlining the importance of validation studies in radiogenomics. Nevertheless, cross-center validation of imaging features associated with photoreceptorness gene expression highlighted the capability radiogenomics to non-invasively inform on genomic subtypes in retinoblastoma.

**Keywords:** Retinoblastoma

### Correlation of sonographic and histopathological findings in indeterminate thyroid nodules: A multicentre study

*Authors: S. Billingsley[1], S. Viner[2], C. Vincent[3], M. Karim4; [1]Leeds teaching hospitals nhs trust, Radiology, Leeds, United Kingdom, [2]Bradford Teaching Hospitals NHS Trust, Bradford, United Kingdom, [3]Leeds Teaching Hospitals NHS Trust, York, United Kingdom, 4Leeds Teaching Hospitals NHS Trust, Bradford, United Kingdom*

**Purpose/Objectives:** The investigation of thyroid nodules forms a large portion of the head and neck radiologists' workload. In the UK, thyroid nodules are sonographically classified according to the British Thyroid Association (BTA) guidelines published in 2014 (1), with nodules classified 1 to 5, 1 being normal, 5 being malignant. Many patients with U3 and U4 nodules, classified as either indeterminate or suspicious, go on to have guided biopsies or fine needle aspiration (FNA). Tissue is subsequently analysed & allocated to a histopathological category according to the THY criteria (2). This study aims to correlate the radiological findings with histopathological findings, and how these findings translate to patient management.
**Methods and materials:** Three NHS trusts (Bradford, York and Mid Yorkshire Teaching Hospitals) were involved in data collection. Local imaging software Enterprise Imaging (EI) was used to search patients who had attended an 'ultrasound guided biopsy, thyroid' between the dates of 31/3/2023 and 1/10/2022. Ultrasound imaging and reports were reviewed, as were histopathological reports, multidisciplinary team documentation and clinic letters. Data collected include relevant imaging findings, size of nodule (mm), BTA classification, histological THY classification and any available follow up histology. Data analysis was completed using Microsoft excel. No patient identifiable data is published.
**Result:** This data demonstrates that the majority of patients with U4 sonographic findings have histopathological findings consistent with either malignancy or malignancy cannot be excluded/concern for malignancy (76%). Remaining U4 lesions were Thy1 with insufficient cells for analysis. None had overtly benign histopathology.
100% of U3 and U4 classified nodules with Thy3 histopathology underwent diagnostic hemithyroidectomy. 100% of patients with Thy4 and Thy5 histopathological gradings underwent thyroid intervention or were scheduled for such at the time of data collection.
One third of FNA samples were Thy1, hence non-diagnostic; of these samples, 64% came from nodules under 10mm in maximum diameter. This is in comparison to 9% of diagnostic Thy2,3,4 and 5 samples.

**Conclusion:** This study demonstrates strong clinicopathological correlation in the features of thyroid nodules and reinforces the functionality of both the BTA and Thy classification systems in clinical practice. The combination of radiological and histopathological categorisation directly impacts decisions regarding surgical follow up. It also raises the question of whether thought should be given to subjecting a patient to FNA when the lesion is under 10mm in maximum diameter given the low diagnostic yield.

References:
[1] Chng , (2018), Diagnostic performance of ATA, BTA and TIRADS sonographic patterns in the prediction of malignancy in histologically proven thyroid nodules, Singapore J Med
[2] Almquist et al, (2019), Surgical management of cytologically indeterminate thyroid nodules, Gland Surg

### Cranial nerves imaging using an optimized 3D-Neurography sequence performed on a 1.5T scanner

*Authors: P. Rondi[1], N. Di Meo[1], M. Ravanelli[1], R. Maroldi[2], D. Farina[1]; [1]Università degli studi di Brescia, Spedali Civili, Radiologia 2, Brescia, Italy, [2]Università degli studi di Brescia, Brescia, Italy*

**Purpose/Objectives:** The aim of this study is to evaluate image quality and identification of cranial nerves (CNs) on an optimized 3D neurography sequence performed on a 1.5 T scanner.
**Methods and materials:** 25 patients were included in this study. In each patient, a 3D-space STIR black blood sequence was performed after Gadolinium administration; the acquisition time of the sequence was 5'11''. MIP thin (4mm) reconstructions were performed, to improve the depiction of the extracranial course of CNs (Figure 1)

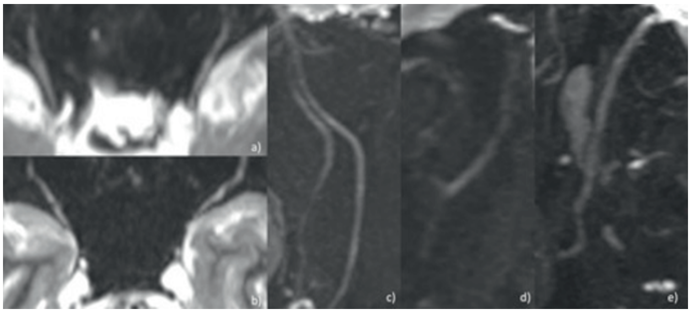

Figure 1: MIPthin reconstruction of CNs, in order: V1 (a); maxillary and proximal portion of infraorbital nerve, V2 (b); mandibular nerve (V3), lingual and inferior alveolar branch(c); common trunk, superior and inferior branch of VII CN (d); hypoglossal nerve(e)

Two operators with different experience in head and neck imaging evaluated the studies assigning a quality score from 1 to 4 (1 insufficient image quality; 4 excellent image quality) and a detection score from 0 to 3 (0: not visible; 3: visible and well delineated) for selected extracranial segments of V, VII, IX-XII CN.
In detail, the evaluated nerves were: V1 (intraorbital), V2 (maxillary and infraorbital n.), V3 (mandibular nerve, alveolar inferior nerve, lingual nerve extra and intraoral tract), VII (intramastoid and intraparotid, common trunk, superior branch, inferior branch), mixed cranial nerves (IX-XII), XI and XII (both distal to the point of separation from mixed cranial nerves).
**Result:** The average overall quality of neurographic sequence was 3.24. The segment-by-segment nerve detection obtained variable score, spanning from 0.94 (inferior branch of facial nerve) to 2.88 (V3).
Detection score were: excellent (above 2) for V1, V2, V3 (mandibular nerve, alveolar inferior nerve, lingual nerve extraoral tract), VII (intramastoid), IX, X, XI and XII; good (between 1 and 2) for intraoral tract of lingual nerve and for intraparotid segments of facial nerve (common trunk and superior branch);poor (inferior to 1) only for the inferior branch of facial nerve.
Inter-rater agreement ranged from 0.46 (XII) to 0.91 (XI).
Optimal inter-rater agreement (above 0.6) was found for: V CN, VII CN, XII and XI CNs.
**Conclusion:** 3D-neurography sequence acquired after contrast administration allows good to optimal identification of extracranial course of CN.
The selected segments are among the most commonly involved by neurogenic tumors and perineural spread. The possibility to depict such nerves amplifies the role of MRI in presurgical planning.
The robust agreement between the two operators encourages a broader use of this sequence in the clinical setting.

**Keywords:** Cranial Nerve Imaging; 3D Neurography; 1.5 T scanner; MRI; Head and Neck Cancer

### Diagnostic accuracy of MRI in detecting perineural spread of head and neck tumors: a systematic review

*Authors:* *U. Abdullaeva[1], J. Hirvonen[2]; [1]Tashkent City Branch of the Republican Specialized Scientific and Practical Medical Center of Oncology and Radiology, Radiology, Tashkent, Uzbekistan, [2]Tampere University Hospital and Tampere University, Faculty of Medicine and Health Technology, Radiology, Tampere, Finland*

**Purpose/Objectives:** Perineural spread (PNS) of head and neck tumors indicates worse prognosis and higher risk of local recurrence, aggressiveness, and metastatic disease. MRI is considered the overall best method for assessing PNS due to the high soft tissue contrast, multiplanar capability, and accuracy in determining the presence and extent of nerve involvement. However, the diagnostic accuracy of MRI in detecting PNS varies between studies, and PNS needs to be differentiated from reactive or inflammatory nerve swelling. Histopathological proof is required as a reference standard to avoid selection bias and circumferential reasoning. Therefore, we performed a systematic review of the literature regarding the diagnostic accuracy of MRI in detecting PNS when using histopathological evidence from the afflicted nerve as the reference standard.

**Methods and materials:** Previous studies were searched from PubMed and Embase databases. Inclusion criteria were: 1) study published in English from 1/1/1992 to 31/10/2022; 2) patients with head and neck tumor; 3) MRI (with and without contrast enhancement) suggesting PNS; 4) histological or surgical confirmation of PNS; 5) reported number of patients required for assessing diagnostic accuracy: total number of patients undergoing biopsy or surgery after MRI suggestive of PNS. The outcome measures were sensitivity, specificity, and positive predictive value (PPV, the proportion of true positives among all positives).

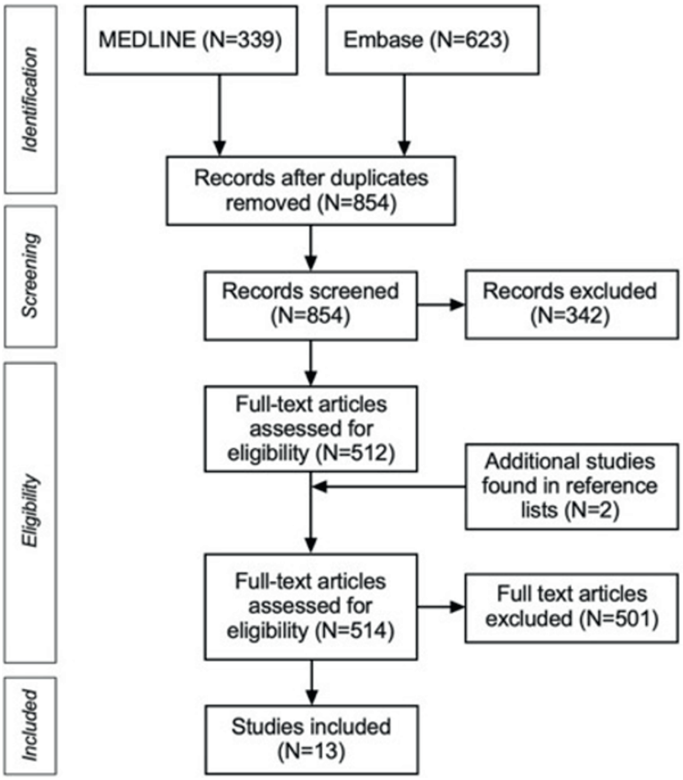

PRISMA flowchart of systematic review

**Result:** We found 13 retrospective studies reporting on 397 nerves samples from 305 patients (mean age 58 years). Sensitivity ranged from 0.46 to 1.00, and specificity from 0.5 to 1.00, with median values of 0.96 and 0.87, respectively. PPV ranged from 0.31 to 1.00 and had a median of 1.0 and a weighted average of 0.88. A total of 39% of studies used 1.5T, 15% used 3T, and 46% did not report the field strength of the MRI device. The most common PNS features on MRI were nerve thickening and abnormal, asymmetric enhancement. The most common tumor localization included skin, nasophar¬ynx, sinonasal region, and salivary glands. The most commonly encountered tumor histologies were adenoid cystic carcinoma and squamous cell carcinoma. Some papers included benign tumors such as schwannoma, neurofibroma, and meningioma. The most commonly studied cranial nerves were the facial and trigeminal nerves and their branches.

| Study | Year | N patients | N nerves | Sensitivity | Specificity | PPV |
|---|---|---|---|---|---|---|
| Hanna et al. | 2007 | 26 | 38 | 1.00 | 0.85 | 0.88 |
| Nader et al. (DFN model 1) | 2019 | 52 | 52 | 0.73 | 0.88 | 0.62 |
| Nader et al. (DFN model 2) | 2019 | 52 | 52 | 0.46 | 1 | 1.00 |
| Nader et al. (SMF) | 2019 | 49 | 52 | 0.8 | 0.83 | 0.76 |
| Baulch et al. | 2015 | 33 | 57 | 0.95 | 0.84 | 0.92 |
| Gandhi et al. | 2011 | 25 | 48 | 1.00 | 0.94 | 0.97 |
| Warren et al. | 2016 | 48 | 48 | 0.96 | - | 1.00 |
| Chang et al. | 2004 | 8 | 8 | 1.00 | - | 1.00 |
| Nemzek et al. | 1998 | 19 | 45 | 0.95 | - | 1.00 |
| Eisen et al. | 1996 | 22 | 22 | 1.00 | 0.5 | 0.31 |
| Lee et al. | 2008 | 38 | 45 | 0.79 | - | 1.00 |
| Schmalfuss et al. | 2002 | 7 | 7 | 1.00 | - | 1.00 |
| Majoie et al. | 1997 | 2 | 2 | 1.00 | - | 1.00 |
| Shimamoto et al. | 2012 | 13 | 13 | 0.73 | 1.00 | 1.00 |
| Tomura et al. | 1999 | 12 | 12 | 1.00 | - | 1.00 |

Studies included in the systematic review. DFN, descending facial nerve; SMF, stylomastoid foramen; PPV, positive predictive value

**Conclusion:** MRI accurately detects PNS of head and neck tumors and should be considered in patients at risk. Yet, this evidence is based on a limited number of retrospective studies that are likely affected by sampling bias. Other limitations include varying MRI vendors and protocols, varying imaging criteria, and omission of studies using a clinical rather than histological reference standard.

**Keywords:** perineural spread, head and neck cancer, MRI

### Ethanol Ablation of Ranulas: Long-Term Follow-Up Results and Risk Factor Analysis for Recurrence and Need for Surgery After Treatment

*Authors: P. S. Suh[1], J. H. Lee[1], Y. H. Roh[1], H. H. Moon[1], S. R. Chung[1], Y. J. Choi[1], J. H. Baek[1], S.-H. Choi[2]; [1]Asan Medical Center, Radiology, Seoul, Republic of Korea (South Korea), [2]Asan Medical Center, Otolaryngology, Seoul, Republic of Korea (South Korea)*

**Purpose/Objectives:** Percutaneous ethanol ablation (EA) is a safe and effective treatment for ranula, but there have been few studies about the long-term follow-up and recurrence of ranula after EA. Our study aimed to evaluate the long-term outcome and risk factors for recurrence and need for surgery in the ranula after EA.
**Methods and materials:** This retrospective study evaluated 57 patients with ranulas treated by EA between July 2009 and March 2021, followed for at least 24 months. Overall recurrence was defined as the residual ranula with clinical symptoms after one or more EA. First local recurrence was assessed with clinical symptoms after the first EA. Analyzed clinical and radiologic factors included patient age, sex, location, type, echogenicity of ranula, number of EA procedures, number of ethanol injections during one session, duration of symptoms before EA, long diameter and volume of ranula, the difference between measured and aspirated volumes, parapharyngeal extension, and sublingual gland herniation. Univariate and multivariate logistic regression analyses were performed to identify risk factors for overall recurrence and need for surgery. Cox proportional hazards models, Kaplan-Meier curves, and log-rank tests were used to evaluate the 2-year first local recurrence-free survival after EA.
**Result:** This study evaluated 33 males and 24 females with a mean age of 26.4 years (range, 3–63 years). Median follow-up after EA was 57 months (range, 24–167 months). Overall recurrence rate was 33.3% (n=19) and 11 patients underwent surgery. Overall recurrence was more common in patients with repetitive EA (OR 2.57, P=0.04 in multivariate analysis) and duration of symptom ≥ 12 months (OR 3.58, P=0.03 in univariate; OR 2.80, P=0.13 in multivariate analysis). Two-year local recurrence-free survival after EA was lower in patients with sublingual gland herniation than in those without herniation (P=0.02). In multivariate analysis, repetitive EA (OR 5.61, P=0.02) and parapharyngeal extension (OR 12.74, P=0.02) were associated with recurrence requiring surgery.
**Conclusion:** Repetitive EA, duration of symptom ≥ 12 months, and sublingual gland herniation are high risk factors for overall recurrence of ranula after EA. Repetitive EA and parapharyngeal extension were significantly associated with need for surgery.

**Keywords:** Ranula, Ethanol ablation

### Feasibility of PET Radiomics Features for Prediction of Bone Invasion in Patients with Oral Squamous Cell Carcinoma

*Authors: M.-Y. Lu[1,2], P.-Y. Chen[1,2], C.-Y. Peng[1,2], X.-P. Lu[3], Y.-H. Liao[4], P.-F. Kao[3,5], W.-C. Shen[4,6]; [1]Chung Shan Medical University Hospital, Division of Oral and Maxillofacial Surgery, Department of Dentistry, Taichung, Taiwan, [2]Chung Shan Medical University, School of Dentistry, College of Oral Medicine, Taichung, Taiwan, [3]Chung Shan Medical University Hospital, Department of Nuclear Medicine, Taichung, Taiwan, [4]Chung Shan Medical University Hospital, Artificial Intelligence Center, Taichung, Taiwan, [5]Chung Shan Medical University, School of Medicine, Taichung, Taiwan, [6]Chung Shan Medical University, Department of Medical Informatics, Taichung, Taiwan*

**Purpose/Objectives:** Oral squamous cell carcinoma (OSCC) often arises in close anatomical relation to the jaws and may invade the bone. The presence of bone invasion significantly affects tumor staging, treatment strategy, outcome, and quality of life. This study investigated the feasibility of computerized features extracted from primary tumors on preoperative positron emission tomography (PET) images in predicting bone invasion in OSCC patients.
**Methods and materials:** Fifty-three OSCC patients who underwent immediate surgery without additional treatment between 2020 and 2022 at Chung Shan Medical University Hospital (CSMUH), Taichung, Taiwan, were included in this study, approved by the Research Ethics Committee of CSMUH. The fifty-three resected specimens containing bone slices were pathologically evaluated for bone invasion. In addition, all patients underwent preoperative PET/CT imaging for clinical staging. The maximum standard uptake value (SUVmax) was identified for each tumor on the PET image. Using the threshold of SUVmax* .4, the computerized method formed metabolic tumor volume (MTV). The radiomic features, including 11 PET parameters and 77 texture features, were then derived from MTV and evaluated for their ability to predict bone invasion using receiver operating characteristic (ROC) curve analysis. Under the ROC curve, the area's (Az) significance level was defined as 0.01.
**Result:** The characteristics of included patients were 46 males and seven females, and the mean age was 59.4. Of the 53 patients, 21 (39.6%) were confirmed as having bone invasion in the surgical specimens. In 11 PET parameters, MTV and total lesion glycolysis exhibited a significant ability to predict bone invasion (Az=.717 and .695), whereas SUVmax was not (p-value=.813). Of 77 texture features, 19 demonstrated a significant ability to predict bone invasion. The average sensitivity and specificity of these 21 predictive features were 81.4% ± 9.3% and 57.6% ± 8.6%, respectively.
**Conclusion:** The radiomic features extracted from the preoperative PET images demonstrated a significant ability to predict the presence of bone invasion in OSCC patients. Therefore, a machine-learning model that explores the relationship between the combination of computerized features and the presence of bone invasion is required and should be validated externally. This future model should assist clinicians in conducting perspicacious staging and precise assessment of resectability while designing appropriate treatment plans.

**Keywords:** Oral squamous cell carcinoma, bone invasion, PET images, radiomic

### Flat Panel Computed Tomography before functional ear surgery: is worth it?

*Authors: E. Ventura[1], R. Spasiano[2]; [1]Ente Ospedaliero Cantonale, Neuroradiology, Lugano, Switzerland, [2]Ente Ospedaliero Cantonale, Otolaryngology, Lugano, Switzerland*

**Purpose/Objectives:** High-resolution ear imaging can play an important role in the diagnostic and therapeutic planning of patients affected by hearing loss. The recent introduction of the flat-panel detector based volumetric CT, thanks to its high spatial resolution, seems to offer the otosurgeons benefits in exploring fine anatomical details of the ear beyond the limits imposed by multilayer CT. With this study we intend to preliminarily evaluate the reliability of flat-panel computed tomography (FPCT) in the diagnostic confirmation and therapeutic decision for patients with hearing loss eligible for surgical correction.
**Methods and materials:** From December 2021 to February 2023 23 patients suffering from hearing impairment with potential indication for surgical correction anticipated by thorough clinical and audiological examination underwent a FPCT scan. A C-arm angiographic system, already available at our Neuroradiology department, was adapted to achieve optimal imaging. In 16 patients the clinical-audiological suspicion was otosclerosis, 3 were clinically diagnosed with tympanosclerosis, while 4 were suffering from a severe-to-profound mixed or sensorineural hearing loss for which a cochlear implant or other prosthetic solution was foreseeable.
**Result:** Of the total 23 FPCTs, only 1 was discarded because of motion artifacts. Of 16 patients with clinical-audiological suspicion of otosclerosis, 13 had a radiologically confirmed diagnosis, while in 1 imaging oriented towards a surgically confirmed tympanic cholesteatoma with intact eardrum. In all the 3 patients with tympanosclerosis radiologic findings supported the clinical scenario. Up to now 5 patients received a stapedoplasty for otosclerosis, and 2 patients a tympanoplasty for tympanic cholesteatoma and tympanosclerosis respectively. In all these 7 operated patients the intraoperative picture was superimposable to the radiological diagnosis and in none of them the surgical inspection revealed morphological abnormalities with increased surgical risk not anticipated by imaging. All the 7 patients benefitted of an air-bone gap reduction to <15 dB. In the 4 patients affected by severe-to-profound mixed or sensorineural hearing loss, FPCT helped to early exclude inner ear abnormalities, such as ossifying labyrinth or cochlear malformations, potentially affecting implantology.
**Conclusion:** FPCT appears to be a useful tool for diagnostic confirmation, therapeutic planning and patient information in functional ear surgery, with complete concordance in our series between radiological and intraoperative data. These preliminary data encourage us to integrate it into the diagnostic routine of hearing diseases amenable of surgical correction.

**Keywords:** Flat-Panel Computed Tomography; Functional Ear Surgery

### High-spatial-resolution MRI-detected optic nerve thickening forecasts early-stage postlaminar optic nerve invasion in retinoblastoma

*Authors: C. de Bloeme, R. Jansen, P. de Graaf, M. de Jong; Amsterdam UMC, Radiology, Amsterdam, The Netherlands*

**Purpose/Objectives:** Conservative treatment methods are preferred over enucleation in retinoblastoma, which hinders assessing metastatic risk factors like postlaminar optic nerve invasion (PLONI) through histopathological examination. However, high-resolution magnetic resonance imaging (MRI) can detect subtle distal optic nerve thickening that may improve the detection of PLONI. The purpose of this study is to evaluate the accuracy of distal optic nerve thickening in predicting early-stage PLONI on MRI and develop a predictive model based on imaging characteristics to enhance diagnostic accuracy.
**Methods and materials:** This retrospective multicenter case-control study utilized high-spatial-resolution 3D T2-weighted MR images to measure the distal optic nerve (ON). Histopathology served as the reference standard for detecting PLONI. Two neuroradiologists measured the ON's width, height, and surface independently at 0, 3, and 5 mm from the ON's most distal part, while blinded to clinical data. Diagnostic accuracy was assessed using receiver operating characteristic (ROC) curves. The ON measurements that demonstrated the highest diagnostic accuracy for PLONI were incorporated into a predictive model with other MRI features.
**Result:** A total of 124 newly-diagnosed retinoblastoma patients from 2011 through 2022 were included in the study, of which 25 were identified as cases with PLONI. The best area under the curve (AUC) of 0.88 (95% confidence interval: 0.79, 0.96; $P<.001$) was achieved by using the axial optic nerve width measured at 0 mm as determined by ROC analysis. The optimal width cutoff at this level was ≥2.215 mm, with a sensitivity of 84% (95%CI: 64, 95%) and specificity of 83% (95%CI: 75, 89%) for detecting PLONI. Combining width measurements with radiological assessment of PLONI on MRI sequences improved the prediction model's sensitivity and specificity to 88% (95%CI: 69, 97%) and 92% (95%CI: 86, 96%), respectively.
**Conclusion:** Distal thickening of the optic nerve is a predictive factor for early-stage postlaminar optic nerve invasion in patients with retinoblastoma.

**Keywords:** Retinoblastoma, Optic nerve, high-spatial-resolution MRI, PLONI, metastases

### Imaging vestibular implants

Authors: A. Hedjoudje[1,2], B. Morris[3], C. Della Santina[3]; [1]Sion hospital, Sion, Switzerland, [2]University of Geneva, Geneva, Switzerland, [3]Johns Hopkins Hospital, Baltimore, United States of America

**Purpose/Objectives:** Analogous to hearing restoration via cochlear implants, vestibular function could be restored via vestibular implants that electrically stimulate vestibular nerve branches to encode head motion. This study presents the technical feasibility and first imaging results of CT for vestibular implants in 8 participants of the first-in-human Multichannel Vestibular Implant Early Feasibility Study.
**Methods and materials:** Imaging characteristics of 8 participants (3 men, 5 women; median age, 59.5 years; range, 51-66 years) implanted with a Multichannel Vestibular Implant System who underwent a postimplantation multislice CT ($n$ = 2) or flat panel CT ($n$ = 6) are reported. The device comprises 9 platinum electrodes inserted into the ampullae of the 3 semicircular canals and 1 reference electrode inserted in the common crus. Electrode insertion site, positions, length and angle of insertion, and number of artifacts were assessed.
**Result:** Individual electrode contacts were barely discernible in the 2 participants imaged using multislice CT. Electrode and osseous structures were detectable but blurred so that only 12 of the 18 stimulating electrode contacts could be individually identified. Flat panel CT could identify all 10 electrode contacts in all 6 participants. The median reference electrode insertion depth angle was 9° (range, -57.5° to 45°), and the median reference electrode insertion length was 42 mm (range, -21-66 mm). Flat panel CT of vestibular implants produces higher-resolution images with fewer artifacts than multidetector row CT, allowing visualization of individual electrode contacts and quantification of their locations relative to vestibular semicircular canals and ampullae.
**Conclusion:** As multichannel vestibular implant imaging improves, so will our understanding of the relationship between electrode placement and vestibular performance.

**Keywords:** Vestibular implants, flat panel, temporal bone

### Impact of gadopiclenol on decision making in patients with brain metastases: A post-hoc study

Authors: M. Eckl[1], G. R. Sarria[2], J. Fleckenstein[1], F. A. Giordano[1]; [1]University Medical Center Mannheim, Radiation Oncology, Mannheim, Germany, [2]University Hospital Bonn, Radiation Oncology, Bonn, Germany

**Purpose/Objectives:** Gadopiclenol (Elucirem™, Guerbet, France) is a new high relaxivity macrocyclic gadolinium-based contrast agent (GBCA), recently approved by the FDA and currently under review by the EMA. The aim of this post-hoc analysis was to evaluate the impact of contrast-enhanced MRI with gadopiclenol at 0.1 mmol/kg on decision making and radiotherapy (RT) treatment planning of brain metastases (BM).
**Methods and materials:** This is a post-hoc analysis of data from a phase IIb study where patients underwent two separate MRI examinations, one with gadopiclenol at 0.025, 0.05, 0.1 or 0.2 mmol/kg and one with gadobenate dimeglumine at 0.1 mmol/kg. MR images of patients who received both GBCAs at 0.1 mmol/kg, with ≥1 BM detected in either of the scans, were subjected to a blinded reader analysis and contouring by two radiation oncology experts. For each patient, treatment plans (stereotactic radiosurgery [SRS] or whole-brain radiotherapy [WBRT]) were calculated based on both MRIs[EM1], with the gross target volume (GTV) indicating the contrast-enhancing aspects of the tumor. Mean GTVs and healthy brain tissue receiving 12 Gy ($V_{12}$), as well as the Dice similarity coefficient (DSC) were obtained for the paired contours. The Spearman´s rank (ρ) correlation was additionally calculated. Furthermore, three experts blindly evaluated the contrast enhancement of each lesion for contouring purposes and subjectively qualified them as "better", in detriment of the counterpart, or "equal".
**Result:** In total, images from 13 adult patients were analyzed. MRI with gadopiclenol depicted additional BM as compared with gadobenate dimeglumine in 7 patients (54%). The treatment plan was changed in two patients (15%), from no treatment to SRS and from SRS to WBRT. Gadopiclenol depicted additional BM in these two patients (from 0 and 10 BM with gadobenate dimeglumine and 1 and 15 BM with gadopiclenol).
The mean GTVs and V12 were comparable between gadopiclenol and gadobenate dimeglumine (p=0.694, p=0.974). The mean DSC was 0.70 (SD: 0.14, ρ 0.82). From 36 answers, contrast enhancement was qualified as better with gadopiclenol in 21 (58.3%) evaluations, better with gadobenate dimeglumine in 8 (22.2%) evaluations, while no difference was observed in 7 (19.4%) evaluations.
**Conclusion:** Gadopiclenol at 0.1 mmol/kg improved BM detection and contrast enhancement with potential impact on RT treatment decisions.

**Keywords:** Gadopiclenol, MRI, radiotherapy, contouring, brain metastases

### Is cochlear canal measurement with MR imaging reproducible for Incomplete Partition?

*Authors:* *D. Ö. Aksoy, S. Kurt Güney, V. Gündogdu, A. S. Mahmutoglu; University of Health Sciences Turkey, Istanbul Training and Research Hospital, Radiology, Istanbul, Turkey*

**Purpose/Objectives:** In cochlear implant candidates, preoperative measurement of the cochlear canal length helps choose the appropriate electrode. Since an incomplete partition (IP) defect is a congenital anomaly, most cochlear implant candidate IP cases are in the pediatric age group. Therefore, we aimed to measure the cochlear canal length with MRI that does not include ionizing radiation and compare its reproducibility in pediatric IP cases.
**Methods and materials:** Our hospital records were scanned for cases under 18 diagnosed with IP between January 2016 and January 2023. 22 IP I, 46 IP II, and 8 IP III cochlea with MRI were included in the patient group. For the control, 50 normal cochleae were taken. Basal turn diameter (A) and width (B) of each cochlea from oblique coronal images and cochlear canal length (CCL) obtained by using 3D curved multiplanar reconstruction of Osirix software were measured by two radiologists.

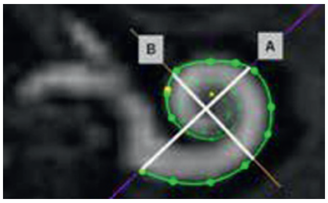

A,B and CCL measurements

Measurements of each IP type were compared with the control. Also, the intraclass correlation was assessed.
**Result:** The mean age of the patients was 6.2, and the control was 4.9. CCL and A of each IP type significantly differed from the control, and B values of IP type I and III differed.

| | | Control | IP-I | IP-II | IP-III |
|---|---|---|---|---|---|
| CCL (cm) | Mean±sd | 3.5 ± 0.2 | 2.4 ± 0.5** | 3.3 ± 0.2* | 2.4 ± 0.2** |
| A (mm) | Mean±sd | 9.2 ± 0.3 | 8.1 ± 0.9** | 9 ± 0.3* | 7.7 ± 0.8** |
| B (mm) | Mean±sd | 6.9 ± 0.3 | 5.9 ± 1.0** | 6.9 ± 0.3 | 5.8 ± 0.5** |

Kruskal-wallis (Mann-whitney u-test)

Summary of the CCL, A, and B mean values.

Except for IP type III A and B measurements, all other measurements were found to have significant intraclass correlations.

| | | $r^{ICC}$ | $p^{ICC}$ |
|---|---|---|---|
| ***CCL (cm)*** | Control | 0.744 | ***0.000*** |
| | IP-I | 0.958 | ***0.000*** |
| | IP-II | 0.804 | ***0.000*** |
| | IP-III | 0.830 | ***0.016*** |
| ***A (mm)*** | Control | 0.562 | ***0.002*** |
| | IP-I | 0.810 | ***0.000*** |
| | IP-II | 0.526 | ***0.008*** |
| | IP-III | 0.612 | 0.118 |
| ***B (mm)*** | Control | 0.766 | ***0.000*** |
| | IP-I | 0.926 | ***0.000*** |
| | IP-II | 0.665 | ***0.000*** |
| | IP-III | -0.25 | 0.611 |

$p^{ICC}$ Intra Class Correlation

Interobserver correlation

**Conclusion:** Preoperative cochlear length measurements of IP cases with MRI showed differences from the control. However, a significant intraclass correlation was found in measurements made with MRI, except for the A and B of IP type III.

**Keywords:** Cochlear canal lenght, MRI, Incomplet partition

### Magnetic Resonance Imaging in naso-oropharyngeal carcinoma: Role of texture analysis in the assessment of response to radiochemotherapy

*Authors:* *E. Bicci, L. Calamandrei, M. Pietragalla, E. Barcali, F. Mungai, L. Bonasera, C. Nardi, V. Miele; Aou Careggi, Florence, Italy*

**Purpose/Objectives:** Identifying MRI texture parameters able to distinguish inflammation, fibrosis, and residual cancer in patients with naso-oropharynx carcinoma after radiochemotherapy (RT-CHT)
**Methods and materials:** In this single-centre, observational, retrospective study, texture analysis was performed on ADC maps and post-gadolinium T1 images of patients with histological diagnosis of naso-oropharyngeal carcinoma treated with RT-CHT. An initial cohort of 99 patients was selected, 57 of them were later excluded. The final cohort of 42 patients was divided into 3 groups (inflammation, fibrosis, and residual cancer) according to MRI, 18F-FDG-PET/CT performed 3-4 months after RT-CHT, and biopsy. Pre-RT-CHT lesions and the corresponding anatomic area post-RT-CHT were segmented with 3D slicer software from which 107 textural features were derived. T-Student and Wilcoxon signed-rank tests were performed, features with $p\text{-value}<0.01$ were considered statistically significant. Cut-off values – obtained by ROC curves – to discriminate post-RT-CHT non-tumoural changes from residual cancer were calculated for the parameters statistically associated to the diseased status at follow-up.
**Result:** Two features – Energy and Grey Level Non-Uniformity – were statistically significant on T1 images in the comparison between ‘positive’ (residual cancer) and ‘negative’ patients (inflammation and fibrosis). Energy was also found to be statistically significant in both patients with fibrosis and residual cancer. Grey Level Non-Uniformity was significant in the differentiation between residual cancer and inflammation. Five features were statistically significant on ADC maps in the differentiation between ‘positive’ and ‘negative’ patients. The reduction in values of such features between pre- and post-RT-CHT was correlated with a good response to therapy.
**Conclusion:** Texture analysis on post-gadolinium T1 images and ADC maps can differentiate residual cancer from fibrosis and inflammation in early follow-up of naso-oropharyngeal carcinoma treated with RT-CHT.

**Keywords:** naso-oropharyngeal carcinoma; head and neck; texture analysis; magnetic resonance imaging; radiochemoterapy.

### Magnetic resonance imaging vs. cone beam CT in severe periodontitis: Bone marrow edema as Magnetic resonance imaging based biomarker of disease activity in severe periodontitis

*Authors: A. Lauer[1], A. Skenderi[1], L. Schulte[1], A. Juerchott[1], M. Sohani[2], F. S. Schwindling[3], P. Rammelsberg[3], S. Heiland[1], M. Bendszus[1], T. Hilgenfeld[1]; [1]Heidelberg University Hospital, Department of Neuroradiology, Heidelberg, Germany, [2]Private Practice, Private Practice, Frankfurt am Main, Germany, [3]Heidelberg University Hospital, Department of Prosthodontics, Heidelberg, Germany*

**Purpose/Objectives:** Loss of the tooth supporting alveolar bone related to chronic inflammation is a hallmark of severe periodontitis and the most frequent reason of tooth loss worldwide. Cone-beam computed tomography (CBCT) is excellent in detecting bone loss but cannot provide information regarding acute tissue inflammation. Moreover, the associated radiation dose severely limits its applicability. Dental Magnetic resonance imaging (dMRI) with its sensitivity to depict inflammation could solve both limitations of CBCT. This study aims to compare periodontal lesion size of dMRI and CBCT in terms of bone loss and bone marrow alterations in patients with periodontitis.
**Methods and materials:** In this prospective study, 19 periodontitis patients underwent pre-treatment cone-beam computed tomography (CBCT, isotopic voxel size: 0.16 mm) and a 3T dMRI (a 3D fat-saturated T2-weighted (T2W) MSVAT-SPACE sequence, 0.6 mm isovolumetric voxel size and a 3D T1-weighted gadolinium contrast enhanced, fa-suppressed (T1W+C) isotropic VIBE sequence, 0.7 mm isovolumetric voxel size). All sequences were co-registered and periodontal bone lesions were semi-automatically segmented using a multistep threshold-based algorithm for each sequence. Volumetrics were compared and correlated to clinical data.
**Result:** Altogether 216 lesions with bone loss were analyzed. While there was a strong correlation of CBCT and dMRI based lesion volumetrics (T2W vs. CBCT: $r = 0.79$, $p<0.001$ and T1W+C vs. CBCT: $r = 0.71$, $p<0.001$, Spearman correlation), lesions delineated based on T2W signal abnormalities were significantly larger compared to CBCT (CBCT vs. T2W vs. T1W+C in median cc [IQR]: 0.056 [0.033-0.139] vs. 0.082 [0.039-0.176] vs. 0.061 [0.026-0.112], Friedman test with Dunn´s correction CBCT vs. T2W/ T1W+C: $p < 0.001$/ 0.49). In 126/216 (58%) lesions, T2W signal abnormalities extended beyond the delineated bone loss by >10% (median % increase to CBCT [IQR]: 55.6 [35.8-122.9] 95% CI: 96%). These excessive [T1] T2W dMRI lesions (yes vs. no) were associated with positive bleeding on probing (89.7% vs. 60.0%, two tailed $p<0.001$, Fisher`s exact test) and deeper probing depth [OR 1.228], 95% CI: 1.038-1.453] for each 1mm increase in PD, $p = 0.016$, binomial logistic regression).
**Conclusion:** Compared to CBCT (non-invasive gold standard), dMRI identified all periodontal lesions. Bone loss in CBCBT is only slightly overestimated by T1W+C in dMRI. Bone marrow edema is closely related to active inflammation in clinical examination and may even provide an improved diagnostic accuracy over clinical examination. These results highlight the potential of dMRI for treatment planning and treatment monitoring of periodontitis.

**Keywords:** Dental Magnetic resonance imaging, severe periodontitis, bone marrow edema.

### MRI for the pre-operative prediction cochlear implant insertion depth on post-operative CBCT

*Authors: A. Shetty[1], G. Pradeep[1], I. Pai[2], S. Connor[3]; [1]King's College London, London, United Kingdom, [2]Guy's and St Thomas' Hospital, London, United Kingdom, [3]King's College Hospital, Neuroradiology, London, United Kingdom*

**Purpose/Objectives:** Cochlear implant (CI) insertion depth impacts on functional outcomes, however it is influenced by cochlea size and morphology[1]. Prediction of angular insertion depth from pre-operative MRI measurements is of great relevance since MRI is increasingly used for CI planning[2]. Applying cone beam CT (CBCT) to the evaluation of post-operative angular insertion depth also overcomes the projectional limitations of radiographic evaluation. Our primary aim was to determine if the angular depth of CI insertion on post-operative CBCT can be determined from the cochlear dimensions measured on pre-operative MRI. The secondary aims were to investigate the potential impact of electrode array type and reference electrode positioning on this prediction of angular insertion.
**Methods and materials:** This retrospective study analysed patients undergoing CI with Mid Scala or Slim J (Advanced Bionics) arrays. Two independent observers performed measurements on pre-operative MRI. Two perpendicular diameters of the basal turn (distance A and distance B) (fig 1) were measured and estimated the cochlear duct length (CDL) to 3600 (est360) and 5400 (est540) by applying the equations proposed by Escude and Schurzig[3,4]. In addition, the CDL was directly measured using a curved reformat to 3600 (dir360) and 5400 (dir540) using Osirix. Angular insertion depth and the position of the reference electrode was evaluated on the post-operative CBCT (fig 2). Intraclass correlation coefficient (ICC) was used to test inter-rater reliability. Pearsons (normally)/Kendalls tau (not normally distributed) correlation coefficients compared pre-operative MRI measures with post-operative angular insertion depth. Subgroup analysis was performed according to electrode array.
**Result:** There were 71 patients (M:F 25:46; age mean 56.5 range 2-87) and 74 ears, implanted with Mid Scala (n=35) or Slim J (n=37). The inter-rater ICC for the pre-operative measurements ranged from 0.72 (dir540) to 0.96 (distance B). There was no correlation demonstrated for the Mid-Scala electrode or measures based on diameter A/B however dir360 (0.341; $p<0.05$) and dir540 (0.31; $p<0.01$) was predictive of angular insertion depth for the Slim J electrode. A formula was derived to predict the cochlea insertion depth and the deviation from the actual angle was calculated.
**Conclusion:** Preoperative direct MRI cochlea measurements using a curved reformat to 3600 and 5400 correlated with post-operative angular insertion depth of the Slim J electrode on CBCT.

**Keywords:** cochlear implant; cone beam CT; MRI; pre-operative

References:
[1] Ketten DR, Skinner MW, Wang G, Vannier MW, Gates GA, Neely JG. , (1998), . In vivo measures of cochlear length and insertion depth of nucleus cochlear implant electrode arrays, Ann Otol Rhinol Laryngol Suppl , 175, 1-16
[2] Connor SEJ, Bell D, O'Gorman R, Fitzgerald- O'Connor A. , (2009), CT and MRI cochlear distance measurements may predict cochlear implant length required for a 360 degrees insertion. , AJNR, 1425-30
[3] Escude B, James J, Deguine O, Cochard N, Eter E, Fraysse B. , (2006), The size of the cochlea and predictions of insertion depth angles for cochlear implant electrodes, 11, 27-33
[4] Schurzig, D, Timm, ME, Batsoulis C, Salcher R , Sieber, D, Jolly C, Lenarz, T. , (2018), A Novel Method for Clinical Cochlear Duct Length Estimation toward Patient- Specific Cochlear Implant Selection. , Oto Open, 1-8

### MRI-based radiomics features of large endolymphatic sac anomaly with audiological correlation

*Authors:* *S. Parlak[1], B. Dalkıran[1], M. Ozbal Batuk[2], L. Sennaroglu[3], B. Ozgen Mocan[4], A. Akgoz Karaosmanoglu[1]; [1]Hacettepe University, Radiology, Ankara, Turkey, [2]Hacettepe University, Audiology, Ankara, Turkey, [3]Hacettepe University, Otorhinolaryngology, Ankara, Turkey, [4]University of Ilinois at Chicago, Radiology, Chicago, United States of America*

**Purpose/Objectives:** Large endolymphatic duct and sac (LEDS) is one of the most common imaging abnormalities in congenital sensorineural hearing loss. Although the exact pathogenesis is still unclear, osmotic damage of the neuroepithelium by the reflux of the hyperosmolar endolymphatic sac content through the endolymphatic duct into the labyrinth has been suggested as the cause for the hearing loss. We aimed to evaluate radiomics features on MRI in patients with LEDS anomaly and correlate with the pure-tone audiometry (PTA) and speech awareness threshold (SAT) data.
**Methods and materials:** Radiomics features based on the largest area of the enlarged endolymphatic sac which was contoured manually on axial T2-DRIVE, were extracted from each 3T temporal bone MRI study of LEDS patients. Obtained radiomics features were then correlated to the audiological data.
**Result:** The study group consisted of 35 ears (R/L:19/16) of 18 patients (F/M:10/8). Both PTA and SAT values showed significant negative correlations with large area low gray level emphasis in gray level size zone matrix (GLSZM) ($p<0.05$). Both PTA and SAT values showed positive correlations with gray level non-uniformity in gray level dependence matrix (GLDM-GLN) which was significant in PTA ($p<0.05$). Both PTA and SAT values showed negative correlations with large dependence emphasis in GLDM and sum average in gray level co-occurrence matrix (GLCM), these correlations were significant in SAT($p<0.05$).
**Conclusion:** The MRI-based radiomics nomogram correlates with hearing status in LEDS and our findings may support osmotic damage of the neuroepithelium by the reflux of the hyperosmolar sac theory and offer a perspective on future goals and objectives.

**Keywords:** radiomics, large endolymphatic duct and sac, hearing loss, temporal bone

### Obstructive salivary gland interventional radiology – how to succeed and pearls & pitfalls

*Authors:* *S. Harvey[1], A. Alade[2]; [1]Queen Victoria Hospital, East Grinstead, United Kingdom, [2]University College Hospital, London, United Kingdom*

**Purpose/Objectives:** This presentation will talk through the various treatments we offer in the treatment of obstructive salivary disease. We show our real cases, and describe case selection, equipment set up and pearls of wisdom for any delegates wishing to set up a service themselves.

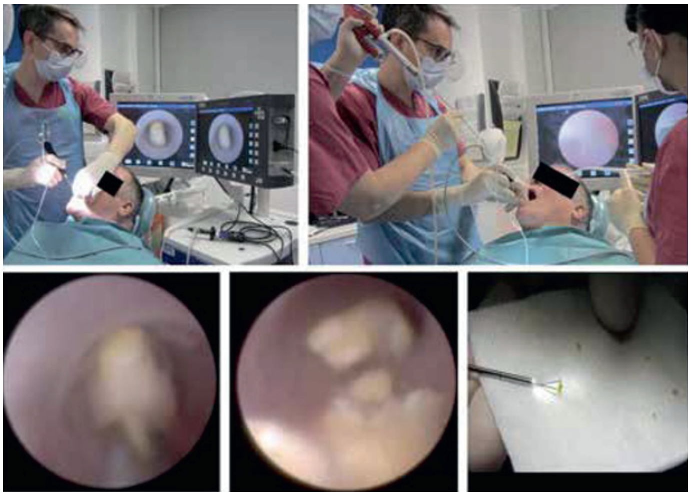

**Methods and materials:** A pictorial and video presentation of basket retrieval of stones, lithotripsy of stones, balloon dilation of strictures and Botox to atrophy glands.

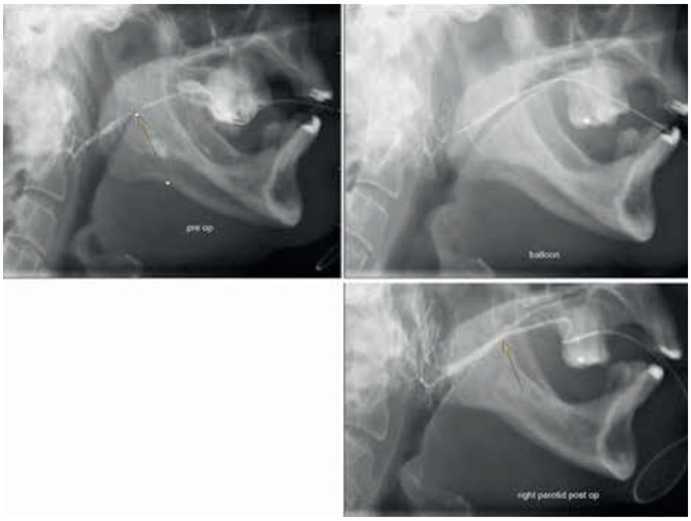

**Result:** We show how high success rates can be achieved using good case selection, safe techniques and the correct technique for each case.
**Conclusion:** These exciting treatment options for this common disease are not frequently or widely offered and after viewing this talk delegates may set up their own service locally for the benefit of their populace.

**Keywords:** Salivary, obstructive salivary disease

### Otosclerosis under MicroCT: New Insights into the Disease and its Anatomy

*Authors:* *G. O'Toole Bom Braga[1], R. Zboray[2], A. Parrilli[2], M. Bulatovic[3], M. D. Caversaccio[4], F. Wagner[5]; [1]ARTORG Center for Biomedical Engineering Research, University Bern, Bern, Switzerland, [2]Center for X-ray Analytics, Swiss Federal Laboratories for Materials Science and Technology (Empa), Center for X-ray Analytics, Swiss Federal Laboratories for Materials Science and Technology (Empa), Dübendorf, Switzerland, [3]ARTORG Center for Biomedical Engineering Research, University of Bern, ARTORG Center for Biomedical Engineering Research, University of Bern, Bern, Switzerland, [4]Department of Otorhinolaryngology, Head and Neck Surgery, Inselspital, University Hospital Bern, Department of Otorhinolaryngology, Head and Neck Surgery, Inselspital, University Hospital Bern, Bern, Switzerland, [5]University Hospital Bern, Diagnostic and Interventional Neuroradiology, Bern, Switzerland*

**Background:** Otospongiotic plaques can be seen on conventional computed tomography (CT) as focal lesions around the cochlea. However, the resolution remains insufficient to enable evaluation of intracochlear damage. MicroCT technology provides resolution at the single micron level, offering an exceptional amplified view of the otosclerotic cochlea. In this study, a non-decalcified otosclerotic cochlea was analyzed and reconstructed in three dimensions for the first time, using microCT technology. The pre-clinical relevance of this study is the demonstration of extensive pro-inflammatory buildup inside the cochlea which cannot be seen with conventional cone-beam CT (CBCT) investigation.
**Methods and materials:** A radiological and a three-dimensional (3D) anatomical study of an otosclerotic cochlea using microCT technology is presented here for the first time. 3D-segmentation of the human cochlea was performed, providing an unprecedented view of the diseased area without the need for decalcification, sectioning, or staining.
**Result:** Using microCT at single micron resolution and geometric reconstructions, it was possible to visualize the disease's effects. These included intensive tissue remodeling and highly vascularized areas with dilated capillaries around the spongiotic foci seen on the pericochlear bone. The cochlea's architecture as a morphological correlate of the otosclerosis was also seen. With a sagittal cut of the 3D mesh, it was possible to visualize intense ossification of the cochlear apex, as well as the internal auditory canal, the modiolus, the spiral ligament, and a large cochleolith over the osseous spiral lamina. In addition, the oval and round windows showed intense fibrotic tissue formation and spongiotic bone with increased vascularization. Given the recently described importance of the osseous spiral lamina in hearing mechanics and that, clinically, one of the signs of otosclerosis is the Carhart notch observed on the audiogram, a tonotopic map using the osseous spiral lamina as region of interest is presented. An additional quantitative study of the porosity and width of the osseous spiral lamina is reported.
**Conclusion:** In this study, structural anatomical alterations of the otosclerotic cochlea were visualized in 3D for the first time. MicroCT suggested that even though the disease may not appear to be advanced in standard clinical CT scans, intense tissue remodeling is already ongoing inside the cochlea. That knowledge will have a great impact on further treatment of patients presenting with sensorineural hearing loss.

**Keywords:** otosclerosis; microCT; 3D anatomical study; cochlea

### Prediction of age older than 18 years in sub-adults by MRI-segmentation of 1st, 2nd and 3rd molars

*Authors:* *M. B. B. Bjørk[1], Ø. Bleka[2], H. B. Eggesbø[3, 4], M. Haugland[3], S. I. Kvaal[1], P. M. Lauritzen[3, 5], T. Sakinis[3], F. A. Tuvnes[3]; [1]Institute of Clinical Dentistry, University of Oslo, Faculty of Dentistry, Oslo, Norway, [2]Oslo University Hospital, Department of Forensic Sciences, Oslo, Norway, [3]Oslo University Hospital, Division of Radiology and Nuclear Medicine, Oslo, Norway, [4]Institute of Clinical Medicine, Faculty of medicine, University of Oslo, Department of Radiology and Nuclear medicine, Oslo, Norway, [5]Faculty of Health Sciences, Department of Life Sciences and Health, Oslo Metropolitan University, Oslo, Norway*

**Purpose/Objectives:** We aimed to develop a method for MRI segmentation of tooth tissues in the entire 1st, 2nd and 3rd molars in order to predict the probability of a sub-adult being older than 18 years.
**Methods and materials:** We scanned 99 healthy volunteers in a 1.5 T MRI scanner with a single customised T2 sequence yielding 0.37 mm high resolution iso-voxels. The scanning time was 5 min and 4 s.
Segmentation of pulp, predentine and hard tooth tissue was performed with SliceOmatic (Tomovision©) based on MRI signal intensity thresholding, and the volumes were estimated.
Linear regression was used to analyse the association between mathematical transformation outcomes of tissue volumes, age and sex. Performance of different outcomes and tooth combinations was assessed based on the p-value of the age variable. The predictive probability of being older than 18 years was obtained by a Bayesian approach.
**Result:** 1st molars from 87 (F/M: 59/28), 2nd molars from 93 (F/M: 60/33) and 3rd molars from 67 (F/M: 45/22) participants were included. Median age was 18 years, range 14-24 years. The strongest association with age was shown for the transformation outcome (Pulp + Predentine) / Total volume for the lower right 2nd molar (M: p=9.44*10-7 / F: p=7.4*10-10) and the upper 3rd molars (Both sexes: p=3.4*10-9).
**Conclusion:** MRI segmentation of tooth tissue volumes might prove useful in the prediction of age older than 18 years in sub-adults and may be combined with other methods for age estimation in the future.

**Keywords:** Age estimation, sub-adults, molars, magnetic resonance imaging, segmentation

### Prediction of difficult round window visibility during cochlear implant surgery in matured temporal bones with pre operative CT scan: A retrospective study with radiological-surgical correlation

*Authors: S. W. Kheok[1], J. H. Ng[2], L. Liauw[1], V. Tan[2], J. Thong[3]; [1]Singapore General Hospital, Department of Diagnostic Radiology, Singapore, Singapore, [2]Singapore General Hospital, Otorhinolaryngology- Head & Neck Surgery, Singapore, Singapore, [3]Tseung Kwan O Hospital, Department of Otorhinolaryngology, Head and Neck Surgery, Hong Kong, Hong Kong*

**Purpose/Objectives:** Cochlear implant (CI) surgery is performed commonly through the facial recess via the round window (RW) approach. However, difficulty in visualising the RW could result in removal of the external auditory canal and injury to the chorda tympani. In some cases, cochleostomy may need to be performed instead. Prior studies have attempted to predict such difficult round window anatomy and each has its own limitations. In this study, we aim to identify characteristics in reformatted images of pre-operative CT temporal bones that could alert the surgeon to a potentially difficult surgery.
**Methods and materials:** In this retrospective study of 24 patients (25 ears), whose temporal bones have matured and underwent CI surgery, operative findings of RW position relative to 2nd genu and mastoid portion of facial nerve were recorded by otologists. Pre-operative CT scan images were analysed by two head and neck radiologists, in the axial and reformatted planes simulating the surgeon's view via the facial recess. The radiological assessment markers include the facial nerve to chorda tympani nerve distance (FN-CTN), the RW position relative to facial nerve (RW position) and the RW membrane's angle from the vertical axis (RW orientation).

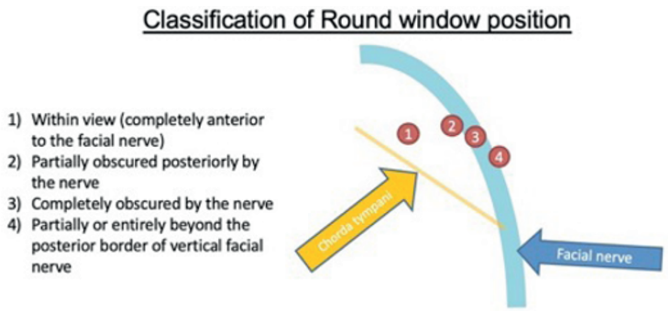

**Result:** The best predictor for difficult intraoperative RW visibility in the pre operative CT is the RW position. A RW position beyond the posterior edge of the facial nerve 2nd genu and mastoid segment had up to 60% risk of encountering difficult RW access, while those positioned more anteriorly only had a 5.0% risk of difficult access (p=0.016). There are substantial agreements in the intrarater (Kappa=0.651, p=0.001) and interrater reliability (Kappa= 0.694, p<0.001). There is no significant difference with regards to FR width (Difficult: 1.8mm mean, CI 1.4 -2.0mm; Simple: 2.1mm mean, CI 0.8-3.9) or RW orientation (difficult: 37.1° mean, CI 31.2°- 45°; Simple: 34.9° mean, CI 23.8°- 44.9°)(p>0.05).
**Conclusion:** Pre operative identification of a posteriorly positioned RW in our reformated facial recess view is a reliable and useful tool to identify and exclude potentially difficult round window access in cochlear implant surgeries.

**Keywords:** Cochlear implant, round window, facial recess, posterior tympanotomy, prediction, computed tomography

### Prognostic value of MRI-measured pre-treatment distance from the midline of squamous cell carcinoma of the oral tongue operated with compartmental surgery

*Authors: N. Di Meo[1], I. Hussain[2], M. Ravanelli[1], P. Rondi[1], A. Grammatica[3], M. Tomasoni[3], A. Bozzola[3], C. Piazza[3], D. Farina[1]; [1]Spedali Civili di Brescia, Radiology, Brescia, Italy, [2]Hospital Universiti Kebangsaan Malaysia, Radiology, Putrajaya, Malaysia, [3]Spedali Civili di Brescia, Otorhinolaryngology, Brescia, Italy*

**Purpose/Objectives:** The first purpose of this retrospective study is to assess the agreement between radiological and pathological DOI and distance from the midline (DFM). The second aim is to evaluate the prognostic value of these parameters, in order to test the assumption according to which DFM doesn't influence survival when compartmental surgery is performed, i.e. when the midline septum is spared.
**Methods and materials:** A retrospective analysis on patients affected by squamous cell carcinoma of the lateral border of the oral tongue and treated in our institution with upfront compartmental surgery from 2010 to 2021 was conducted. Availability of preoperative MRI and histological specimens, and minimal follow-up of 2 years were inclusion criteria. MRI and histological specimens were reviewed, respectively, by an experienced head and neck radiologist and a pathologist. The following data were reviewed: radiological depth of infiltration (rDOI), pathological depth of infiltration (pDOI), radiological distance from midline (rDFM), pathological distance from midline (pDFM), DOI difference (DOId) between rDOI and pDOI and DFM difference (DFMd) between rDFM and pDFM.
**Result:** Sixty-four patients were included. Median age was 61 years (IQR, 45-73.25); 68.7% were male. Preoperative MRI showed a median rDOI of 14 mm (IQR, 10-15) and rDFM of 8.5 mm (IQR, 5.87-11.5). Review of the histological slides returned a median pDOI of 13 mm (IQR, 9.92-16) and pDFM of 8 mm (IQR, 5-12). No significant differences were observed between rDOI and pDOI (p=0.321) and between rDFM and pDFM (p=0.435). A correlation with overall survival and loco regional recurrence free-survival was found only for pDOI (p<0.005), while pDFM, rDOI and rDFM didn't show any correlation with survival (p>0.005).
**Conclusion:** To the best of our knowledge, this is the first study considering DFM as possible prognostic factor in operated oral cancer patients. The absence of relationship between DFM and survival confirms the hypothesis that median lingual septum represents a valuable barrier to cancer spread even if the tumor approaches the midline, confirming the robustness of the concept of compartmental surgery.

### Pushing the boundaries of MR imaging in endodontics: an ex-vivo study comparing endodontic measurements on CBCT vs. high-resolution dental MRI using an inductively coupled intraoral coil

*Authors: T. Hilgenfeld[1], M. A. Saleem[1], F. S. Schwindling[2], U. Ludwig[3], A. C. Özen[3], J.-B. Hoevener[4], M. Bock[3], M. Nittka[5], A.-K. Eisenbeiss[6], J. Mente[7], H. Gehrig[7], S. Heiland[1], M. Bendszus[1], A. Jürchott[1]; [1]Heidelberg University Hospital, Department of Neuroradiology, Heidelberg, Germany, [2]University of Innsbruck, Prosthetic Dentistry, Insbruck, Austria, [3]University of Freiburg, Department of Radiology, Medical Physics, Freiburg, Germany, [4]University Medical Center Schleswig-Holstein, Department of Radiology and Neuroradiology, Kiel, Germany, [5]Siemens Healthcare GmbH, Magnetic Resonance, Erlangen, Germany, [6]University Medical Center Schleswig-Holstein, Department of Oral and Maxillofacial Surgery, Kiel, Germany, [7]Heidelberg University Hospital, Department of Conservative Dentistry, Heidelberg, Germany*

**Purpose/Objectives:** Due to limitations in spatial resolution or excessive examination times current dental magnetic resonance imaging (dMRI) techniques were not able to compete with cone-beam computed tomography (CBCT) for endodontic purposes. The aim of this ex-vivo study was to evaluate the diagnostic performance of novel intraoral coil-based high-resolution dMRI protocols specifically optimized for endodontic measurements under clinical conditions.
**Methods and materials:** In a realistic human tooth model performance of five different MRI-sequences (TrueFISP, FLASH, DESS, CISS, UTE) was evaluated for established endodontic measurements (pulp chamber dimensions, root canal working length, apical root canal diameter and apical dentine thickness) using CBCT as reference imaging modality. Reliability was assessed using intraclass correlation coefficients (ICCs) and accuracy using two-way analysis of variance.
**Result:** Within 6 to 8 minutes an isotropic resolution of 0.21 mm3 was achieved. Intra- and inter-rater reliability was excellent for all MRI-sequences (intraclass correlation coefficients > 0.99).
In general, MRI based measurements revealed a slight overestimation compared to CBCT, with a mean difference of 0.10±0.59 mm. For all absolute measurements combined no significant difference were noted between TrueFISP, FLASH, DESS, CISS, UTE (0.08±0.58, 0.08±0.56, 0.11±0.55, 0.15±0.56, 0.07±0.67). For individual measurements only UTE performed significantly worse for root canal diameter (0.30±0.38 mm; best sequences CISS/DESS with 0.04±0.34/0.07±0.39 mm). As a trend CISS performed worse for pulp chamber measurements (0.41±0.41 mm, mean of all sequences 0.21±0.49 mm).
**Conclusion:** Altogether, results indicate that the novel dMRI setup using a intraoral coil and endodontically optimized high-resolution 3D sequences TrueFISP, DESS or FLASH enables accurate endodontic measurements within clinically feasible acquisition times.

**Keywords:** MRI, dentistry, endodontics, dental MRI, CBCT

### Radiologic Findings in Ocular Perforating Injuries

*Authors: S. Parlak[1], O. Dikmetas[2], A. Akgoz Karaosmanoglu[1]; [1]Hacettepe University, Radiology, Ankara, Turkey, [2]Hacettepe University, Ophthalmology, Ankara, Turkey*

**Purpose/Objectives:** Eye traumas are one of the most common causes of vision loss. Radiological examinations play a role in diagnosis. The aim of this study was to retrospectively evaluate the radiologic findings of patients with ocular traumatic perforation.
**Methods and materials:** Computed tomography (CT ) findings of 20 eyes of 15 patients with traumatic ocular perforation were retrospectively examined.
**Result:** The median age was 31 years (min/max:2/86) with male predominance (M/F:11/4). Sharp and penetrating objects injuries were common. Blunt trauma injuries were rare including a case with Ehler-Danlos disease. The presence of increased periocular density (80%), volume loss (66%) and contour irregularity (66%) of bulbus oculi, intraocular hemorrhage (60%), extraocular foreign bodies (46%) were common findings. The presence of lens dislocation or rupture (30%), intraocular or scleral air (26%), and intraocular foreign bodies (20%) were less common.
**Conclusion:** Supporting the clinical findings with radiological findings is important for accurate treatment. In addition to volume and diameter reduction and increased periocular density in ocular perforations, other accompanying findings should be carefully evaluated.

### The effect of MRI-based radiomics in discriminating pediatric head and neck rhabdomyosarcoma and lymphoma

*Authors: S. Parlak, B. Dalkıran; Hacettepe University, Radiology, Ankara, Turkey*

**Purpose/Objectives:** Rhabdomyosarcoma (RMS), as one of the small blue round cell tumors, is the second most common head and neck malignancy after lymphoma in the pediatric age. The imaging characteristics of these tumors could overlap. We aimed to evaluate radiomics features in discriminating pediatric head and neck RMS and lymphoma.
**Methods and materials:** Radiomics features extraction was performed by outlining the largest area of primary tumor without necrosis, manually. First order, shape and texture features were extracted from each patient's pretreatment axial contrast-enhanced MRI.
**Result:** The study group consisted of 12 RMS and 15 lymphoma patients. 15 of 100 radiomics features (High gray level emphasis, Large dependence emphasis, Low gray level emphasis, Small dependence high gray level emphasis and Small dependence low gray level emphasis in GLDM (Gray Level Dependence Matrix); Gray level non-uniformity, Gray level variance, High gray level run emphasis, long run emphasis, long run high gray level emphasis, long run low gray level emphasis, low gray level run emphasis, run entropy and normalized run lenght non-uniformity in GLRLM (Gray Level Run Lenghts Matrix); normalized gray level non-uniformity in GLSZM (Gray Level Size Zone Matrix) were significantly different ($p<0.01$).
**Conclusion:** The MRI-based radiomics features can discriminate head and neck RMS and lymphoma.

**Keywords:** radiomics, rhabdomyosarcoma, lymphoma

### The impact of interactive radiologist peer-review on MRI-based radiotherapy target volume delineation in head and neck cancer

*Authors: D. Adjogatse[1,2], I. Petkar[1], M. Reis Ferreira[1], A. Kong[1], M. Lei[1], C. Thomas[1,2], S. F. Barrington[2,3], C. Dudau[1,4], P. Touska[1,4], T. Guerrero Urbano[1,5], S. Connor[1,2,4]; [1]Guys and St Thomas' NHS Foundation Trust, London, United Kingdom, [2]School of Biomedical Engineering and Imaging Sciences, King's College London, London, United Kingdom, [3]King's College London and Guy's and St Thomas' PET Centre, London, United Kingdom, [4]King's College Hospital, Department of Neurororadiology, London, United Kingdom, [5]King's College London, Faculty of Dentistry, Oral and Craniofacial sciences, London, United Kingdom*

**Purpose/Objectives:** Peer review of Head and Neck (HN) Radiotherapy (RT) target volumes by HN radiologists was introduced in our institution to optimise volume delineation. The focus was delineation of gross disease on Magnetic Resonance Imaging (MRI). This study assessed the impact of radiologist peer review through qualitative and quantitative analysis.
**Methods and materials:** All radical HN RT cases with a fused MRI for volume delineation were prospectively reviewed. Gross Tumour Volumes (GTV) defined by HN oncologists were reviewed by a HN radiologist in one-to-one sessions and modified as indicated. Oncologist-delineated volumes were labelled GTVonc; post-radiologist volumes were labelled GTVrad. Following peer review, Clinical Target Volumes (CTVonc, CTVrad) were created by adding a 10mm isotropic margin to each GTV-tumour (GTVT) or GTV-nodal (GTVN), then edited off anatomical barriers. The rate of change, anatomical patterns of change, and factors associated with volume discrepancy were prospectively recorded. Amended and original volumes were compared using Dice Similarity Coefficient (DSC), Jaccard Index (JI) and Hausdorff Distance (HD). 'Major' changes defined as: editing of GTV by ≥10mm, editing to avoid a GTV geographical miss, or inclusion of suspicious nodes within the high-dose CTV, were retrospectively assessed.
**Result:** 50 cases were reviewed from April 2019 - March 2020. Median age was 59 years and 72% were males. 76% of GTVTs and 41.5% of GTVNs were altered, with 54.8% of GTVT and 66.6% of GTVN alterations classified as 'major'. Undercontouring of GTVT soft tissue involvement and unidentified pathological or 'boderline-pathological' lymph nodes were predominant reasons for change. Radiologist review of gross disease on diffusion-weighted MRI was implicated in 7 GTV adjustments.
Radiologist review significantly altered the size of both the GTVT ($p=0.034$) and CTVT ($p=0.003$), but not GTVN or CTVN. The median conformity and surface distance metrics were: GTVT DSC 0.93 (0.82-0.96), JI 0.87 (0.7-0.94), HD 7.45mm (5.6-11.7mm); and GTVN DSC 0.95 (0.91-0.97), JI 0.91 (0.83-0.95) and HD 20.7mm (12.6-41.6). Conformity improved on GTVT-to-CTVT expansion (DSC 0.93 versus 0.95, $p=0.003$).
**Conclusion:** MRI-based radiologist review resulted in RT target volume amendments in the majority of patients. Despite high conformity indices, most changes to GTVT and GTVN were considered "major". Although volumetric similarities improved on GTVT-to-CTVT expansion, changes to the volume size remained statistically significant for both GTVT and CTVT. The true clinical significance of these changes remains uncertain. However the quantitative measures and descriptive reasons for volume adjustment illustrate the benefit of radiologist input for MRI-based volume delineation in HNC.

**Keywords:** Radiotherapy; Peer-review; MRI

### The spectrum of cochlear malformations in CHARGE syndrome and insights into the role of the CHD7 gene during embryogenesis of the inner ear

*Authors: F. D'Arco[1], M. Lewis[2], A. Juliano[3], C. Robson[4]; [1]Great Ormond Street Hospital, Radiology, LONDON, United Kingdom, [2]GOSH, Radiology, London, United Kingdom, [3]Mass Ear and Ear, Radiology, Boston, United States of America, [4]Boston Children's Hospital, Neuroradiology, Boston, United States of America*

**Purpose:** We reviewed the genotypes and the imaging appearances of cochleae in CHARGE patients from two large tertiary centres and analysed the observed cochlear anomalies, providing detailed anatomical description and a grading system. The goal was to gain insight into the spectrum of cochlear anomalies in CHARGE syndrome, and thus, in the role of the CHD7 gene in otic vesicle development.
**Methods:** We retrospectively reviewed CT and/or MR imaging of CHARGE patients referred to our institutions between 2005 and 2022. Cochlear morphology was analysed and, when abnormal, divided into 3 groups in order of progressive severity. Other radiological findings in the temporal bone were also recorded. Comparison with the existing classification system of cochlear malformation was also attempted.
**Results:** Cochlear morphology in our CHARGE cohort ranged from normal to extreme hypoplasia. The most common phenotype was cochlear hypoplasia in which the basal turn was relatively preserved, and the upper turns were underdeveloped. All patients in the cohort had absent or markedly hypoplastic semicircular canals and small, misshapen vestibules. Aside from a stenotic cochlear aperture (fossette) being associated with a hypoplastic or absent cochlear nerve, there was no consistent relationship between cochlear nerve status (normal, hypoplasia, or aplasia) and cochlear morphology.
**Conclusion:** Cochlear morphology in CHARGE syndrome is variable. Whenever the cochlea was abnormal, it was almost invariably hypoplastic. This may shed light on the role of CHD7 in cochlear development. Accurate morphological description of the cochlea contributes to proper clinical diagnosis and is important for planning surgical treatment options

**Keywords:** CHARGE; CHD7; Cochlea; Inner ear; Temporal bone.

### The Value of 3D FLAIR MRI in idiopathic sudden sensorineural hearing loss

*Authors: D. W. Park, S. Bae; Hanyang University Guri Hospital, Radiology, Guri-si, Republic of Korea (South Korea)*

**Purpose/Objectives:** Although 3D FLAIR MRI has been used for the diagnosis of idiopathic sudden sensorineural hearing loss (ISSNHL), the relationship of 3D FLAIR MRI findings and the prognosis of patients with ISSNHL remains controversial. This study aims to evaluate the value of 3D FLAIR MRI in patients with ISSNHL
**Methods and materials:** This study included 281 patients who presented with unilateral ISSNHL and underwent 3D FLAIR MRI. 3D FLAIR MRI findings of the patients with ISSNHL were retrospectively analyzed, according to the signal change and/or enhancement of labyrinth as followings; negative MRI findings (MRI-), positive MRI findings (MRI+) including labyrinthine enhancement as blood labyrinth barrier breakdown (BLBB), labyrinthine FLAIR hyperintensity as protein outflow disturbance, labyrinthine T1 and FLAIR hyperintensity as labyrinthine hemorrhage, and both labyrinthine enhancement and FLAIR hyperintensity as both BLBB and protein outflow disturbance. The relationship of 3D FLAIR MRI findings, the degree of hearing loss and the prognosis of patients with hearing loss were statistically analyzed with the results of audiometry and vestibular function tests.
**Result:** 3D FLAIR MRI findings in the patients with ISSNHL were divided into MRI- (n = 162) and MRI+ (n = 119), and MRI+ included BLBB (n = 54), protein outflow disturbance (n = 15), labyrinthine hemorrhage (n = 11), and both BLBB and protein outflow disturbance (n = 39). As a whole, 3D FLAIR MRI findings in the patients with ISSNHL were classified into 5 groups.
Among 5 groups, hearing loss, duration of treatment, vestibular dysfunction were statistically different ($p < 0.05$), respectively. The degree of hearing loss and duration of treatment in labyrinthine hemorrhage group was significantly severe comparing to that in the other groups ($p < 0.05$).
**Conclusion:** 3D FLAIR MRI findings were correlated with the degree and prognosis of hearing loss in the patients with ISSNHL, as the sensitive MRI biomarker.

**Keywords:** idiopathic sudden sensorineural hearing loss (ISSNHL); three-dimensional fluid-attenuated inversion recovery sequence magnetic resonance imaging (3D FLAIR MRI); labyrinthine enhancement; labyrinthine FLAIR hyperintensity

### Two or more different lesions found in the head and neck

*Authors:* *S. Jung*[1], *M. Lee*[1], *S.-w. Park*[2]; [1]*The Catholic University of Korea, Radiology, Seoul, Republic of Korea (South Korea),* [2]*College of Medicine, Seoul National University, Radiology, Seoul, Republic of Korea (South Korea)*

**Purpose/Objectives:** To evaluate the imaging findings of the patients with two or more different lesions found in the head and neck for correct diagnosis and treatment.
**Methods and materials:** We evaluate the four patients with two or more different lesions found in the head and neck. Case 1: A 48-year old male was diagnosed with three different diseases; recurrent hypopharyngeal cancer, tuberculous lymphadenitis, and thyroid papillary thyroid cancer. Case 2: A 65-year old male was diagnosed with two different diseases: glottic squamous cell carcinoma and large B cell lymphoma. Case 3: A 75-year old male was diagnosed with organizing hematoma of the maxillary sinus and tuberculous lymphadenitis. Case 4: A 31-year old female was diagnosed with tuberculous lymphadenitis and lymph node metastasis for thyroid papillary thyroid cancer.
**Result:** Three patients simultaneously had tuberculous lymphadenitis and other disease. Two out of three patients were diagnosed with thyroid papillary thyroid carcinoma concurrently with tuberculous lymphadenitis. Both tuberculous lymphadenitis and thyroid papillary thyroid carcinoma metastatic lymph node can show rim enhancing and centrally necrotic lymph node with calcification. One patient showed glottic mass and multiple enlarged bilateral cervical lymph nodes on CT scan. The bilateral multiple large lymph nodes showed homogeneously mild enhancement unlike metastatic lymph node from glottic carcinoma.
**Conclusion:** Although it is not common to be diagnosed with two or more lesions in the head and neck, the possibility should be kept in mind for correct diagnosis and treatment.

### Ultra-High Resolution CT of the head and neck with deep learning reconstruction – assessment of image quality and radiation exposure and intraindividual comparison with normal-resolution-CT

*Authors:* *S. Altmann*[1], *M. Abello Mercado*[1], *F. Ucar*[1], *A. Kronfeld*[1], *B. Al-Nawas*[2], *A. Mukhopadhyay*[3], *C. Booz*[4], *M. Brockman*[1], *A. Othman*[1]; [1]*University Medical Center Mainz, Department of Neuroradiology, Mainz, Germany,* [2]*University Medical Center Mainz, Department of oral and maxillofacial Surgery, Mainz, Germany,* [3]*Technical University of Darmstadt, Department of Computer Science, Darmstadt, Germany,* [4]*University Clinic Frankfurt, Department of diagnostic and interventional Radiology, Frankfurt, Germany*

**Purpose/Objectives:** To assess subjective and objective image quality, as well as radiation dose of ultra-high resolution CT (UHR-CT) with deep learning-based image reconstruction engine (AiCE) and intraindividually compare it to normal resolution-CT (NR-CT)
**Methods and materials:** In this retrospective study, 40 consecutive patients who underwent repetitive head and neck UHR-CT with deep learning-based image reconstruction engine (AiCE) for clinically diagnosed head and neck malignancies and available prior NR-CT of a different scanner with different focal spot size and image matrix. Subjective image quality was evaluated by two readers on a 5-point Likert-scale regarding image noise, image sharpness, artifacts and diagnostic acceptability, as well as assessability of various anatomic regions. To ensure reproducibility, the inter-Reader agreement was analyzed. For objective image evaluation, signal to noise-ratio (SNR), contrast to noise-ratio (CNR) and the slope of the gray-value transition between different tissues was calculated. The radiation dose was evaluated by comparing CTDIvol, DLP and mean effective dose values.
**Result:** UHR-CT with AiCE reconstruction led to a significant improvement in subjective (e.g. image noise and diagnostic acceptability: $p<0.000$; inter-rater agreement: ICC $\geq 0.91$) and objective image quality (e.g. SNR: $p<0.000$ and CNR: $p<0.025$) at significantly lower radiation doses (NR-CT $2.03 \pm 0.14$ mSv; UHR-CT $1.45 \pm 0.11$ mSv; $p<0.0001$) as compared to NR-CT.
**Conclusion:** Compared to NR-CT, UHR-CT with AiCE provides superior image quality at lower radiation dose. Thus, UHR-CT may further improve the role of CT in the assessment of head and neck pathologies.

**Keywords:** computed tomography, head and neck neoplasms, ultra-high resolution, image quality, radiation dose, deep learning, image reconstruction

### Ultrasound-guided Radiofrequency ablation of Parotid Warthin's tumour – Safety and Feasibility Study

*Authors:* *H. S. Leung[1], D. C. M. Yeung[2], R. Lai[2], A. K. F. Lee[2], E. W. Y. Wong[2], E. H. L. Lau[2], J. Y. K. Chan[2], K. T. Wong[1]; [1]The Chinese University of Hong Kong, Department of Imaging and Interventional Radiology, Hong Kong, Hong Kong, [2]The Chinese University of Hong Kong, Department of Otorhinolaryngology, Head and Neck Surgery, Hong Kong, Hong Kong*

**Purpose/Objectives:** Warthin's tumour is the second most common benign tumour of parotid glands which tend to involve middle-to-old age male smokers. Surgical parotidectomy is the traditional treatment of choice but associated with significant complications including transient or even permanent facial nerve palsy. While ultrasound (USG)-guided radiofrequency ablation (RFA) is minimally invasive and could achieve tumour shrinkage and symptomatic improvement, its efficacy is mainly proven in benign thyroid nodules or other visceral malignancies with limited data on treatment of Warthin's. This study is to evaluate the safety and feasibility of RFA on treatment of parotid Warthin's.
**Methods and materials:** This is a single-centre, institutionally approved, single-arm non-blinded clinical trial. 20 consecutive patients with Warthin's tumour of the parotid gland superficial lobe were recruited and treated with USG-guided RFA of the tumour under local anaesthesia with subsequent post-procedural follow-up visits of up to 45 weeks. The primary outcome was reduction of the volume of parotid tumour based on serial USG measurements when compared with baseline, with secondary outcomes on serial reduction of tumour size and cosmetic symptoms during each follow-up visit. Safety outcomes were evaluated on complications including facial paralysis, with comparison against a case-control matched cohort of surgical parotidectomy patients based on age, sex and tumour volume measurements. Statistical analysis performed with Wilcoxon signed-rank and chi-square tests.
**Result:** 19 patients were included in the final analysis. At a median of 45 weeks post-procedure there was a 68.4% volume reduction compared to baseline (p<0.001). When compared with baseline, there is significant improvement in cosmetic score from 12 weeks onwards (p=0.014 at 12 weeks). 3 patients had transient facial nerve palsy (all recovered by 12 weeks post-procedure), and 3 patients had numbness of the great auricular nerve. When compared with the historical cohort of age and sex-matched parotidectomy patients, there is a lower incidence of great auricular nerve numbness (p<0.001) with no significant difference in facial nerve paralysis (p=0.11). RFA is also associated with shorter procedure time and length of stay when compared with surgery (both p<0.001).
**Conclusion:** USG-guided RFA is a safe alternative to surgical parotidectomy for treatment of Warthin's tumour, offering significant volume reduction and improvement of symptoms with a comparable safety profile, shorter procedure time and length of stay.

**Keywords:** Radiofrequency Ablation, Warthin's tumour, Parotid gland, Interventional Radiology

### Visualization and Clinical Relevance of the Endolymphatic Duct and Sac in Ménière's Disease

*Authors:* *L. de Pont[1,2], M. Houben[3], T. Verhagen[3], B. Verbist[2], M. van Buchem[2], C. Bommeljé[4], H. Blom[4], S. Hammer[1]; [1]Haga Teaching Hospital, Radiology, The Hague, The Netherlands, [2]Leiden University Medical Center, Radiology, Leiden, The Netherlands, [3]Haga Teaching Hospital, Radiology, Otorhinolaryngology, The Hague, The Netherlands, [4]Haga Teaching Hospital, Otorhinolaryngology, The Hague, The Netherlands*

**Purpose/Objectives:** To investigate (1) to which extent non-visualization of the endolymphatic duct-sac (ED-ES) system is a radiological feature of Ménière's disease (MD) in a cohort of vertigo-associated pathologies (VAP); and (2) if visible, whether different angular trajectories of the ED-ES system in MD are characterized by distinguishable clinical features.
**Methods and materials:** We retrospectively assessed 301 patients (187 definite MD, 114 other VAP) that underwent 4h-delayed gadolinium-enhanced 3D FLAIR MRI at our vertigo referral center (Haga Teaching Hospital, The Hague, The Netherlands) between February 2017 and March 2019. We evaluated (1) the visibility of the ED-ES system between definite MD and other VAP patients, and (2) measured the angular trajectory of the ED-ES system in MD patients with a visible ED-ES complex using open-source software[4]. MD patients were stratified based on the angular measurements into $\alpha_{exit} \leq 120°$ (MD-120), $\alpha_{exit}$ 120°-140° (MD-intermediate) or $\alpha_{exit} \geq 140°$ (MD-140). Correlations between MD subgroups and clinical parameters were evaluated.

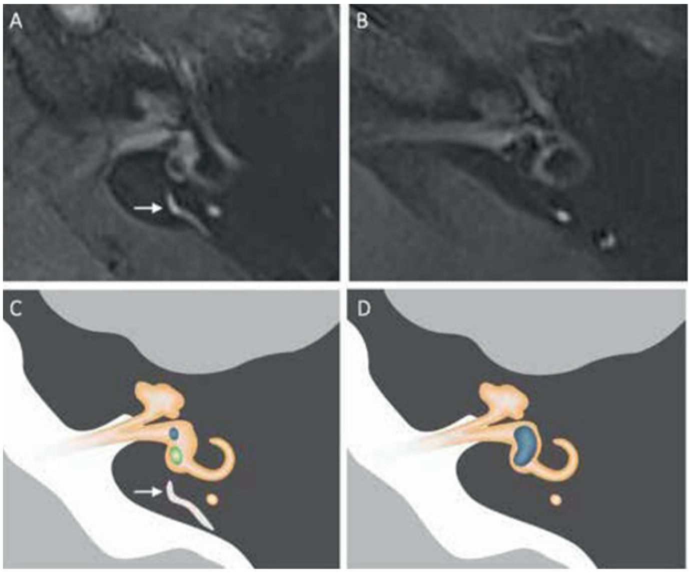

A, visualization of the ED-ES system (long arrow) in a patient diagnosed with vestibular migraine. B, non-visualization of the ED-ES system in patient with definite MD. C and D, corresponding schematic depiction.

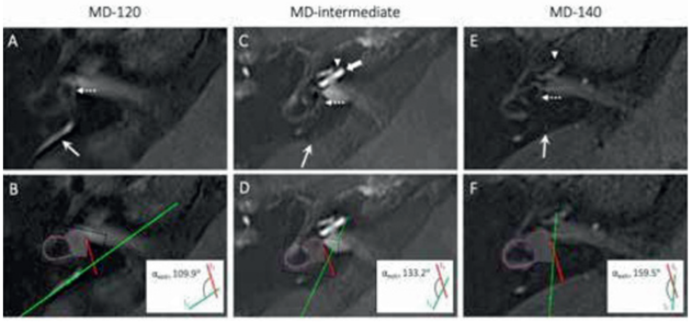

Angular trajectory of the ED-ES system in definite MD cases with MD-120 (A, B), MD-intermediate (C, D) and MD-140 (E, F) morphologies. (A, C, E) Gd-enhanced FLAIR MRI, the long arrows indicate the ED-ES system in the opercular region. (B, D, F) show the corresponding $\alpha$ exit.

**Result:** Non-visualization of the ED-ES system was more common in definite MD patients compared with other VAP (P<.001). Among definite MD patients, the MD-140 subtype demonstrated a longer history of vertigo (P=.006), a higher prevalence of bilateral clinical disease (P=.005), and a trend towards a male

| | Definite MD | Other VAP | P |
|---|---|---|---|
| Visibility of the ED-ES system | | | <.001 |
| Visible, n (%) | 162 (79.4) | 161 (96.4) | |
| Non-visible, n (%) | 42 (20.6) | 6 (3.6) | |

Table 1. Visibility of the ED-ES system

| | Data available, n | MD-120 (n=69) | MD-intermediate (n=29) | MD-140 (n=16) | P |
|---|---|---|---|---|---|
| **Female:male ratio** | 114 | 44:25 | 15:14 | 5:11 | 0.053 |
| **Unilateral:bilateral MD ratio** | 114 | 63:6 | 29:0 | 11:5 | 0.005 |
| **Median low Fletcher, dB** | 97 | 48 [2; 108] | 53 [13; 75] | 45 [23; 120] | 0.900 |
| **Median high Fletcher, dB** | 97 | 49 [5; 108] | 52 [18; 75] | 50 [28; 120] | 0.977 |
| **Median vertigo disease duration, y** | 80 | 2.8 [0.1; 30.7] | 4.9 [1.1; 11.0] | 16.0 [0.4; 29.0] | 0.006 |
| **Mean vertigo attack frequency, nr per month** | 70 | 6.3 ± 6.9 | 8.2 ± 10.8 | 5.0 ± 8.8 | 0.486 |
| **Tinnitus, n (%)** | 83 | 39 (83.0) | 24 (96.0) | 10 (90.9) | 0.121 |
| **Aural fullness, n (%)** | 64 | 33 (89.2) | 17 (89.5) | 8 (100) | 1.000 |
| **Drop attacks, n (%)** | 49 | 2 (6.3) | 1 (11.1) | 1 (12.5) | 0.432 |

Table 2. Correlations between MD subgroups (n=114)

preponderance (p=.053). No significant differences were found between MD subgroups regarding the degree of sensorineural hearing loss, the presence of tinnitus or aural fullness, or the frequency of vertigo attacks.
**Conclusion:** Non-visualization of the ED-ES system is significantly associated with MD. Among MD patients with a visible ED-ES system, we demonstrated that the MD-140 subtype is associated with a longer disease duration, a higher prevalence of bilateral MD, and a trend towards a male preponderance.

**Keywords:** Meniere, MRI, endolymphatic sac, clinical features

References:
[1] Eckhard AH, et al., (2019), Inner ear pathologies impair sodium-regulated ion transport in Meniere's disease, Acta Neuropathol, 343–57, 137
[2] Bächinger D, et al. , (2019), Vestibular Aqueduct Morphology Correlates With Endolymphatic Sac Pathologies in Menière's Disease—A Correlative Histology and Computed Tomography Study, Otol Neurotol, e548–55, 40
[3] (2019), Bächinger D, et al. Endotype-phenotype patterns in Meniere's disease based on gadolinium-enhanced MRI of the vestibular aqueduct, Front Neurol, eCollection., 10
[4] Zürrer D. , CoolAngleCalc. , Https//danielzuerrer.github.io/Cool

### Which internal auditory meatus vascular loops are associated with pulsatile tinnitus?

*Authors: M. Chong[1], K. Stephenson[1], M. Sultani Tehrani[2], I. Pai[3], S. Connor[1];*
*[1]Guy's and St Thomas' NHS Foundation Trust, Department of Radiology, London, United Kingdom, [2]King's College London, School of Medicine, London, United Kingdom, [3]Guy's and St Thomas' NHS Foundation Trust, Department of Otolaryngology, London, United Kingdom*

**Purpose/Objectives:** Previous investigators have proposed that there is a relationship between vascular loops extending deep into the internal auditory canal (IAC) and pulsatile tinnitus (PT) (1,2). Unlike continuous tinnitus, the relationship between IAC vascular loops and PT has not been thoroughly investigated in a larger cohort. The primary aim of this study is to examine whether MRI evidence of vascular loops within the IAC correlates with the presence of unexplained PT, whilst the secondary aim was to determine whether there are other anatomical or audiological causes for the presence of PT in ears with IAC vascular loops.
**Methods and materials:** This retrospective study analysed consecutive patients undergoing thin section T2-w imaging for the investigation of PT. Appropriate studies were retrieved through a Boolean search of the radiology management system between 2012-22 with subsequent de-duplication. Exclusion criteria were other causes of PT on imaging, conductive hearing loss, abnormal otoscopy, significant intracranial pathology, ear trauma or surgery, and other causative medical conditions. MRI scans were scored according to the Chavda classification (graded 1 to 3). For ears with IAC vascular loops (Chavda 2 and 3), additional variables were documented: distance between apex of loop and fundus, angulation of the loop, whether the vascular loop contacted bone, whether there was semicircular canal dehiscence, and whether hyper-bone conduction was present on audiogram. The results of the Chavda classification (3) were compared for the symptomatic vs asymptomatic ears for unilateral PT ears and all ears using the Chi sq test ($p<0.05$). In ears with intra-meatal vascular loops (Chavda 2 and 3), it was determined whether the presence of PT could be accounted for by the presence of the previously documented co-variates (Chi sq, Wilcoxon signed-rank test, Mann-Whitney U test).
**Result:** There were 1501 studies retrieved from the initial search and following de-duplication and exclusions, there were 520 patients (mean age 48.3, range 12.3-93.1) and 1040 ears included in the study. A preliminary analysis (n=91) demonstrated no significant relationship between the Chavda grade and the presence of pulsatile tinnitus (Chi sq 2.61 p=0.27). The complete analysis will be presented together including any significant co-variates that contribute to the presence of PT in ears with IAC vascular loops.
**Conclusion:** This represents the first analysis of IAC vascular loops (according to the Chavda classification) in a large cohort with documented unexplained pulsatile tinnitus. We have also explored whether there are particular anatomical and audiological co-variates which account for the presence of PT in patients with IAC vascular loops.

**Keywords:** Pulsatile tinnitus, vascular loop, internal auditory canal, internal auditory meatus, Chavda classification

References:
[1] V Nowé, D De Ridder, P H Van de Heyning, X L Wang, J Gielen, J Van Goethem, O Ozsarlak, A M De Schepper, P M Parizel, (2004), Does the location of a vascular loop in the cerebellopontine angle explain pulsatile and non-pulsatile tinnitus?, European Radiology, 2282–2289, 14, https://doi.org/10.1007/s00330-004-2450-x
[2] D De Ridder, L De Ridder, V Nowé, H Thierens, P Van de Heyning, A Møller, (2005), Pulsatile Tinnitus and the Intrameatal Vascular Loop: Why Do We Not Hear Our Carotids?, Neurosurgery, 1213-7, 57(6), https://doi.org/10.1227/01.neu.0000186035.73828.34
[3] A-L McDermott, S N Dutt, R M Irving, A L Pahor, S V Chavda, (2003), Anterior inferior cerebellar artery syndrome: fact or fiction, Clinical Otolaryngology & Allied Sciences, 75-80, 28(2), https://doi.org/10.1046/j.1365-2273.2003.00662.x

### Scientific Posters

### A systematic review of artificial intelligence (AI) models in the detection of maxillofacial fractures on plain film radiography

*Author: V. Chavda; University of Birmingham, School of Dentistry, Birmingham, United Kingdom*

**Purpose/Objectives:** The purpose of this systematic review was to evaluate the current performance and capabilities of Artificial Intelligence (AI) algorithms in the detection of fractures of the facial bones, on plain film radiography only.
Objectives:
- To review the current literature and evidence which may impact the radiology workflow
- To identify any sources of bias in the current research
- To inform future research and guide further studies into the use of AI in facial fracture detection.

**Methods and materials:** A systematic search was conducted of 3 medical literature databases, as well as hand searches of 5 relevant radiology journals for head and neck imaging. Eligible studies were selected, and abstracts reviewed. Predefined inclusion and exclusion criteria allowed the selection of relevant studies for full text review. An assessment of the study quality was undertaken using the STARD-AI tool (Boa_Image_Frames for Reporting Diagnostic Accuracy Studies - Artificial Intelligence)
**Result:** A total of 32 original research papers were identified of which 5 fit the inclusion criteria. The 5 papers were based solely on the detection of mandible fractures on panoramic radiographs. Many research papers were excluded as they were based on data derived from cross sectional imaging modalities. All studies used a variation of a convolutional neural network (CNN), with varying sizes of control and fracture data sets. Variations of CNNs included YOLO and U-Nets. The summary receiver operating characteristic curve (AUC) ranged from 0.977 to 0.85 between the studies. Many studies were limited by the small sample sizes, lack of diversity in fracture detection and domain shift limiting applicabilty.
**Conclusion:** The inital selection criteria for imaging in the clinical scenario of a suspected facial fracture is often plain radiography, due to its ease of accessibility, low cost and lower comparative radiation dose. The current literature in the detection of fractures of the facial skeleton with AI is limited to panoramic radiographs of the mandible, with majority of research on cross sectional imaging modalities and little consideration of the midface fractures. The AI methodology currently used in the has been shown to be effective with a good diagnostic accuracy. The current accuracy calculations are based on a single view of a suspected fracture, whereas standard practice is to undertake two orthogonal views. The secondary view has not been considered and may input further information allowing the detection to be further increased in accuracy.
Facial fractures are often difficult to diagnose on plain film imaging due to superimposed anatomical structures and tomographic techniques. There is a role for AI in aiding clinical diagnosis and further research into its use is suggested.

**Keywords:** Plain film, Review, Artificial Intelligence, Fracture

### Acute invasive fungal sinusitis involving orbital apex: CT, MR imaging features of 6 patients

*Authors: Y.-K. Ihn, J.-h. Shin; St.Vincent's hospital, Radiology, Suwon, Republic of Korea (South Korea)*

**Purpose/Objectives:** Acute invasive fungal sinusitis (AIFS) is a life threatening disease that is difficult to diagnoses. Extension beyond the sinuses is one of the most important indicators suggesting fungal etiology. Early and accurate radiological diagnosis with expeditious surgical intervention is pivotal in improving the mortality & morbidity. The purrpose of our study was to present MR imaging features of 6 patients of acute invasive fungal rhinosinusitis involving orbital apex.
**Methods and materials:** CT and MR images and clinical records of 6 patients with AIFS were retrospectively evaluated to identify the imaging feature of various fungal etiology.
**Result:** Three patients (3/6, 50%) were immunocompromised because of poorly controlled diabetes mellitus. The associated fungal infections were aspergillosis in 3 patients (50%) and mucormycosis in 3 patients (50%). All cases showed extra-sinonasal involvement and orbit was the most common (100%, 6/6) location. The lesion enhancement pattern was classified into lack of contrast enhancement (33%, 2/6) and homogenous (17%, 1/6) and heterogenous (50 %. 3/6) enhancement. Adjacent sinus bony change pattern was classified into sclerosis (66%,4/6), destruction (17%, 1/6) and normal (17%, 1/6).
**Conclusion:** AIFS showed frequent extra-sinonasal involvement and variable MR enhancement pattern. Heterogenous enhancement pattern of involved sinus was seen about half of the case and sclerotic bony changes are predominantly seen.

**Keywords:** acute invasive fungal sinusitis, orbital apex syndrome

### Agenesis of the Internal Carotid Artery: Evaluation of Associated Vascular Anomalies and Collateral Patterns

*Authors: B.-s. Kim, M.-K. Seo, I.-A. Shin, J. Y. Lee, J. Jang, K.-J. Ahn; Seoul St.Mary's Hospital, The Catholic University of Korea, Radiology, Seoul, Republic of Korea (South Korea)*

**Purpose/Objectives:** Agenesis of the internal carotid artery (ICA) is an exceedingly rare vascular anomaly that may be associated with other vascular pathologies such as intracranial aneurysm, rete aneurysm, and arteriovenous fistula. This study aims to investigate the clinical manifestations, radiographic features, associated vascular diseases, and collateral circulation patterns in patients with ICA agenesis.
**Methods and materials:** We conducted a retrospective analysis of patients who underwent digital subtraction angiography (DSA) at a single institute over a 13-year period. Cases with ICA anomalies were identified, and their clinical and radiographic data were evaluated.
**Result:** Ten patients [median age 59 years (range, 24–75 years), 5 male and 5 female] were included in the study. DSA revealed ICA agenesis on the right side in 6 cases and on the left side in 4 cases. All cases exhibited absent or very small carotid canals at the base of the skull. Collateral circulation through the circle of Willis was observed in all cases (posterior communicating artery in 8 cases, anterior communicating artery in 7 cases). Seven cases were complicated by aneurysms, one by arteriovenous fistula, and one by ischemia. In one patient with right ICA agenesis, concurrent agenesis of the right brachiocephalic artery was observed.
**Conclusion:** ICA agenesis is a rare vascular anomaly that is frequently associated with vascular disease at hemodynamic collateral routes. We report a series of 10 cases of unilateral internal carotid artery agenesis with discussion of embryogenesis, clinical presentation, and imaging findings.

**Keywords:** Internal carotid artery, agenesis, vascular anomaly, collaterals

### CBCT in the assessment of radicular and dentigerous cysts

*Authors: A. Inzerillo[1], M. De Angelis[1], E. Palizzolo[1], E. Bruno[1], F. Bencivinni[2], G. La Tona[1], A. Lo Casto[1]; [1]Sezione di scienze radiologiche, BIND, Università degli studi di Palermo, AOUP P. Giaccone, Palermo, Italy, [2]UOS Radiologia Odontoiatrica e Maxillo-Facciale, AOUP P. Giaccone, Palermo, Italy*

**Purpose/Objectives:** CBCT is currently used for the assessment of cystic lesions in the jaws, thanks to 3D superior information with respect to conventional radiography. Cystic lesions, as radicular and dentigerous ones, may induct various alterations in the jaws, depending on location and size, and effect on surrounding teeth and structures, requiring different type of treatment. An analysis of a patient series with radicular and dentigerous cysts, studied by CBCT, aimed to define eventually associated alterations of the jaws is presented.
**Methods and materials:** 171 patients, 81 males and 90 females, age range 8-90 (average 53) yrs. studied by CBCT (120 kV, 5mA, FOV 8x5cm to 23x17cm, DAP 611mGy x $cm^2$- 877mGy x $cm^2$) in 2010-2023, were retrospectively analyzed. A dental software was used, recording location, size, type -i.e. radicular, dentigerous, residual radicular cysts and alterations in adjacent teeth and other surrounding structures.
**Result:** 178 cysts were detected, 81 (47.4%) in males, 90 (52.6%) in females. 107/178 (60.1%) cysts were located in the maxilla and 71 (39.9%) in the mandible. 156/178 were radicular cysts (87,7%), 16/178 were dentigerous cysts (8,9%), 6 were residual radicular cysts (3,4%). Dentigerous cysts included 38 in 6/16, 48 in 2/16, 18 in 2/16, 26 in 1/16, 28 in 1/16, 34 in 1/16, 43 in 1/16, 46 in 1/16, 47 in 1/16. Radicular cysts diameter was 5-34 mm (average 9.3 mm). Dentigerous cysts diameter was 7-38 mm (average 14.9 mm). Residual radicular cysts diameter was between 9-21 mm (average 10 mm). Thinning and expansion of the bone cortex was observed in 34/178 (19.1%) cysts, lifting of the floor of the maxillary sinus in 21/178 cysts (11.8%), dislocation of the mandibular canal in 8/178 (4.5%) cysts and dislocation of contiguous teeth in 3/178 (1.7%) cysts.
**Conclusion:** CBCT is important to assess the location, size and precisely define topographic relation of radicular and dentigerous cysts prior to treatment, especially in larger ones that cause dislocation of the teeth or other contiguous anatomical structures, such as the nasal fossa, maxillary sinus or mandibular canal.

### Compensation of batch effect in ultrasonography radiomics of normal submandibular gland for the multi-center studies

*Authors: Y. J. Choi, K. J. Jeon, C. Lee, S.-S. Han; Yonsei University, College of Dentistry, Oral and Maxillofacial Radiology, Seoul, Republic of Korea (South Korea)*

**Purpose/Objectives:** This study aimed to apply the machine learning method, ComBat harmonization, to the radiomics features and investigate its effectiveness to compensate for the batch effect in normal submandibular glands of ultrasonography.
**Methods and materials:** Thirty ultrasound images of normal submandibular glands acquired with two different ultrasound devices and imaging parameters of healthy adults between the ages of 20 and 40 were retrospectively chosen. A radiologist with more than 7 years of experience determined the whole parenchyma of the normal submandibular gland as the region of interest and extracted 103 radiomics features using A-VIEW (Coreline Soft Inc., Seoul, Republic of Korea). Features showing low variability in two groups were selected as reliable radiomics features for the decision of normal submandibular gland. The median and distribution of reliable features between the two groups were statistically compared before and after applying the ComBat algorithm using Mann-Whitney U-test ($p<0.05$).
**Result:** In a total of 103 features, 17 features had low variability, in both groups with normal salivary glands. The reliable features were one histogram, 11 GLCMs, 2 GLDM, 2 GLRLM, and one GLSZM. Before applying the normalization algorithm, all reliable features except for two (Histogram Entropy and GLCM Maximal correlation coefficient) had significant differences between the two groups. After the application of the ComBat harmonization method, the median and distribution of the 16 features were harmonized to show no significant difference between the two groups ($p>0.05$). One feature (GLCM Inverse Variance) remained different ($p\leq0.05$).
**Conclusion:** Radiomics features were different even in sonographic images of normal submandibular glands due to the differences in devices and imaging protocols in multi-center studies. This batch effect can be a major obstacle to radiomics research using ultrasound images. However, the harmonization method using machine learning will improve the accuracy of the diagnostic model, which will be helpful for large-scale, multicenter studies.

**Keywords:** Ultrasonography, Salivary gland, Radiomics, Machine Learning

### Development of deep learning based volumetry of olfactory bulb from high-resolution MRI

*Authors: M. K. Lee[1], Y. M. Lim[2], S. Jung[3], J. H. Baek[3], J. H. Lee[3]; [1]Yeoido St. Mary's Hospital, College of Medicine, The Catholic University of Korea, Radiology, Seoul, Republic of Korea (South Korea), [2]Asan Medical Center, Neurology, Seoul, Republic of Korea (South Korea), [3]Asan Medical Center, Radiology, Seoul, Republic of Korea (South Korea)*

**Purpose/Objectives:** To develop a deep learning model for automatic segmentation and volumetry of olfactory bulb from high-resolution MRI and to test the model in three separate patient groups with cognitive dysfunction, diabetes mellitus, or olfactory dysfunction
**Methods and materials:** Total 432 patients who underwent brain MRI with high resolution T2WIs were enrolled and divided into training (n = 214), internal validation (n = 120), and external validation (n = 98) sets. We trained and validated a nnU-Net deep learning model for segmentation of olfactory bulb using manually segmented volume as the ground truth. To test the developed model, we enrolled normal control (n = 30), patients with mild cognitive impairment (MCI, n = 30), Alzheimer's disease (AD, n = 30), and diabetes mellitus (DM, n = 30) in the internal validation set. External validation set was composed of those with olfactory dysfunction (n = 64) and control (n = 34). Among cognition groups, One-way ANOVA with post-hoc Bonferroni correction was used for comparison of continuous variables and Kruskal-Wallis test with post-hoc Bonferroni correction was used for comparison of nominal variables. Within DM group and external validation set, Student *t* test or Mann-Whitney test were used for comparison of continuous variables and $\chi^2$ test was used for comparison of nominal variables.
**Result:** The prediction volumes of olfactory bulb in training set, internal validation set, and external validation set were 39.2 ± 11.1 $mm^3$, 37.2 ± 10.3 mm3, and 30.0 ± 10.3 $mm^3$, respectively. The mean dice scores of training, internal validation, and external validation sets were 87.0 ± 8.3%, 84.5 ± 12.4%, 87.1 ± 10.8%, respectively. Volumetry by deep learning model revealed no significant difference among control, MCI, and AD groups. The mean dice scores of each group ranged from 83.2 to 88.0 %. DM patients showed lager olfactory bulb volume than control (37.8 ± 9.9 $mm^3$ vs. 36.3 ±11.2 $mm^3$), but there was no statistical significance. The mean dice scores of each DM group ranged 71.5 – 89.6%. Olfactory dysfunction group showed significantly older age than normal controls (57.8 ± 11.9 vs. 47.1 ±12.2; $P<.001$).
**Conclusion:** Our deep learning-based volumetry of olfactory bulb showed excellent performance. It can help to obtain insight about correlation between various diseases and olfactory bulb volume.

**Keywords:** Olfactory nerve, deep learning, segmentation, volumetry, cognition, diabetes, hyposmia, anosmia

### Diagnostic yield of MRI in patients with non-specific or normal head and neck examination findings at a UK teaching hospital

*Authors:* *S. Karthik, B. Owen, C. Vincent, R. Clarkson, S.-J. Choi, S. Vaidyanathan; Leeds Teaching Hospitals NHS Trust, Radiology, Leeds, United Kingdom*

**Purpose/Objectives:** Magnetic resonance imaging (MRI) is commonly performed for patients with suspected or confirmed head and neck cancer. Anecdotally, in our practice, MRI studies of the head and neck have a low yield without associated positive clinical examination findings identified by specialist clinical review.
We aimed to review urgent and fast-track (suspected cancer) MRI requests for patients which detailed non-specific or normal clinical examinations and correlate these with imaging findings and clinical outcomes.
**Methods and materials:** MRI's performed from 01/04/2021 to 31/03/2022 were retrospectively analysed with the use of the radiology information system and electronic patient records. Data was recorded and analysed adhering to our local information governance policies.
**Result:** 610 studies were performed on behalf of 21 specialist referrers from our institution and identified for analysis. 85 studies (26 urgent and 59 fast track) met inclusion criteria (15% of all urgent, and 17% of all fast track). 525 of 610 scans were excluded due to one or more of the following reasons: a routine or planned request, staging scans, known malignancy follow-up, and positive clinical findings (e.g., a mass or vocal cord palsy).
Two broad groups of patients were identified, patients reporting symptoms but with no positive clinical examination findings (50 (59%)) and patients with indeterminate examination findings (35 (41%)). A large proportion of these patients were referred with pain, followed by asymmetry or prominence of tonsillar tissue. 16 of 85 (19%) had an equivocal imaging finding. No malignancy or other concerning conditions was identified within either group based on tissue sampling and/or clinical follow-up.
**Conclusion:** There is a background of increasing demand for imaging services at our institution with a year-on-year increase of between 7-10% across modalities which places a strain on resources. For symptomatic patients with normal or non-specific clinical examination findings, MRI has not identified a single case of cancer in 85 patients over a 12-month period. This is consistent with published findings in the literature.[Ref01]
Based on these results, we are altering our local protocolling, downgrading fast-track patients without positive clinical examinations to urgent and performing a more limited 4-sequence study without contrast.

References:
[Ref01] Goodfellow M, Lim C, Tustin H, Mentias Y, Cocks H. , (2022), The diagnostic yield of head and neck imaging in symptomatic patients with a normal clinical examination. , Head & Neck, Journal of the sciences and specialties of the head and neck., 2564-2570., 44(11), 10.1002/hed.27168, 2023-03-10

### Differentiation between pleomorphic adenoma and mucoepidermoid carcinoma based on changes in the greater palatine foramen

*Authors:* *H.-K. Choi[1], G.-D. Jo[2], J.-H. Kang[2], J.-E. Kim[1], W.-J. Yi[1], M.-S. Heo[1], S.-S. Lee[1], K.-H. Huh[1]; [1]Seoul National University, Oral and Maxillofacial Radiology, Seoul, Republic of Korea (South Korea), [2]Seoul National University Dental Hospital, Oral and Maxillofacial Radiology, Seoul, Republic of Korea (South Korea)*

**Purpose/Objectives:** Mucoepidermoid carcinoma (MEC) is a common malignant tumor that occurs in the palate, but many cases show benign characteristics on images, making it difficult to differentiate from pleomorphic adenoma (PA), which is the most common benign tumor in the palate. The objective of this study was to investigate the feasibility of distinguishing between MEC and PA in the palate by analyzing changes in the greater palatine foramen (GPF) on computed tomography (CT) scans.
**Methods and materials:** Patients who took CT scan for palatal mass evaluation and were histopathologically diagnosed with PA (N=53) or MEC (N=25) in Seoul National University Dental Hospital between January 2001 and January 2023 were selected. CT scans were evaluated retrospectively for various imaging features, which included the size of the tumors, the effects on the adjacent bone, the obliteration of the fat at the entrance of the GPF, and the presence of expansion of the GPF.

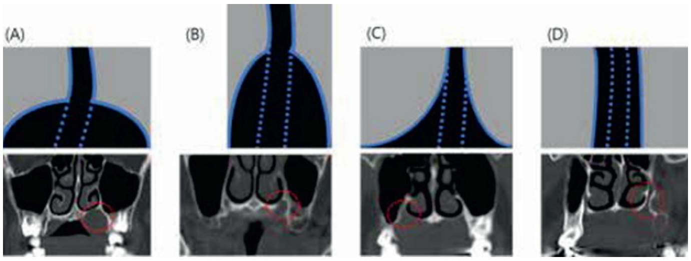

Figure 1. Expansion patterns of greater palatine foramen. (A) Inverted-bowl type. (B) Bell type. (C) Prickle type. (D) Parallel-widening type.

Finally, the expansion pattern, classified as inverted-bowl, bell, prickle, or parallel-widening type, was also evaluated. The effects of each imaging parameter on the differentiation between MEC and PA were compared and analyzed with Spearman's correlation analysis, chi-squared test, Fisher's exact test and logistic regression analysis.
**Result:** Regardless of the tumor size, the majority of MEC demonstrated either obliteration of the fat at the entrance of the GPF or an enlargement of the GPF, while PA exhibited an increasing tendency to obliterate the fat at the entrance of the GPF or to expand the foramina as the tumor size increased ($P<0.05$). The obliteration of the fat at the entrance of the GPF and the destructive change in the adjacent bone were significantly higher in MEC than in PA. Regarding the expansion pattern of the GPF, inverted-bowl type was found only in PA, and prickle and parallel-widening type were more likely to be found in MEC ($P<0.05$). Logistic regression analysis demonstrated that the prickle and parallel-widening type showed the higher predictive power for the differentiation between MEC and PA than destructive change in the adjacent bone.
**Conclusion:** The expansion pattern of the GPF can be a crucial imaging feature on CT scans in distinguishing between MEC and PA.

**Keywords:** mucoepidermoid carcinoma, pleomorphic adenoma

### Double mesiodens: Evaluation by panoramic radiography and Cone Beam Computed Tomography (CBCT) in two cases

*Authors: E. Palizzolo[1], F. Bencivinni[2], A. Lo Casto[1], G. La Tona[1], M. De Angelis[1], A. Inzerillo[1]; [1]Sezione Scienze Radiologiche, BIND, Università degli studi di Palermo, Palermo, Italy, [2]UOS Radiologia Odontoiatrica e Maxillo-Facciale, Università degli Studi di Palermo AOUP P. Giaccone, Palermo, Italy*

**Purpose/Objectives:** Mesiodens is the most common supernumerary tooth with a 0.15-55% prevalence. Double mesiodens is rarer (10-19% of patients with mesiodens), and only few cases of inverted double mesiodens are reported. Mesiodens is usually asymptomatic and included, incidentally discovered on a radiological exam, but sometimes can erupt, be responsible of alterations on surrounding teeth, as eruptive delay, ectopic eruption, malposition, cystic or dysplastic evolution, inflammation. Two cases of double mesiodens, one of them with double inversion, studied by panoramic radiography and CBCT, are described.
**Methods and materials:** Two children, a 5-year-old girl and an 11-year-old boy, were initially submitted to panoramic radiography for orthodontic evaluation. A CBCT was then acquired in both patients.
**Result:** On panoramic radiograph two supernumerary teeth were appreciable between 11 and 21 above, and 51 and 61 below, in a 5-year-old girl. (Fig1)

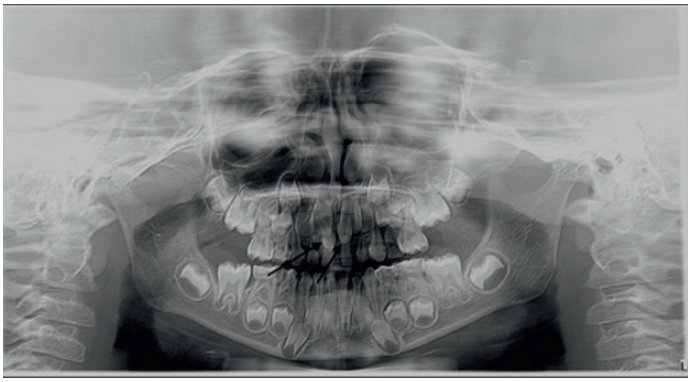

Double mesiodens in a 5-year-old girl. Panoramic radiograph. Two supernumerary teeth are appreciable between 11 and 21 above, and 51 and 61 below.

A CBCT, aimed to confirm the presence of the supernumerary teeth and better assess their relation with contiguous teeth and surrounding anatomic structures, was then performed. The two mesiodens were vertical, palatal oriented and had a conoid shape. (Fig2)

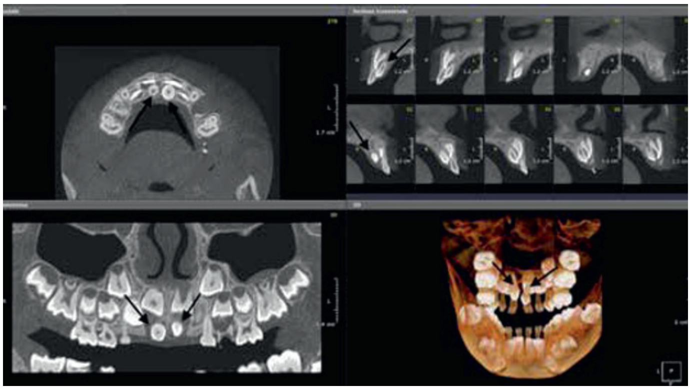

Double mesiodens in a 5-year-old girl. CBCT. The two mesiodens were vertical, palatal oriented, had a conoid shape and were contiguous with the nasopalatine canal.

Case 2
On panoramic radiograph two supernumerary teeth were visible between the roots of 11 and 21, whose roots were displaced, in a 11-year-old boy. (Fig3)

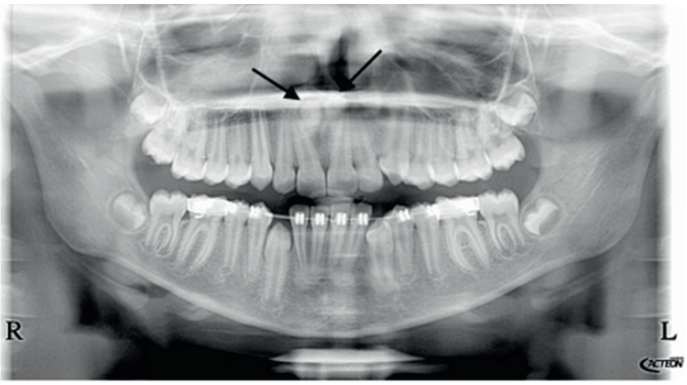

Double inverted mesiodens in a 10 year old boy. Panoramic radiograph. Two supernumerary teeth, partially overimposed to the hard palate, are seen between 11 and 21, whose roots are obliquely angled and distally displaced.

A CBCT, aimed to confirm the presence of the supernumerary teeth and better assess their exact position and relation with surrounding anatomic structures, was then performed. The two mesiodens were both inverted and had a conoid shape. They were located on the palatal median line posterior to the upper permanent incisors, and in contact with the nasopalatin canal. The crown of the mesiodens on the left side laid just below the nasal floor. (Fig4)

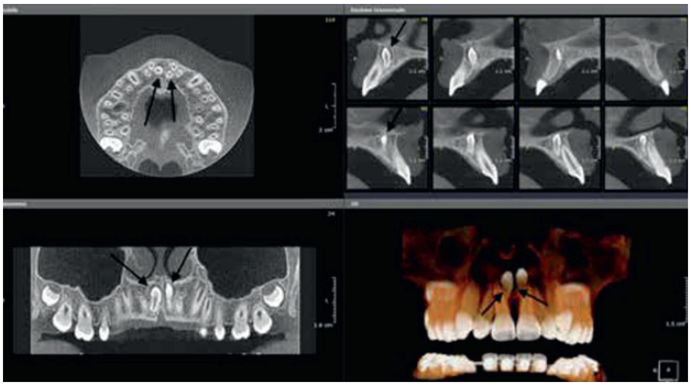

Double inverted mesiodens in a 10 year old boy. CBCT. The conoid shape of the two inverted mesiodens is appreciable. Moreover the palatal position, the relation with the nasopalatin canal and the contiguity of the 21 crown with the above nasal floor are demonstrated.

**Conclusion:** The careful evaluation of panoramic radiography is useful for an early diagnosis of excess dental anomaly, allowing to prevent possible complications affecting the deciduous and, most of all, permanent teeth and plan the most effective treatment, but CBCT is essential as, thanks to 3D reformations, it's able to better assess the exact localization and anatomical relationships of the supernumerary teeth with the contiguous anatomical structures for a correct therapeutic approach.

### Evaluating MRI estimated tumour thickness as a pre-treatment biomarker for survival in patients with locally advanced oral cavity cancer treated with surgery and post operative radiotherapy

*Authors: K. Sambasivan[1], C. Dudau[2,3], K. Anthony[1,4], P. Imran[1], M. Reis Ferreira[1,4], M. Lei[1], T. Guerrero Urbano[1,4], S. Connor[2,3]; [1]Guy's and St Thomas' NHS Foundation Trust, Clinical Oncology, London, United Kingdom, 2Guy's and St Thomas' NHS Foundation Trust, Department of Radiology, London, United Kingdom, 3Kings College Hospital, Department of Neuroradiology, London, United Kingdom, 4Kings College London, London, United Kingdom*

**Purpose/Objectives:** [1]Depth of invasion (DOI) is a well known prognostic factor for oral cavity squamous cell cancer (OCSCC)1. However, it can only be accurately measured after surgical resection, once patients have undergone a major operation. We sought to assess 1) if tumour thickness (TT) as measured on a diagnostic MRI could be used as a surrogate marker for DOI and 2) if TT carries prognostic significance for patients with stage III-IV OCSCC.
**Methods and materials:** This is a retrospective analysis of 77 patients with OCSCC treated with surgery and post-operative radiotherapy (PORT) between 2010 and 2021, who also had a diagnostic MRI. Two head and neck radiologists reviewed the MRI images to independently measure the TT, and information regarding the DOI was retrieved from pathology reports. Inter-observer agreement regarding estimated TT was assessed by the intra class correlation coefficient (ICC) and the relationship between TT and DOI by the Pearson's correlation coefficient. Survival analyses were conducted to evaluate the prognostic significance of the DOI and estimated TT. Statistical analysis was performed using IBM SPSS 28 software.

**Result:** Overall 75 patients were included for analysis; 2 were excluded due to missing DOI information. The median DOI for the population was 18mm (interquartile range IQR 11-25mm) and the median TT 16.5mm (12-22.5mm). The correlation coefficient between TT and DOI was 0.639, p<0.001 (see Figure 1). The ICC between the two sets of estimated TT measurements was 0.8797. Overall survival (OS) was 84% at 1 year and 42% at 5 years. Survival analyses according to DOI and TT are showed in Table 1. Stratification of DOI and TT at 5 and 10mm had no significant effect on either OS or DFS. Stratification at 20mm resulted in a significant effect on OS and DFS for TT, but not for DOI. Figure 2 shows Kaplan Meier survival curves for patients with TT ≤ 20mm vs > 20mm.
**Conclusion:** Our results show good consistency between 2 head and neck radiologists in TT estimation. The positive correlation between TT and DOI suggests that estimated TT could be used as an indication of the actual DOI. It would be useful to assess concordance between a larger cohort of radiologists to see if TT can be reliably measured on MRI imaging, if it is to be used as a pre-treatment surrogate of DOI, which would support its routine use in oral cavity cancer staging. Our results did not demonstrate prognostic value for either DOI or TT cut-offs specified in the current (AJCC 8) staging system for OCSCC, at 5 or 10mm. This could be due to most patients having DOI/TT values >10mm; the lower quartiles were 11 and 12mm A higher cut-off at of 20mm resulted in TT having a significant effect on DFS and OS. This should be validated prospectively in a larger study sample.

**Keywords:** Oral cavity cancer, depth of invasion, tumour thickness

References:
[1] Ebrahimi A, (2014), Primary tumor staging for oral cancer and a proposed modification incorporating depth of invasion: an international multicenter retrospective study., JAMA Otolaryngol Head Neck Surg

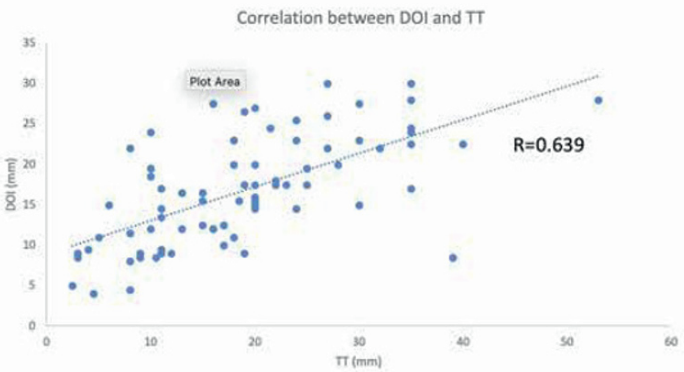

Figure 1. Correlation between actual DOI and estimated TT.

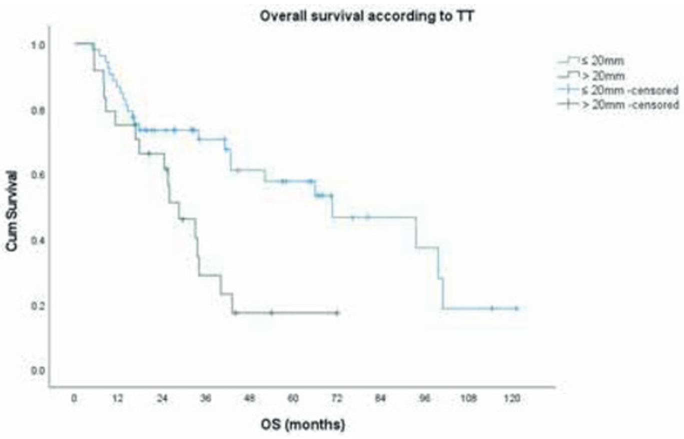

Figure 2. Kaplan Meier curve of overall survival according to tumour thickness (TT)

| | Overall survival (p value) | Disease free survival (p value) |
|---|---|---|
| DOI ≤ 5mm vs > 5mm | 0.793 | 0.843 |
| DOI ≤ 10mm vs > 10mm | 0.933 | 0.272 |
| DOI ≤ 20mm vs > 20mm | 0.966 | 0.183 |
| TT ≤ 5mm vs > 5mm | 0.358 | 0.222 |
| TT ≤ 10mm vs > 10mm | 0.893 | 0.932 |
| TT ≤ 20mm vs > 20mm | 0.002 | 0.005 |

Table 1. Results of log rank tests to assess effects of DOI and TT on survival at different thresholds. Significant results are in bold.

### Hydrops MRI imaging in correlation with clinical diagnosis: EcochG and Glycerol test in Menière disease

*Authors: D. Grosser[1], E. Avallone[2], K. Döring[3], F. Götz[3], A. Warnecke[2], H. Lanfermann[3], T. Lenarz[2], A. Giesemann[3]; [1]Klinikum Hannover, Department of Radiology, Hannover, Germany, [2]Medical School Hanover, Department of Otorhinolaryngology, Hannover, Germany, [3]Medical School Hanover, Diagnostic and interventional Neuroradiology, Hannover, Germany*

**Purpose/Objectives:** The classic symptom triad of acute recurrent attacks of rotary vertigo with nausea and vomiting, hearing loss with sensation of aural fulness and tinnitus is suspicious for the diagnosis of Menière disease. The exact etiology of Menière disease is still unknown, histologically an endolymphatic hydrops is strongly correlated with the disease. The clinical diagnosis is made using the AAO-HNS guidelines (consensus statement of the American Academy of Otolaryngology, Head and Neck Surgery 1995). In vivo tests such as transtympanic electrocochleography (EcochG) and glycerol testing can support the clinical diagnosis, however, as has been supported by numerous studies, the results are heterogeneous and often not consensual. The aim of the present study is to evaluate the sensitivity of MRI imaging compared to the classical EcochG and Gylcerol test.
**Methods and materials:** Fifty-eight patients with Possible, Probable, or Definite Mèniere's Disease underwent diagnostic MRI inner ear imaging, Glycerol and EcochG testing.
The mean age of the cohort is 54.2 years (range 17-82 years). Two patients were affected bilaterally. Twenty-one patients reported right-sided symptoms; 37 left. Imaging of the Hydrops was performed 4h post i.v. double dose contrast application using a 3Tesla MRI Skyra (Siemens, Medical Systems), 64 channel head coil. The subtraction method with positive perilymph and positive endolymph space images were used to detect inner ear Hydrops of the cochlea and vestibule. Two experienced neuroradiologists evaluated the imaging data.
**Result:** In 30 patients with Definite Mèniere´s Disease, Glycerol test was positive in 14 (47%), EchoG test was positive in 14 (47%) and gadolinium MRI imaging diagnosed cochlea hydrops in 83% and vestibular hydrops in 57%). Only 23% of those definitely affected by Mèniere had positive findings in all three diagnostic procedures.
In 8 subjects with symptom-based Probable Mèniere´s Disease, only three (38%) had a positive EcochG test, seven (88%) had a positive glycerol test and six showed hydrops on MR imaging (75%). In 20 with Possible Mèniere´s Disease, Glycerol test was positive in 11 (55%), EchoG test was positive in 8 (48%) and gadolinium MRI imaging diagnosed chochlear hydrops in 83% and vestibular hydrops in 30%.
**Conclusion:** In our study, cochlear hydrops was detected proportionally more often than vestibular hydrops and in comparable studies possibly due to the subtraction method, revealing more subtle cochlea hydrops.
The present study confirms the heterogeneity of clinical and diagnostic test results in the diagnosis of Mèniere's disease and emphasizes that imaging and classical examination methods opportune to otolaryngology, such as EcochG and glycerol tests, are often not consensual.

**Keywords:** Mèniere´s Disease, MRI inner ear imaging, electrocochleography, hydrops

### Imaging features of metastatic lymph nodes and extranodal spread in HNSCC

*Authors: G. Ficarra[1], M. Verda[2], L. Carmisciano[3]; [1]San Paolo Hospital, Savona, Italy, [2]Santa Corona Hospital, Pietra Ligure, Italy, [3]DISSAL, University of Genoa, Genoa, Italy*

**Purpose/Objectives:** Lymph node evaluation is a crucial step in head and neck squamous cell carcinomastaging, influencing treatment choice and prognosis, and representingone of the hardest challenges for radiologists. The aim of our study is to evaluatethe prognostic value of CT and MR imaging features commonly related to metastatic nodesand extranodal extention.
**Methods and materials:** We retrospectively evaluated all imaging examinations obtained for surgical assessment before neck dissection for head and neck squamous cell carcinoma at our institutionin a 6-year interval. Two radiologists indipendently reviewed all scans, noting imaging features and an overall impression about the presence of metastases and ENE expressed as a binary variable (yes/no). Features considered for N-staging were size, central necrosis, non homogeneus contrast enhancement, grouped nodes and diffusivity restriction (for MRI only); Features considered for ENE were peripheral necrosis, unshaped margins and confluent nodes. Imaging findings were compared with histopathological results.
The study population has been splitted in a training set and a validation set; a multivariable logistic model was trained. Model performance was then tested on the validation dataset and compared to the overall assessment.
**Result:** One-hundred-twenty-eight patients were evaluated for metastatic involvement, and 51 for ENE. The overall impression and each evaluated parameter except the presence of grouped nodes resulted significantly associated with the presence ofnodal metastasis, whereasoverall impression and unsharp margins resulted significantly associated withthe presence of ENE. The combination of size, necrosis, nonhomogeneous c.e. and the presence of grouped nodes performed no better than the best single predictor considered alone.
Regarding the evaluation of N-staging,overall impression resulted in higher accuracy compared to the multivariate model. Regarding the assessment of ENE,overall impression showed a lower accuracy compared to the multivariate model but a higher Youden Index.
**Conclusion:** Our results showed no significant difference among imaging modalities (CT and MR) in nodal metastases assessment. MRI performed slightly better in terms of ENE detection, particularly resulting in a slightly higher sensitivity; anyway, due to clinical implications of ENE assessment in HNSCC authors recommend to prefer specificity over sensitivity, in order not to exclude patients that might benefit from surgical treatment. Imaging based staging resulted to be reliable and radiologists should especially consider central necrosis and non-homogeneous contrast-enhancement to assess N stage disease. ENE assessment should be based on the presence of unsharpened margins, rather than on peripheral necrosis.

**Keywords:** HNSCC, metastatic nodes, extra-nodal extension

### Improvement in predictive value of nodal staging system by incorporating radiological advanced extranodal extension to stage N3 in nasopharyngeal carcinoma

*Authors: Q. Y. H. Ai[1], A. D. King[2], Y. M. Tsang[2], K. F. Hung[3], L. M. Wong[2]; [1]The Hong Kong Polytechnic University, Department of Health Technology and Informatics, Kowloon, Hong Kong, [2]The Chinese University of Hong Kong, Department of Imaging and Interventional Radiology, SHATIN, Hong Kong, [3]The University of Hong Kong, Division of Applied Oral Sciences & Community Dental Care, Pokfulam, Hong Kong*

**Purpose/Objectives:** Nodal metastases are the main determinant of outcome in patients with nasopharyngeal carcinoma (NPC) and radiological advanced extranodal extension (ENE) shows the potential in adding predictive value to the current nodal staging system. Recent studies suggest that another radiological feature of ENE, nodal matting, also predicts outcomes but there is a paucity of studies to validate these findings. This ENE study aims to investigate the predictive values of modified nodal systems which incorporate radiological advanced ENE or nodal matting ENE into the current stage N3 of NPC.
**Methods and materials:** A retrospective review of pre-treatment head and neck staging MRIs from 750 consecutive patients with newly diagnosed and non-disseminated NPC was performed. Advanced ENE was defined as infiltration into adjacent muscle/skin/salivary and nodal matting as two or more adjacent lymph nodes with ENE that merge with partial or complete loss of the intervening plane. Three modifications to the criteria of the current N3 staging system ($N_{8th}$) were evaluated which incorporated advanced ENE ($N_{advanced\ ENE}$), nodal matting ENE ($N_{matting\ ENE}$), and advanced ENE/matting ENE ($N_{advanced\ ENE/matting\ ENE}$). The predictive values of these three modifications were compared to ($N_{8th}$) for disease-free survival (DFS) and distant metastases-free survival (DMFS) using concordance statistics (c-indexes). A p-value of < 0.05 indicated statistical significance.
**Result:** The 5-year DFS and DMFS were 74.2% and 83.1% respectively. C-indexes of the $N_{8th}$ and three modified nodal staging systems are shown in Table 1.

Table 1. C-index of the current nodal staging system ($N_{8th}$) and three modified nodal staging systems for 5-year DFS and DMFS

| | DFS | | DMFS | |
|---|---|---|---|---|
| | C-index (95%CI) | P-value | C-index (95%CI) | P-value |
| 5-year | | | | |
| $N_{8th}$ | 0.69 (0.67–0.70) | Ref. | 0.71 (0.69 – 0.74) | Ref. |
| $N_{advanced\ ENE}$ | 0.72 (0.71- 0.73) | **<0.01** | 0.75 (0.73 -0.77) | **< 0.01** |
| $N_{matting\ ENE}$ | 0.68 (0.66 – 0.69) | 0.42 | 0.70 (0.69 – 0.72) | 0.60 |
| $N_{advanced\ ENE/matting\ ENE}$ | 0.68 (0.67 – 0.69) | 0.50 | 0.71 (0.69 -0.72) | 0.72 |

Compared with $N_{8th}$, only $N_{advanced\ ENE}$ showed statistically higher c-indexes for 5-year DFS and DMFS (0.69 vs 0.72, 0.71 vs. 0.75, respectively, p <0.01).

**Conclusion:** Results suggest that for radiological ENE, advanced ENE is the strongest predictor of outcome and should be considered as a criterion for stage N3 disease in NPC.

**Keywords:** Nasopharyngeal carcinoma, extranodal extension, MRI, predictive value

### Model of vestibular implant stimulation in rhesus labyrinth estimates current spread to facial and cochlear nerves

*Authors: A. Hedjoudje[1,2,3], E. Vesper[3], B. Morris[3], C. Della Santina[3]; [1]Sion hospital, Sion, Switzerland, [2]University of Geneva, Geneva, Switzerland, [3]Johns Hopkins Hospital, Baltimore, United States of America*

**Purpose/Objectives:** To study the spread of current in a vestibular implant aimed at restoring balance sense after the loss of inner ear hair cell function, a detailed model of the inner ear geometry was developed. The previous work had modeled the spread of current from spherical electrodes to the divisions of the vestibular nerve innervating the five vestibular end organs, but current spread to the facial and cochlear nerves was not considered, and it used outdated software. An updated model was developed that includes the activation of the facial nerve and all six branches of the vestibulocochlear nerve. The model was optimized to work with more readily available software and implement parallel computing to speed up computational time.
**Methods and materials:** The model was developed using a normal rhesus macaque temporal bone scanned using micro-MRI and reconstructed to generate model geometry with physiological tissue conductivities, including anisotropic nerve conductance. The model's geometry was refined using Solidworks to place spherical electrodes in the semicircular canals. The COMSOL Multiphysics software was used for meshing and finite element modeling (FEM) of electrical currents, assuming quasistatic conditions. The extracellular potential field at model neuron nodes of Ranvier was calculated and convolved in time with a single biphasic cathodic pulse. These were input to stochastic, nonlinear dynamic spiking models with randomized parameters of 2,415 vestibular afferent axons, 1,000 cochlear afferent axons, and 500 facial afferent axons to determine action potential initiation. The pulse amplitude threshold above which prosthetic stimulation elicited an action potential at the medial end of the model axon within 2 ms of pulse delivery was determined for each axon. Each axon was given a unique location on the neuroepithelium and trajectory through its nerve. Parallel computing was used to speed computational time on multiprocessor computers.
**Result:** For a given electrode pair and stimulus pulse amplitude, relative axon recruitment was calculated for each nerve branch. Simulation of spike generation and propagation of 3,915 neurons for one electrode combination took ~11 minutes to compute on a 3.5-GHz 16-core 64-bit PC running Windows 10. This is a significant improvement from the previous model, which took 400 minutes per electrode to simulate 2,415 axons.
**Conclusion:** The model can be extended with different electrode designs and to human anatomy to facilitate the development of electrode designs, surgical techniques, and stimulus paradigms for the use of vestibular implants in the treatment of human patients with severe vestibular hypofunction.

**Keywords:** Modelling, vestibular system, vestibular implant

### MRI in diagnosis of metastatic Lymph Node in patient with Oral Squamous Cell Carcinoma

*Authors: P. Buscemi[1], A. Monteleone[1], M. Nichelini[2], F. Pandolfo[1], R. Cannella[1], F. Bencivinni[3], A. Lo Casto[1]; [1]Sezione di scienze radiologiche, BIND, Università degli studi di Palermo, AOUP P. Giaccone, Palermo, Italy, [2]Chirurgia plastica e ricostruttiva, Dipartimento di discipline chirurgiche, oncologiche e stomatologiche, Università degli studi di Palermo, AOUP P. Giaccone, Palermo, Italy, [3]UOS Radiologia Odontoiatrica e Maxillo-Facciale, AOUP P. Giaccone, Palermo, Italy*

**Purpose/Objectives:** The occurrence of metastatic lymph nodes in lateral cervical stations has a significant impact on lifespan of patients with oral squamous cell carcinoma (OSCC), influencing treatment and in particular the surgical approach to a possible lymphadenectomy. MRI is often used to detect the presence of metastatic lymph nodes. The three main morphological criteria currently used to evaluate metastatic lymph nodes in MRI are size, colliquation and extra nodal diffusion. Growing in size is the most common characteristic of a metastatic lymph node. Also, metastatic lymph nodes often exhibit a rounded shape and a short axial diameter (SAD) ≥10 mm is generally considered the cutoff for malignancy on axial images. Colliquation and extra nodal diffusion are less used criteria for this purpose. The aim of this study was to evaluate the diagnostic performance of MRI in diagnosis of metastatic lymph nodes in patients with OSCC.
**Methods and materials:** Head and neck MRI exams of 67 consecutive patients (26 females and 41 males, 29-88 years, average 65), studied and treated between 2017 and 2022, with OSCC and malignant lateral cervical lymph nodes at histological examination were retrospectively analyzed. T1, with and without fat saturation and i.v. contrast medium, and T2 and STIR sequences on the axial plane with a maximum 4 mm slice thickness were used to evaluate lymph node morphology. The SAD of lymph nodes of stations IA, IB, IIA, IIB, III, IV, V and VI, bilaterally, were considered using the ≥10 mm cutoff for malignancy, except for the jugulodigastric node where a ≥11 mm cutoff was adopted. Colliquation and extra nodal diffusion were evaluated too.
**Result:** MRI criteria gave 35 (52.2%) true positive cases, and 32 (47.8%) false negative cases. Colliquation occurred in 27/67 patients with a 14 mm median SAD of the colliquated lymph node. Only in 1/27 patients colliquation was a useful finding in considering malignant a lymph node with SAD <10 mm.
**Conclusion:** Data obtained in our cohort of patients with OSCC demonstrate that currently used MRI criteria to diagnose metastatic lymph nodes give a very high number of false negative. Cutoff SAD of lymph nodes in the evaluation of head and neck OSCC should be reduced in order to improve the diagnostic performance of MRI in detection of metastatic nodes.

### MRI-derived radiomic features for discriminating between cancerous and non-cancerous palatine tonsils

*Authors: K. F. Hung[1,2], R. Zhang[3], A. D. King[3], L. M. Wong[3], Y. Y. Leung[2], Q. Y. H. Ai[3,4]; [1]Faculty of Dentistry, The University of Hong Kong, Applied Oral Sciences and Community Dental Care, Hong Kong, Hong Kong, [2]Faculty of Dentistry, The University of Hong Kong, Oral and Maxillofacial Surgery, Hong Kong, Hong Kong, [3]The Chinese University of Hong Kong, Imaging and Interventional Radiology, Hong Kong, Hong Kong, [4]The Hong Kong Polytechnic University, Health Technology and Informatics, Hong Kong, Hong Kong*

**Purpose/Objectives:** This preliminary study aimed to evaluate whether radiomic features extracted from the non-contrast-enhanced T2-weighted fat-suppressed (T2-fs) magnetic resonance imaging (MRI) images could differentiate between cancerous and non-cancerous palatine tonsils.
**Methods and materials:** MRIs of 25 patients without head-neck cancer (age ranged 40-62) and 39 patients with histologically confirmed palatine tonsillar cancer (age ranged 43-84) were retrospectively collected. 50 non-cancerous and 41 histologically confirmed cancerous palatine tonsils from the two cohorts were manually segmented on the axial T2-fs images by two experienced researchers, separately, using ITK-SNAP. All images were applied N4ITK bias field correction algorithm to remove the nonuniform intensity and the "$\mu \pm 3\alpha$" algorithm to exclude image intensity outliers. 105 quantitative features in "First-order", "Shape", and "Texture" categories were automatically extracted from two separate sets of segmentations using SimpleITK and PyRadiomics. Features with intra-class coefficient (ICC) >0.85 were selected. The z-score normalization was applied to all features. Feature selection was performed on the training set. The *p*-values for each feature between cancerous and non-cancerous tonsils in the training set were calculated and only the features with a $p < 0.05$ were reserved. The LASSO (least absolute shrinkage and selection operator) algorithm was performed to identify the potential features using the minimum criteria to tune the regularization parameter ($\lambda$) via 10-fold cross validation. The radiomics signature was built using the selected features with nonzero coefficients. A logistic regression classifier was performed to construct radiomic models. The 3-fold cross validation was used to assess the model performance. The performance metrics were AUC, accuracy, sensitivity, and specificity. The optimal thresholds for identifying tonsillar cancer on MRI were obtained by maximizing the Youden index.
**Result:** The radiomic model in each of the three folds shows the AUCs of 0.75-0.98 for identifying tonsillar cancer on T2-fs images (Figures 1&2). The overall AUC was 0.86 across three folds. By using the optimal threshold, sensitivity of 82.9%, specificity of 86.0%, and accuracy of 84.6% were achieved (Table 1).
**Conclusion:** The preliminary results suggested that radiomics analysis of non-contrast-enhanced MRI with the non-transformed features has the potential to discriminate palatine tonsillar cancer from normal palatine tonsils. The preliminary results may serve as the foundation for further improvement and optimization of the radiomics model for such application. The stability of feature selection and the generalizability of the model should be further tested in external data sets.

**Keywords:** palatine tonsillar cancer, non-contrast-enhanced MRI, radiomics analysis

### Non-attendance at neck ultrasound appointments: Can we predict who isn't going to attend?

*Authors: T. Watson[1], P. Moyle[1], B. Hodgkinson[2], K. A. Eley[3]; [1]Cambridge University Hospitals NHS Foundation Trust, Radiology, Cambridge, United Kingdom, [2]Cambridge University Hospitals NHS Foundation Trust, Central Informatics, Information Management Systems and Analytics, Cambridge, United Kingdom, [3]University of Cambridge, Radiology, Cambridge, United Kingdom*

**Purpose/Objectives:** Missed appointments waste public healthcare resources and lead to delays in patient care. The purpose of this study was to investigate the factors affecting attendance rates at head & neck outpatient ultrasound appointments. Due to anecdotal experience, we hypothesised that: (1) patients having to travel further would be less likely to attend; (2) attendance rates for procedural ultrasound appointments (e.g. biopsies) would be lower than non-procedural appointments; and (3) younger patients would be less likely to attend.
**Methods and materials:** Retrospective data was gathered from the electronic patient record system (Epic) of all attended and missed outpatient ultrasound appointments (for all anatomical regions) in 2021 at our hospital. For each appointment, travel distance (straight line distance from patient's address to the hospital), age, sex, and type of investigation were gathered. Patients were excluded if there were no travel distance data available. After exclusions there were 975 missed appointments and 30936 attended appointments.
**Result:** Overall non-attendance rates were 3.1% across all ultrasound appointments, and 2.7% for neck ultrasound ($p>0.05$). The mean age for non-attended appointments was lower (38.6 vs 48.6 years, $p < 0.001$), with male patients representing a greater proportion of missed appointments (45.7% vs. 38.0%, $p < 0.00001$).
A significant difference was identified in non-attendance rates for procedural ultrasound appointments compared to non-procedural ultrasound appointments across all subspecialties (1.8% vs. 3.1%, $p < 0.01$). However, this difference was not seen for neck ultrasound appointments (2.1% vs 2.8%).
No significant difference was identified in mean travel distance between attended and missed appointments (19.7 vs. 20.2 km), nor between distance travelled and non-attendance rate.
**Conclusion:**
(1) Travel distance does not affect non-attendance rate for ultrasound appointments.
(2) Patients are significantly more likely to attend procedural appointments than non-procedural appointments, although this trend becomes non-significant when restricted to neck ultrasound, which may be due to smaller sample sizes. Such appointments may be perceived as more important by the patient (e.g. a biopsy for suspected cancer).
(3) Patient age is a factor for non-attendance.
Further work is required to understand the reasons underlying this behaviour to guide patient education and improve efficacy of ultrasound appointments.

**Keywords:** ultrasound; attendance; DNA.

### Parathyroid adenoma: Radiologic features and correlationstudy with 99mTc-sestamibi SPECT findings

*Author: H. Kang; Seoul Veterans Hospital, Department of Radiology, Seoul, Republic of Korea (South Korea)*

**Purpose/Objectives:** Our study was intended to describe the typical radiologic features of parathyroid adenoma and correlation study with $^{99m}$Tc-sestamibi SPECT findings.
**Methods and materials:** A review of a surgical database and electronic medical records from 2015 to 2022 identified the cases of 17 patients who underwent preoperative cervical sonography for primary hyperparathyroidism with subsequent resection of pathologically proven parathyroid adenoma. Two radiologists retrospectively evaluated the preoperative ultrasound images and assessed for morphologic characteristics, size, location, and color Doppler vascularity. $^{99m}$Tc-sestamibi SPECT findings, surgical and pathologic reports were reviewed.
**Result:** Most of the parathyroid adenomas (15/17, 86%) were deep or inferolateral to the adjacent thyroid. An echogenic border separating the adenoma from the overlying thyroid was identified in all cases. 14 cases were solid (82%), one case was solid and cystic, and two cases were cystic (11%) in sonographic morphology. Color Doppler examination of 14 patients showed feeding vessels with internal color flow to the solid components in 7 patients (50%). All patients underwent preoperative $^{99m}$Tc-sestamibi SPECT, and the adenoma was definitively localized in 9 patients (52%) and equivocally localized in 6 patients (34%). One case of ectopic mediastinal parathyroid adenoma was identified by $^{99m}$Tc-sestamibi SPECT and subsequently confirmed by chest computed tomography.
**Conclusion:** Awareness of typical radiologic features (location, morphologic characteristics, color Doppler findings) may aid radiologists in preoperative localization of parathyroid adenoma. And $^{99m}$Tc-sestamibi SPECT is also helpful to the diagnosis of this tumor.

**Keywords:** parathyroid adenoma, ultrasonography, $^{99m}$Tc-sestamibi SPECT

### Prediction of hemifacial spasm re-appearing phenomenon after microvascular decompression surgery in patients with hemifacial spasm using dynamic susceptibility contrast perfusion MRI

*Authors: C.-W. Ryu, H. c. Kho, S. Park, G.-H. Jahng; Kyung Hee University Hospital at Gangdong, Radiology, Seoul, Republic of Korea (South Korea)*

**Purpose/Objectives:** A hemifacial spasm (HFS) re-appearing phenomenon after surgery (HFSrapas) is one of the unpredictable courses when HFS ceases immediately after microvascular decompression (MVD) and then reappears within about four days. The resolution of HFSrapas requires a substantial time. In this study, we investigated the occurrence of HFSrapas with dynamic susceptibility contrast (DSC) perfusion MRI to be able to predict it using both a receiver operating characteristic (ROC) curve and machine learning analyses.
**Methods and materials:** The 60 patients were enrolled in this study and were divided into 2 groups: 32 patients (53.3%) with HFSrapas (Group A) and 28 patients (46.7%) whose symptoms of spasm completely disappeared and never recurred (Group B). With the use of DSC perfusion MRI taken preoperatively, the following DSC indices were measured and evaluated to predict reappearing symptom using both ROC and machine learning methods: relative cerebral blood volume (rCBV), relative cerebral blood flow (rCBF), relative mean transit time (rMTT), leakage, extraction fraction (EF).
**Result:** The voxel-based analysis showed that both rCBF and rCBV reduced in the symptom reappearance group, but both rMTT and EF increased in the symptom reappearance group in the most of brain areas. The ROI-based analysis showed that in the reappearing group compared to the no-reappearing group, the rCBF value significantly decreased in most of the selected brain areas. The largest AUC value to predict the differentiation between reappearing and no-reappearing symptom groups was obtained with the NB machine learning model by combining the three different EF values at MTG, PC, and brainstem and age (AUC=0.845).
**Conclusion:** The machine learning analysis showed that the combination of the EF parameter with the demographical parameters such as age or sex can predict HFS patients with reappearing from no-reappearing symptom after MVD surgery. Thus, DSC perfusion MRI is useful tool to predicate HFS recurrence before intra-operation, and help neurosurgeon predict possible problems during MVD surgery.

**Keywords:** MRI; Dynamic susceptibility contrast perfusion; Hemifacial spasm; Microvascular decompression Surgery; Reappearing symptom; Prediction; Extraction fraction

### Segmentation of the intraparotid facial nerve: Usefulness of neurographic MRI sequences in the management of parotid tumours. Preliminary experience

*Authors: S. Medrano-Martorell, Y. Rodríguez-Álvarez, A. Morales-Rosa, V. Richart-Sierra, F. J. Cuesta-González, J. M. Costa-González, N. Bargalló-Alabart; Hospital Clínic de Barcelona, Neuroradiology, Barcelona, Spain*

**Purpose/Objectives:** Surgery for parotid gland tumors has a relatively high rate of complications. Among these, facial nerve palsy is the one that has the greatest impact on quality of life.
The intraglandular course of the facial nerve with the classic structural imaging techniques of CT and MRI, that are commonly used in clinical practice, can only be approximated through indirect methods. In the last decade, several high-resolution MR sequences have been developed to allow direct visualization of the intraparotid nerve.
The objectives of this presentation will be:
- To present some high-resolution MR sequences that allow visualization of the intraglandular course of the facial nerve.
- To show examples of the first cases in which we have obtained the segmentation of the intraglandular facial nerve through neurographic sequences as well as the radio-surgical correlation.

**Methods and materials:**
1. Acquisition of high-resolution DESS (double-echo-steady-state) and T1w TSE-VFA (T1-weighted 3D turbo spin-echo with variable flip angles) sequences in patients with parotid tumors.
2. Three-dimensional segmentation of the intraparotid facial nerve.
3. Surgical photographs.
4. Radio-surgical consensus/correlation.

**Result:** As an example, images of segmentation work of a few individuals with parotid tumors will be presented.
These images will be correlated with the surgical findings.
**Conclusion:** With the introduction of these neurographic techniques, it is intended to improve the surgical planning of facial nerve dissection in parotid tumor surgery with the ultimate goal of optimizing the preservation of the facial nerve in the surgical outcome of parotid gland surgery, as well as minimizing the secondary effects. On the other hand, it is intended to gain experience in obtaining and segmenting this type of images and to take a first step towards the future validation of our segmentation method.

**Keywords:** Segmentation. Facial nerve. Parotid tumours

### Tonsillar Actinomycosis that Mimicking Tonsillar Neoplasm

*Authors: R. Karaca[1], S. Karaca[2]; [1]Darende State Hospital, Radiology, Malatya, Turkey, [2]SK Private Clinic, Otolaryngology, Malatya, Turkey*

**Purpose/Objectives:** Actinomycosis is a chronic suppurative infection caused by a group of bacteria called Actinomycetaceae. Cervicofacial actinomycosis is the most common form of the disease and results from bacteria becoming invasive through a mucosal lesion. We report a case of tonsillar actinomycosis that cause massive, asymmetric enlargement of tonsil and mimic the neoplasia. The purpose of this case presentation is to consider tonsillar actinomycosis as a rare entity in cases with asymmetric tonsillar hypertrophy with suspected malignancy.
**Methods and materials:** A 54 year-old woman presented with a 4-days history of sore throat and fever. The patient had a history of recurrent tonsillitis for 2 years and consumed smoke for 40 years. On examination, the right tonsil was grade 4 hypertrophic and the left tonsil was grade 1 hypertrophic. There was a growing irregular surface of in the right tonsil. There were nicotine stains on the teeth. Ear, laryngeal and nasopharyngeal examinations were normal. Routine blood tests were normal. Biopsy of the right tonsil was done under general anesthesia and sent for histopathological examination. Histopathological examination showed an inflammatory lesion. Magnetic resonance imaging (MRI) planned because of clinical suspicion of malignancy. On MRI examination, there was a right oropharyngeal relatively homogeneous enhanced mass centered on the right tonsillar fossa.

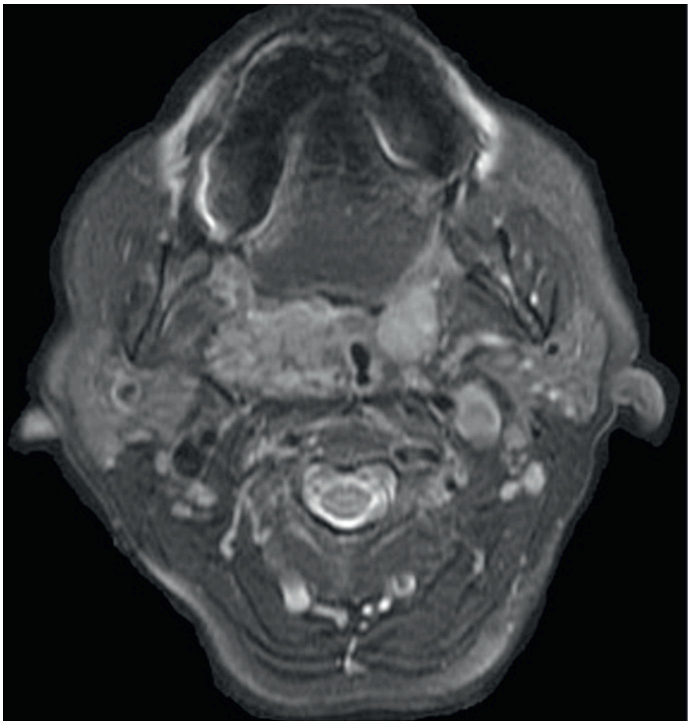

Axial T2-weighted MR image shows a hyperintense mass in right tonsillar fossa. Mass extends the retropharyngeal space and abuts from the right longus colli muscle. The left tonsil is minimal hypertrophic.

The mass had small necrotic areas or cystic portions.
**Result:** The patient subsequently underwent bilateral tonsillectomy. Histopathological examination revealed multiple foci of Actinomycetaceae in the right tonsil and chronic lymphoid hyperplasia in the left tonsil. Postoperatively, the patient was received antibiotherapy. The patient had no complaints at the postoperative first- and second-week follow-up and her physical examination was normal. Three months after surgery, the patient recovered clinically and symptomatically.
**Conclusion:** Actinomycosis can mimic any oropharyngeal malignancies clinically and radiologically. However, it is unusual for actinomycosis to cause unilateral, massive tonsillar hypertrophy that mimic neoplasia.Tonsillar actinomycosis is a rare entity that should be kept in mind when clinically and radiologically asymmetric tonsillar hypertrophy is encountered.

**Keywords:** Actinomycosis, Cervicofacial actinomycosis, Tonsillar neoplasm

### Underlying Bone Change in Oral Squamous Cell Carcinoma: Implications for Tumor Aggressiveness and Prognosis

*Authors: G.-D. Jo[1], H.-K. Choi[1], J.-H. Kang[1], J.-E. Kim[2], W.-J. Yi[2], M.-S. Heo[2], S.-S. Lee[2], K.-H. Huh[2]; [1]Seoul National University Dental Hospital, Seoul, Republic of Korea (South Korea), [2]Seoul National University, Seoul, Republic of Korea (South Korea)*

**Purpose/Objectives:** In cases where oral squamous cell carcinoma (OSCC) invades the jaw bone, radiologists often observe abnormal attenuation on computed tomography (CT) and pathological signal intensity (SI) on magnetic resonance (MR) images of the affected underlying bone marrow. This phenomenon is commonly referred to as underlying bone change. The purpose of this study was to determine whether underlying bone change is associated with the progression and prognosis of OSCC, and to investigate the relationship between underlying bone change and the type of bone invasion.
**Methods and materials:** We enrolled 93 consecutive patients diagnosed with OSCC who underwent mandibulectomy at Seoul National University Dental Hospital between 2009 and 2015. Patients with recurrent OSCC and those who received preoperative radiotherapy or chemotherapy were excluded. CT and MR images and electronic medical records were reviewed, and correlations between underlying bone change, type of bone invasion, clinicopathological factors, and overall survival were assessed. The Pearson $\chi 2$ test or the Fisher exact test was performed to evaluate the correlation among underlying bone change, type of invasion, and clinicopathological factors. Cumulative survival rates were calculated using the Kaplan-Meier method, and differences between the curves were analyzed using the log-rank test. A P value less than .05 was considered statistically significant. All statistical analyses were performed using IBM SPSS Statistics Version 21 (IBM Co, Armonk, NY).
**Result:** Underlying bone sclerosis on CT images was observed in 69 (74%) of the 93 cases, and pathological SI on MR images was observed in 74 (80%) of the 93 cases of OSCC. Underlying bone change was correlated with the extent of the tumor ($P$ = .001) and recurrence ($P$ = .017). However, no significant difference was observed in overall survival between patients presenting with underlying bone change and those without it.
Regarding bone invasion, medullary bone invasion was observed in 61 (66%) of the 93 cases. The type of bone invasion was strongly associated with the extent of the tumor ($P$ < .001) and TNM stage ($P$ < .001). Patients with medullary invasion demonstrated significantly poorer overall survival compared to those without it ($P$ = .003).
The underlying bone change on CT and MR images was positively associated with aggressive bone invasion type ($P$ = .001 and = .016, respectively).
**Conclusion:** Underlying bone change on CT and MR images may provide additional information for determining the aggressiveness of bone invasion in OSCC and assist clinicians in making more informed decisions regarding treatment strategies. Further research is needed to explore the potential role of underlying bone change as a prognostic indicator in OSCC.

**Keywords:** Underlying bone change, Oral squamous cell carcinoma, Bone invasion

### Unilateral vocal cord paralysis – From brain to cord

*Authors:* *N. Zorlu, B. Bryl, N. Bhatt; Luton and Dunstabke UHB, Luton, United Kingdom*

**Purpose/Objectives:** UVCP is a common presentation which can be the first presentation of severe pathology. The complicated anatomy and broad range of causes can lead to significant anxiety for the reporting radiologist. Our learning objectives are as follows;
- Normal anatomy of the larynx, vagal nerve and recurrent laryngeal nerves
- Imaging findings in unilateral vocal cord paralysis (UVCP).
- Mimics of UVCP.
- Demonstration of a logical approach to UVCP, to be used by reporters.
- Case series illustrating different examples of UVCP.

**Methods and materials:** We will demonstrate the normal anatomy of the larynx, vagal nerves and recurrent laryngeal nerves and provide typical imaging findings in UVCP. We will provide imaging mimics and pitfalls to avoid while reporting. The importance of the anatomy will be highlighted by a variety of illustrated cases. A logical approach to UVCP will be provided to improve confidence in identifying both the paralysis, and also the cause of the paralysis

**Result:** Cases include:
- Skull base metastases from breast carcinoma
- Oesophageal carcinoma
- Mediastinal mass secondary to metastatic breast carcinoma
- Thoracic aortic aneurysm
- Mediastinal invasion of small cell lung cancer
- Thyroid goiter
- Thyroid carcinoma
- Mediastinal adenopathy due to sarcoidosis

**Conclusion:** With an understanding of the anatomy and the simple logical approach provided, UVCP does not need to be a source of anxiety for reporting radiologists. The case series will increase understanding and increase confidence in reporting the broad range of pathologies which can present with UVCP.

**Keywords:** Unilateral vocal cord paralysis, vocal cord paralysis, laryngeal nerve

### Utility of high resolution magnetic resonance imaging for non-arteritic anterior ischemic optic neuropathy; a preliminary study

*Authors:* *J. E. Park, R. G. Yoon; University of Eulji College of Medicine, Nowon Eulji Medical Center, Department of Radiology, Seoul, Republic of Korea (South Korea)*

**Purpose/Objectives:** Magnetic Resonance Imaging (MRI) is commonly used to differentiate patients with suspected Non-Arteritic Anterior Ischemic Optic Neuropathy (NAION) or inflammatory optic neuritis. The purpose of this study was to demonstrate the utility of high-resolution MRI sequences in detecting NAION.

**Methods and materials:** We conducted a retrospective review of the medical records and MRIs of eight patients suspected of having NAION, who underwent orbit protocol MRI. The orbit MRI protocol included axial T1-weighted imaging (T1WI), high-resolution 3D T2-weighted imaging (HR-T2WI), high-resolution 3D contrast-enhanced T1-weighted imaging (HR-CE-T1WI), and high-resolution 3D T2-fluid attenuated inversion recovery imaging (HR-T2-FLAIR). We evaluated the presence of optic disc swelling, contrast enhancement at the optic disc, and other abnormal findings on these sequences to rule out inflammatory optic neuritis. The final clinical diagnosis of NAION was established by a 12-week follow-up ophthalmologic exam and laboratory testing.

**Results:** Of the eight patients, seven were diagnosed with NAION. Two of these patients demonstrated optic disc swelling on T2WI and focal contrast enhancement on HR-CE-T1WI or HR-T2-FLAIR imaging. Five patients showed focal contrast enhancement on HR-CE-T1WI or HR-T2-FLAIR imaging without evidence of optic disc swelling on HR-T2WI; four of these patients showed contrast enhancement only on HR-T2-FLAIR (Fig 1). One patient, confirmed to have optic perineuritis with papilledema, showed optic disc swelling with contrast enhancement at optic disc, and also perineural enhancement along the intraorbital segment of optic nerve.

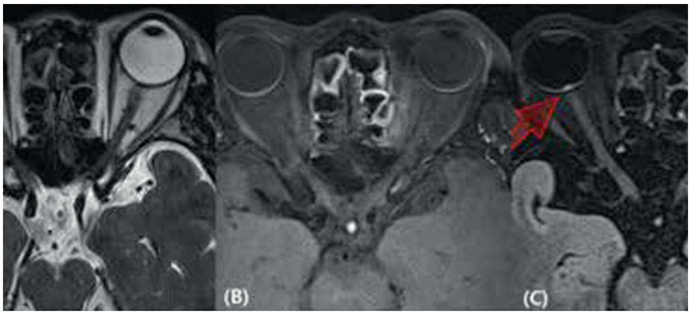

A 70-year-old male patient with NAION. (A) Axial T2-weighted SPACE imaging demonstrating no definite optic disc swelling at the right optic disc. (B) Axial CE-T1WI showing no definite enhancement at the right optic disc. (C) Axial T2-FLAIR imaging demonstrating definite focal contrast enhancement at right optic disc.

**Conclusion:** This study demonstrated the utility of high resolution MRI sequences for detecting NAION. High-resolution T2-FLAIR imaging is particularly useful in detecting NAION, especially when combined with other high-resolution MRI sequences. It can provide additional insights into the pathophysiology of NAION, which may aid in making an appropriate diagnosis and follow-ups. Further studies with larger sample sizes and diffusion-weighted image are warranted to validate the results of this study.

**Keywords:** Orbit; Non-Arteritic Anterior Ischemic Optic Neuropathy; Magnetic resonance imaging

References:
[NEJM] Valérie Biousse, and Nancy J. Newman , (2015), Ischemic Optic Neuropathies, Massachusetts Medical Society, The New England Journal of Medicine
[NO] Ore-ofe O. Adesinaa, J. Scott McNallye, Karen L. Salzman, Bradley J. Katz, Judith E. A. Warner, Molly McFadden, and Kathleen B. Digre, (2018), Diffusion-Weighted Imaging and Post-contrast Enhancement in Differentiating Optic Neuritis and Non-arteritic Anterior Optic Neuropathy, Taylor & Francis, NEURO-OPHTHALMOLOGY

### Educational Posters

### A case of an accessory ocular muscle: A rare cause of restrictive strabismus

*Authors: C. Chaves[1], P. Barradas[1], F. Rodrigues[1], C. Paiva[2], C. Nunes[1]; [1]Centro Hospitalar e Universitário de Coimbra, Serviço de Imagem Médica, Neurorradiologia, Coimbra, Portugal, [2]Centro Hospitalar e Universitário de Coimbra, Hospital Pediátrico, Serviço de Oftalmologia, Coimbra, Portugal*

**Learning objectives:** To review the aetiology and clinic findings of an accessory ocular muscle; to illustrate the CT and MRI appearances of an accessory ocular muscle and the differential diagnoses; to understand the available options of management.
**Background:** The orbits contain muscular, nervous and vascular structures which contribute for the normal function of the globe, and can be affected by various pathologies. One of the rarest anomalies that can disturb this balance is the existence of an accessory extraocular muscle that interferes with the normal movement of the ocular globe.
**Imaging findings and procedure details:** A 1-year-old child was brought to the emergency department with 1-month history of failure to rise the right eye. The neurologic exam detected limited supraversion and enophtalmia of the right eye. An orbital nonenhanced CT scan, and an orbital MRI study revealed an anomalous intraconal structure, with a thickness of approximately 2.3 mm, originating at the orbital apex, traveling within the muscle cone, in a position inferolateral to the optic nerve, superolateral to the inferior rectus, medial to the lateral rectus, and inserting on the inferomedial posterior surface of the right globe. The CT attenuation of this structure was similar to the other extra-ocular muscles. The MRI study showed a similar signal emission of the other extra-ocular muscles, namely with isosignal intensity on T1 and T2-weighted images, and homogeneous enhancement. This structure was surrounded by intraconal fat and did not establish contact with any of the typical extra-ocular muscles, which had a normal morphology, implantation and size. No other intraconal or extraconal abnormalities were visualised in the studies.
**Conclusion:** This abnormal structure found by CT and MRI was the cause of the elevation deficit and presumed to be an accessory extraocular muscle. Despite their extreme rarity, this structure should be considered in the differential diagnosis of pathologies causing restrictive strabismus. Orbital imaging studies should be done to explore the origin of the disease.

**Keywords:** Strabismus, Orbit, Extraocular muscle

### A practical approach to the most common orbital masses in adults

*Authors: B. Zoltai[1], L. Ujváry[2], H. Sükösd[1]; [1]Semmelweis University, Medical Imaging Centre, Budapest, Hungary, [2]Semmelweis University, Department of Ophthalmology, Budapest, Hungary*

**Learning objectives:** Readers will learn to identify and interpret the most commonly encountered findings in the orbit. Their typical clinical presentation, typical imaging appearance and potential pitfalls will be discussed. This presentation focuses on some of the common orbital vascular lesions, benign tumors and malignancies.
**Background:** A wide variety of processes can produce mass lesions in and around the orbit such as neoplastic, vascular, inflammatory, developmental. The imaging modalities used to delineate orbital soft tissue lesions, distinguish malignant from benign lesions, evaluate the location and extent of orbital tumors. On the other hand, computed tomography is the preferred mode for imaging of the orbital bones, sinuses, and osseous lesions and can detect calcifications within lesions.
Compartment-based approach and knowledge of the characteristic features on magnetic resonance and computed tomography imaging can lead to distinguishing among orbital lesions.
**Imaging findings and procedure details:** Key imaging features that guide radiological diagnosis are discussed and illustrated.
**Conclusion:** Orbital masses in adults are not common but encompass a wide spectrum of benign and malignant entities that range from developmental anomalies to primary and secondary orbital malignancies and metastatic disease. Imaging is mandatory for narrowing the diagnostic considerations and determining the most appropriate management strategy. Improving basic knowledge, determining typical imaging appearance through a systematic approach may greatly improve detection and therapeutic results as well.

**Keywords:** orbital masses, adults

### A retrospective review to assess the effectiveness of our investigative pathway for the presentation of spontaneous CSF rhinorrhoea

*Authors: O. O'Brien, D. Ellul, I. V. McCrea; NHS Greater Glasgow & Clyde: Queen Elizabeth University Hospital, Otolaryngology department, Glasgow, United Kingdom*

**Learning objectives:**
1. Analyse current imaging practice in our centre.
2. Optimise imaging pathway for the investigation of spontaneous CSF leak.

**Background:**Untreated/undiagnosed CSF leaks can lead to potentially serious complications such as meningitis, cerebritis and cerebral abscess. Clinical assessment, beta-2 transferrin testing of nasal fluid, HRCT sinus, MRI and cisternography all play an important role in the investigation of CSF leak detection.[1][2]

We conducted a retrospective analysis of all patients imaged for an initial presumed diagnosis of spontaneous CSF rhinorrhoea in NHS Greater Glasgow & Clyde over a two-year period to assess the effectiveness of CT and MRI for detecting the site of a leak.

**Imaging findings and procedure details:** Using a CRIS search for 'CSF rhinorrhoea, CSF leak' from January 2019 to December 2020 we identified 297 patients who had imaging for CSF leak. From this group we identified a cohort of 22 patients who underwent imaging after presenting with a clinical picture in keeping with spontaneous CSF rhinorrhoea. 73% (16) were female. The average age was 51.5 years (range 27 – 77).

Of the 22 investigated, 18 (82%) had HRCT sinuses, 11 (50%) had MRI imaging and 10 (45%) had both CT and MRI. 9/11 (82%) who underwent MRI had a dedicated sinus protocol performed (two patients only had MRI brain protocol), only one of which included a thin slice high resolution heavily T2-weighted sequence.

Beta-transferrin test was performed on rhinorrhoea fluid in 11 patients, 6 of which were positive and 5 negative.

Of 6 positive cases, a defect was identified in 4 patients on HRCT, 3 of whom underwent surgery to repair the defect, the intraoperative defect site corresponding to the radiologically identified site in all cases. A further one patient with positive beta-transferrin and negative HRCT sinus underwent CT cisternography which demonstrated a defect site, the same defect site was subsequentially identified intraoperatively and repaired. One patient was lost to follow up.

All patients with negative beta-transferrin test (5 patients) or no beta-transferrin test performed (11 patients), had negative imaging.

**Conclusion:** HRCT sinuses is effective for identifying the site of CSF leak in patients who have a proven CSF leak on beta-transferrin rhinorrhoea fluid analysis.

In our cohort, MRI had a limited role to play in identifying the site of CSF leak. This may be due to suboptimal imaging protocol with lack of high resolution T2 weighted imaging.

We propose a standardised imaging protocol/algorithm to optimise the investigation of spontaneous CSF rhinorrhoea.

**Keywords:** 'Rhinorrhea', 'HRCT sinus', 'MRI sinus, 'CT cisternography'

References:
[1] Vemuri NV, Karanam LS, Manchikanti V, Dandamudi S, Puvvada SK, Vemuri VK., (2017), Imaging review of cerebrospinal fluid leaks, Wolters Kluwer, Indian Journal of Radiology and Imaging, 441-6, 4, 2023-03-31
[2] Bhatia D, Murthy N., (2020), Comparative retrospective study of HRCT, CT Cisternography, and MRI in evaluation of CSF leak, East African Scholars Publisher, EAS Journal of Radiology and Imaging Technology, 7-12, 4, https://www.easpublisher.com/media/features_articles/EASJRIT_24_7-12_cc.pdf, 2023-03-31

### A Review of Imaging Strategies for Investigating Unilateral Vocal Cord Paralysis

*Author: E. J. Lee; Dongguk University Ilsan Hospital, Radiology, Goyang-si, Gyeonggi-do, Republic of Korea (South Korea)*

**Learning objectives:**
- To review the normal anatomy of vagal and recurrent laryngeal nerves and vocal cord
- To show normal cross-sectional anatomy of the vagal and recurrent laryngeal nerves on a CT scan
- To illustrate the imaging findings of various lesions causing vocal cord paralysis according to a course-based approach

**Background:** Vocal cord paralysis is the total interruption of nerve impulses resulting in no movement of internal laryngeal muscles, and not open or close the true vocal cord properly.

Vocal cord movement muscles are mainly innervated by the recurrent laryngeal nerves from the vagus nerve. Thus, vocal cord paralysis can be caused by pathology in the brainstem or anywhere along the course of the nerves supplying the vocal cords.

Due to the long anatomical course of the vagal and recurrent laryngeal nerves, many disease processes can cause vocal cord paralysis.

**Imaging findings and procedure details:**
1. Introduction and general overview of vocal cord paralysis
2. Anatomic review of the vocal cord and imaging findings of vocal cord paralysis, its mimics, and pitfalls.
3. Anatomic review and CT imaging-based normal cross-sectional anatomy of the vagal and recurrent laryngeal nerves
4. Imaging findings of various lesions causing vocal cord paralysis along the course of the vagal and recurrent laryngeal nerves
   1) intracranial pathologies affecting the brainstem or the skull base
   2) according to the course-based approach, extracranial pathologies are divided into:
      - carotid space
      - visceral space
      - mediastinal space
5. Demonstration of the treatments and their image appearance

**Conclusion:** By understanding and evaluating the entire course of the vagal and recurrent laryngeal nerves, radiologists can avoid missing causative lesions, many of which are more significant and serious to patients than the vocal cord paralysis itself.

### A Snapshot of Pterygopalatine Fossa Anatomy and Tumoral Involvement

*Author: T. Akdag; University of Health Sciences, Diskapi Yildirim Beyazit Training and Research Hospital, Department of Radiology, Ankara, Turkey*

**Learning objectives:**
- To describe the anatomy of the pterygopalatine fossa (PPF).
- To discuss the imaging features of tumours involving the PPF

**Background:** PPF is located between the maxillary bone anteriorly, the pterygoid process posteriorly, orbital apex superiorly and orbital process of the palatine bone medially. Foramen Rotundum and Vidian canals can be used as imaging landmarks to delineate the PPF on imaging (Figure 1).

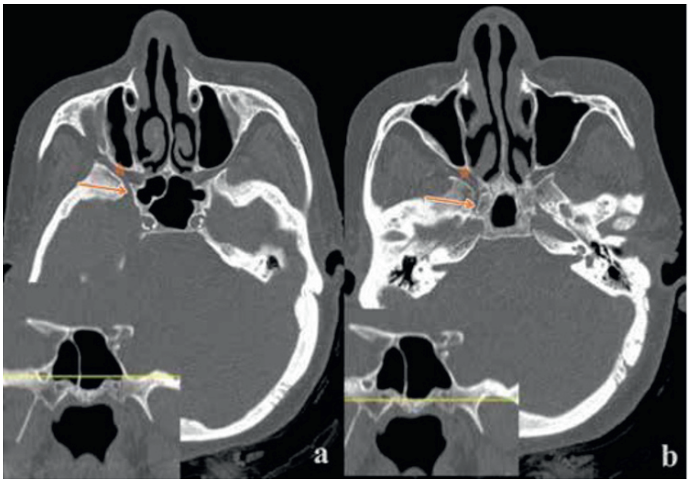

(a) Foramen Rotundum (red arrow) on axial CT image connects the PPF (star) to the middle cranial fossa posteriorly (b) The Vidian canal (red arrow) is seen posterior to the Foramen Rotundum. Inst images showing these structures in a coronal plane (yellow line)
The most superior aspect of the PPF is the inferior orbital fissure, Inferiorly the fossa is contiguous with the greater and lesser palatine foramen. Communication with the nasal cavity is via the sphenopalatine foramen medially. Laterally, the pterygomaxillary fissure connects the PPF to the infratemporal fossa (Figure 2 a,b,c,d).

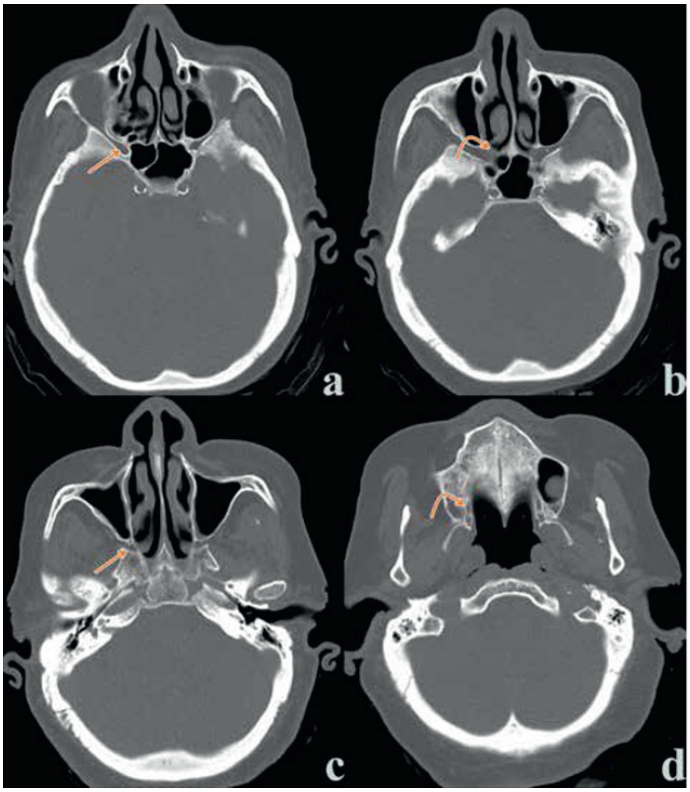

a) Axial CT scan shows the inferior orbital fissure (arrow). (b) The sphenopalatine foramen (arrow) communicates the PPF with the nasal cavity. (c) Pterygomaxillary fissure (arrow) leads PPF to the infratemporal fossa. (d) The greater&lesser palatine canals connect the PPF with the oral cavity at its most inferior aspect

PPF serves as a highway between nearby anatomic structures throughout the foramen and fissure. Due to its strategic location between orbit, middle cranial fossa, nasal cavity, nasopharynx, infratemporal fossa, and oral cavity, the PPF may be affected either by loco-regional tumoral invasion or by perineural tumour spread (PNTS). Recognizing the perineural spread of the tumors is crucial as its involvement is an independent risk factor for the patient survival (1).
CT and magnetic resonance imaging (MRI) are complementary techniques for evaluating the PPF. With a CT scan, the fossa's bony boundaries and any bone invasion is well demonstrated (2). MRI is most reliable method for grading of tumors, to detect PNTS and for monitoring (3).

**Imaging findings and procedure details:** Retrospective evaluation of MRI features of 22 patients with tumors invading the PPF was performed. The study included 15 males and 7 females with ages ranging between 21 and 85 years. All patients were examined on 1.5 Tesla magnets before and after injection of Gadolinium Meglumine. MRI features of various masses invading pterygopalatine fossa were reviewed. Out of 22 PPF masses, the most common tumors were adenoid cystic carcinoma (n:5) (Figure 3) squamous cell carcinoma (n:5) (Figure 4) and nasopharynx carcinoma (n:4) (Figure 5).Other diagnosis included: malignant peripheral nerve sheath tumors (Figure 6), angiofibroma (Figure 7) lymphoma (Figure 8) round small cell tumor (Figure 9) and, hypophysis adenoma (Figure 10).

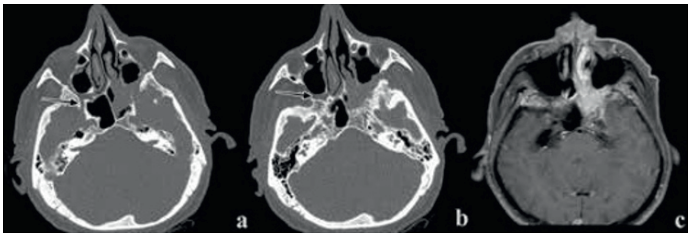

Nonkeratinized SCC of nasopharynx. (a)Normal foramen rotundum on the right and tumoral spread&bone destruction on the left. (b) Normal right sphenopalatine foramen (black arrow), while on the left this level is infiltrated by the tumor. (c) Post-contrast axial image shows an enhancing mass extending to the left PPF, nasal cavity and sphenoid sinus.

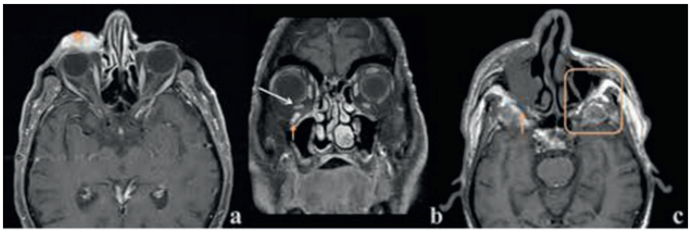

(a)An 85-year-old-male presented with a mass with intense contrast enhancement. (b) On coronal image PNTS of the lesion through the infraorbital nerve (red arrow). (c)In the follow-up, pathologically proven malignant peripheral nerve sheath tumor increased in size and extended into PPF (right). Note normal pterygopalatine fossa on the left.

**Conclusion:** Awareness of the complex anatomy of the PPF and its neuronal communications helps to simplify whilst imaging on the tumoral involvements. For the PPF evaluation, CT and MRI are complimentary techniques and a careful review in terms of detect locoregional or perineural spread is critical.

**Keywords:** pterygopalatine fossa, anatomy, tumours, computed tomography, magnetic resonance imaging

References:
[1] Moreira MCS, Dos Santos AC, Cintra MB. , (2017), Perineural spread of malignant head and neck tumors: review of the literature and analysis of cases treated at a teaching hospital. , Radiol Bras, 323-327, 50(5)
[2] 2. Derinkuyu BE, Boyunaga O, Oztunali C, Alimli AG, Ucar M, (2017), Pterygopalatine Fossa: Not a Mystery!, Can Assoc Radiol J. , 122-130, 68(2)
[3] Tashi S, Purohit BS, Becker M, Mundada P, (2016), The pterygopalatine fossa: imaging anatomy, communications, and pathology revisited. , Insights Imaging, 589-99, 7(4)

### Allergic sialadenitis – Role of the radiologist in diagnosis and management

*Authors: J. Flexen, S. Harvey, L. Feinberg; University College London Hospital, Clinical Radiology, London, United Kingdom*

**Learning objectives:**
- Gain an understanding of this emerging disease process and its presentation features.
- Appreciate difficulties in diagnosis and most common differential diagnoses.
- Become familiar with the role of the radiologist in diagnosis and management options.

**Background:** Allergic sialadenitis (also known as eosinophilic sialadenitis or sialodochitis) is an uncommon cause of recurrent major salivary gland swelling (with a preponderance for the submandibular glands) and is associated with atopy. It is a recently described entity with few cases reported in the literature. Diagnosis centres on a supportive clinical history of atopic disease along with eosinophilia in aspirated mucus plugs and peripheral eosinophilia. Treatment options include medical management such as antihistamines and systemic corticosteroids along with ductal cannulation and irrigation or steroid injection. [1][2]

**Imaging findings and procedure details:** We present a series of cases reviewing normal anatomy of the major salivary glands (focussing on the submandibular glands) and features of allergic sialadenitis on ultrasound and sialography. We demonstrate an intra-oral approach to salivary intervention and management.The poster will include a 'how to' box on how we treat patients at our institution.

**Conclusion:** Although uncommon, allergic sialadenitis should be considered in all patients with recurrent salivary gland swelling. Radiologists can play a pivotal role not only in diagnosis but also in management.

**Keywords:** allergic sialadenitis; salivary glands; eosinophilic sialodochitis: allergy

References:
[1] Baer et al., (2017), Eosinophilic sialodochitis: re-definition of "allergic parotitis" and "sialodochitis fibrinosa", Oral Diseases, 840-848, 23(7)
[2] Carey et al., (2022), Eosinophilic sialodochitis: An emerging atopic condition, Oral diseases, 648-656, 28(3)

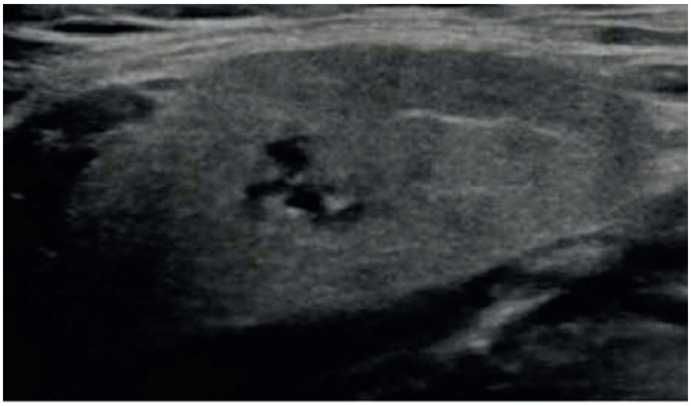

US right submandibular gland

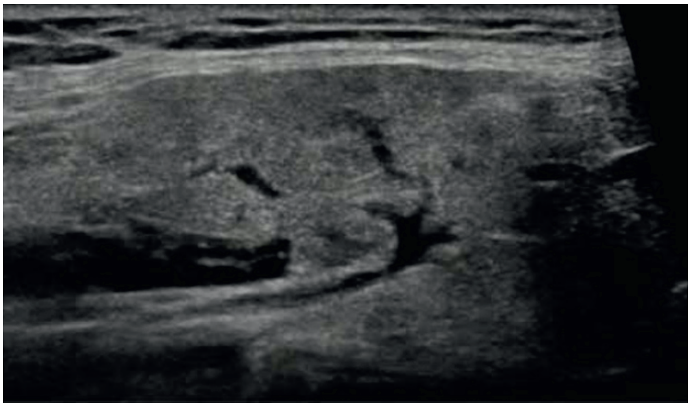

US left submandibular gland

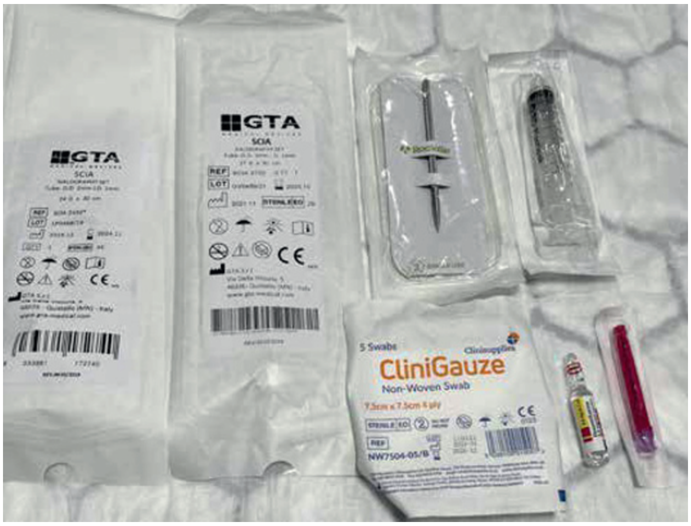

Set-up for major salivary gland washout

### Beyond Squamous Cell Carcinoma: A Review of Laryngeal Lesions and Their Clinical and Histopathological Findings

*Authors: A. Öztürk[1], Y. Pekçevik[1], U. Küçük[2], I. B. Arslan[3], I. Çukurova[3]; [1]Izmir Health Sciences University, Tepecik Training and Research Hospital, Radiology, Izmir, Turkey, [2]Izmir Health Sciences University, Tepecik Training and Research Hospital, Pathology, Izmir, Turkey, [3]Izmir Health Sciences University, Tepecik Training and Research Hospital, Otolaryngology-Head and Neck Surgery, Izmir, Turkey*

**Learning objectives:** Review the diagnostic clinical and imaging findings, as well as the histopathological characteristics of laryngeal lesions other than squamous cell carcinoma (SCC)
Demonstrate key clinical, imaging, and histopathological features of the lesions for differential diagnosis.
**Background:** Laryngeal lesions other than SCC are rare but can have significant clinical implications. These lesions can arise from various sources, such as benign tumors, inflammatory conditions, infectious diseases, and malignancies other than SCC. The clinical presentation of these lesions is diverse and can include hoarseness, cough, stridor, and dyspnea. Imaging modalities such as computed tomography (CT) and magnetic resonance imaging (MRI) help characterize these lesions, guiding biopsy, and evaluating the extent of the disease. Treatment options depend on the type and extent of the lesion and can range from conservative management to surgery and radiation therapy. In this educational exhibit, we provide an overview of laryngeal lesions other than SCC, their clinical and imaging features, and management strategies
**Imaging findings and procedure details:** We reviewed the clinical and imaging features, as well as histopathological findings of patients with laryngeal lesions other than SCC. We demonstrated key imaging, clinical, and histopathological findings that aid in the differential diagnosis.
**Conclusion:** Awareness of these laryngeal lesions and their presentations can aid in early diagnosis and appropriate treatment.

**Keywords:** Laryngeal lesions, CT, MR, PET, imaging, histopathology

### Complications of dental implants: cbct findings a pictoral review

*Authors: M. De Angelis[1], E. Palizzolo[1], A. Inzerillo[1], F. Bencivinni[2], G. La Tona[1], F. Pandolfo[1], R. Di Raimondo[3,4], A. Lo Casto[1]; [1]Sezione scienze radiologiche, BIND, Università degli studi di Palermo, Radiology, Palermo, Italy, [2]U.O. Radiologia odontoiatrica e maxillo-facciale, AOUP, Palermo, Palermo, Italy, [3]Postgraduate of Periodontology, Faculty of Odontology, University Complutense,Madrid,Spain, Madrid, Spain, [4]Postgraduate of Oral Surgery, Periodontology and Implant, University Sur Mississippi, Spain Institution, Madrid, Spain, Madrid, Spain*

**Learning objectives:** Review of dental implants' complications imaged by panoramic radiography (PR), CT or CBCT.
**Background:** The use of dental implants has increased during the last few decades with an increase of related complications. Dental implants are aimed to restore function in partially or completely edentulous patients. In order to assess and maintain dental implants' stability, osseointegration, in which osteoblasts grow and directly integrate with the surface of titanium embedded into the jaw, is necessary. It sometimes might interrupt due to different risk factors (osteoporosis, uncontrolled diabetes, poor oral hygiene, bruxism, radio- and/or chemio-therapy, etc.), leading to complications. Three types may occur: biological complications (mucositis, peri-implantitis, implant loss, infection, failure of bone and soft tissue preservation and injury to surround structures), surgical complications (hemorrhage, injury to inferior alveolar nerve, tissue emphysema and fistule through sinus), technical complications (fractures of implant prostheses and screw loosening). Biological complications can result in serious clinical conditions, including the need to explant the implant fixture or irreversible nerve damage, in fact biological complications, result in greater sequelae compared to other complications. Still, early diagnosis deriving from periodic follow up can contribute to reduce the severity of complications and facilitate appropriate treatment. Radiological examinations including PR is usually the first imaging line, while CBCT is generally suggested when better definition and differential diagnosis in doubtful cases are required.
**Imaging findings and procedure details:** Different types of severe complications of dental implants, such as peri-implantitis, infection, oro-antral fistula, oro-nasal fistula, implant malpositioning and osteomyelitis are presented. PR is the most widely used imaging. This technique is also used for monitoring the bone levels around the implant, following the implant's placement and osseointegration, but it is limited by a 2D vision. CT or CBCT with MPR and dental software reformations is suggested to complete the evaluation of significant parameters, and provide a reliable and comprehensive overview. CT/CBCT is useful to depict subtle bone anatomy of the jaws and surrounding anatomy, especially when precise relationships of nasal cavity, maxillary sinus, mandibular canals, and other critical structures to the site of implant is required.
**Conclusion:** Dental implant complications can lead to regardably dangerous conditions for patients, and it is important for radiologists to be aware of them, either for early detection, or contribute to manage the surgical treatment.

### Congenital dacryocystoceles: A case series from our institution

*Authors: H. Queirós, C. Chaves, P. Barradas, J. Pinto, R. P. Pais; Centro Hospitalar e Universitário de Coimbra, Functional Unit of Neuroradiology, Medical Imaging Department, Coimbra, Portugal*

**Learning objectives:** Our goal is to draw attention to an uncommon condition that may cause respiratory distress in the newborn. Awareness of its imagiological characteristics and relatively benign evolution is important for a proper management and exclusion of possible complications.
**Background:** Congenital dacryocystocele is a rare entity which is thought to result from the obstruction of two sites in the nasolacrimal system: distal at the Hasner valve, and proximal at the Rosenmuller valve [1]. The dacryocystocele may extend intra-nasally forming a nasal cyst located in the inferior meatus. As neonates are obligatory nasal breathers, an obstruction at this level can lead to cyanosis and desaturation, especially during feeding and sleeping [2]. While laryngomalacia and choanal atresia are common causes of neonatal respiratory obstruction, dacryocystocele, meningocele, hemangioma, and nasal glioma should also be considered among differential diagnosis [2], [3]. The diagnosis is clinical, but CT and MRI are also very useful, especially detecting bone changes, and characterising the cyst contents, respectively [1], [2]. Conservative management appears to be sufficient in most cases, whereas surgical intervention is usually indicated in patients with permanent respiratory obstruction [3].
**Imaging findings and procedure details:** A total of 5 patients (3 male, 2 female) were diagnosed with dacryocystocele, between April 2017 and May 2022, in our institution. Age range was 1 to 17 days. Patient data, associated symptoms, imaging findings and treatment options are described in Table 1. Complete resolution was obtained in all cases.

| Age at onset (days) | Sex | Symptoms | Imaging findings | Management |
|---|---|---|---|---|
| 9 | M | None; drainage of mucoid material after direct massage | CT: bilateral dacryocystocele, more prominent at the right inferior nasal meatus | Probing (initially) + Massage |
| 17 | M | None | CT: left dacryocystocele | Probing |
| 3 | M | None | CT and MR: bilateral dacryocystocele | Massage |
| 2 | F | Mild respiratory distress during feeding | CT: bilateral dacryocystocele | Massage + Antibiotics |
| 1 | F | Nasal obstruction with desaturation and cyanosis while feeding | CT: bilateral dacryocystocele, occupying almost the entire inferior nasal meatus (++ left side) | Massage + Hot compresses |

Table 1. Congenital dacryocystocele – patient data, clinical manifestations, imaging findings and management.

**Conclusion:** Despite being rare, congenital dacryocystocele is a possible etiology for neonatal respiratory distress. The diagnosis is clinical, although imaging exams may be requested to confirm it. Although the majority of cases resolves spontaneously or with conservative treatment, surgical intervention may be mandatory in more severe ones.

**Keywords:** Dacryocystocele, Neonatal respiratory obstruction, Imaging findings, Treatment

References:
[1] 1. Cavazza S, Laffi GL, Lodi L, Tassinari G, Dall'Olio D, (2008), Congenital dacryocystocele: diagnosis and treatment, Acta Otorhinolaryngol Ital, 28(6):298-301
[2] 2. Carneiro de Sousa P, Neves M, Duarte D, Azevedo P, (2019), Congenital bilateral dacryocystocele, Eur Ann Otorhinolaryngol Head Neck Dis, 136(1):41-42
[3] 3. Barroso F, Silva R, Mendes C, (2017), Congenital dacryocystocele in infant: A rare cause of eye swelling, Porto Biomed J, 2(1):28-29

### Craniofacial manifestations in achondroplasia: A pictorial review

*Authors: C. Chaves, H. Queiros, R. Sousa, C. Nunes, C. Maia; Centro Hospitalar e Universitário de Coimbra, Serviço de Imagem Médica, Neurorradiologia, Coimbra, Portugal*

**Learning objectives:** To present, summarize and illustrate with cases from our center the multiple and complex craniofacial characteristics of this disorder.
**Background:** Achondroplasia is a congenital genetic disorder and the most common form of short-limb dwarfism. It has a rate of occurrence of 1 in 25,000 births and is inherited as an autosomal dominant trait; however, the majority of cases are sporadic (80%). The phenotypic changes are mainly attributed to the abnormal cartilage growth plate differentiation and the significant decrease in bone development. The diagnosis is made by clinical suspicion and specific features found in imaging, and can be confirmed by genetic testing. Apart from the several recognized physical features of this syndrome, achondroplasia can also affect the central nervous system and lead to some important clinical conditions.
**Imaging findings and procedure details:** The craniofacial features of achondroplasia include macrocephaly, frontal bossing, depressed nasal bridge, maxillary hypoplasia, otolaryngeal system dysfunction, small skull base and foramen magnum stenosis. Various complications can arise, such as hydrocephalus, otitis media, sinusitis, upper-airway obstruction and difficulty in intubation.
**Conclusion:** In our educational exhibit we illustrate numerous imaging findings of the typical achondroplasia craniofacial anomalies in both MRI and CT and we discuss their potential complications.

**Keywords:** Achondroplasia; Craniofacial; Congenital

### Diagnostic Challenges and Follow-Up Imaging in Necrotizing Otitis Externa

*Authors: Y. Pekçevik[1], I. B. Arslan[2], I. Çukurova[2]; [1]Izmir Health Sciences University, Tepecik Training and Research Hospital, Radiology, Izmir, Turkey, [2]Izmir Health Sciences University, Tepecik Training and Research Hospital, Otolaryngology-Head and Neck Surgery, Izmir, Turkey*

**Learning objectives:**
- Review the diagnostic clinical and imaging findings of necrotizing otitis externa (NOE).
- Demonstrate key imaging features of NOE for differential diagnosis.
- Illustrate the spread patterns and complications of NOE during the follow-up period.

**Background:** Necrotizing otitis externa presents clinical and radiological challenges both at the time of diagnosis and during the follow-up period. It is a rare but life-threatening disease with nonspecific symptoms that can mimic other diseases. Accurate diagnosis requires a high index of clinical suspicion and appropriate imaging. Furthermore, the follow-up period is challenging, and early recognition of treatment response and complications is crucial for patient management. Magnetic resonance (MR) imaging of the skull base is superior to other imaging modalities for diagnosing NOE and evaluating intracranial complications related to the disease.
The aim of this educational exhibit is to present case-based clinical and imaging findings of NOE, review differential diagnoses, and illustrate the spread patterns and complications.

**Imaging findings and procedure details:** We reviewed the clinical and imaging findings of patients with NOE at the time of diagnosis and during follow-up. We demonstrated key imaging findings that aid in differential diagnosis, spread patterns of the disease, and associated complications.

**Conclusion:** NEO is a rare but life-threatening condition that requires a high index of clinical suspicion and appropriate imaging for accurate diagnosis. Being familiar with imaging and clinical findings at the time of diagnosis and during follow-up helps in early recognition of NOE and its complications. Magnetic resonance imaging is the imaging modality of choice for diagnosing NOE and evaluating intracranial complications.

**Keywords:** necrotising otitis externa, skull base osteomyelitis, MR imaging

### Epistaxis: Diagnostic Approach to Common and Uncommon Causes

*Authors: E. Marín-Díez[1], J. Viera-Artiles[2], E. Marco de Lucas[2], D. Castanedo Vázquez[2]; [1]Hospital Universitario Marqués de Valdecilla, Radiología, Santandear, Spain, [2]Hospital Universitario Marqués de Valdecilla, Santandear, Spain*

**Learning objectives:** The purpose of this educational exhibit is:
1. Review the anatomical considerations in the ontogenesis of epistaxis.
2. Provide an image-based review of both common and uncommon causes of epistaxis.

**Background:** We have reviewed the relevant literature covering nasal anatomy and its vascularization in rhinological imaging. The common causes of epistaxis are highlighted and the diagnostic approach is explained.

**Imaging findings and procedure details:**
Anatomy:
Angiograms and illustrations.
Correlation between CT/MRI scans and vascular anatomy.
ECA branches:
Internal maxillary artery --> Sphenopalatine and Greater palatine arteries.
Facial artery --> Superior labial artery.
ICA branches:
Ophthalmic artery --> Anterior and Posterior ethmoidal arteries.
Vascular plexus:
Kiesselbach plexus: anterior/inferior nasal septum.
Anterior and posterior ethmoidal arteries (Ophthalmic artery).
Sphenopalatine artery (Internal maxillary artery).
Greater palatine artery (Internal maxillary artery).
Superior labial artery (Facial artery).
Woodruff plexus:
posterior/inferior/lateral nasal septum.
Sphenopalatine artery (Internal maxillary artery).
Pharyngeal arteries.

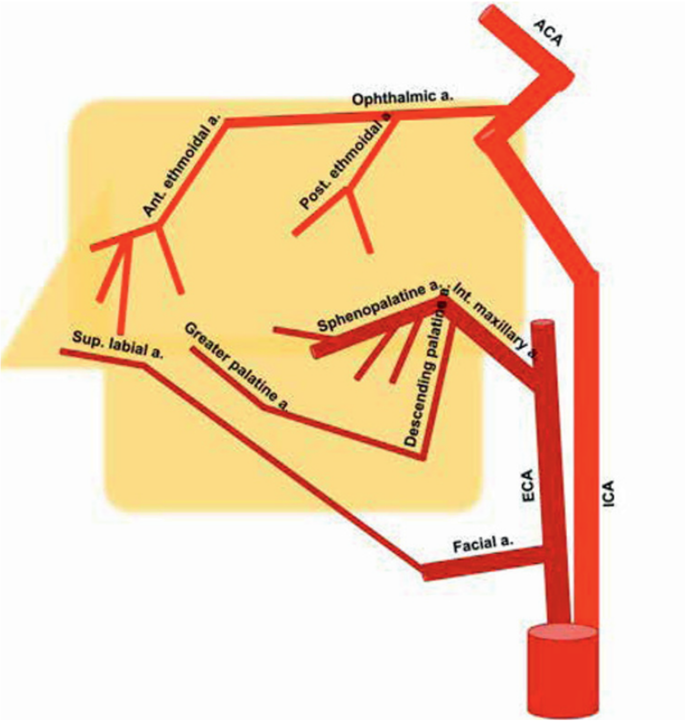

Nasal arteries. A: Internal maxillary artery (yellow arrow) and sphenopalatine artery (blue arrow). B: Ophthalmic artery (yellow arrow), anterior (blue arrow) and posterior (red arrow) ethmoids arteries. C: Facial artery (yellow arrow) and superior labial artery (blue arrow). D: Descending palatine artery (yellow arrow) and greater palatine artery

Case-Based Review of Epistaxis:
TRAUMA
Septal perforation.
Foreign body.
Facial trauma.
Barotrauma.
Cocaine abuse.

VASCULAR ABNORMALITIES AND VASCULAR MALFORMATIONS
Hereditary hemorrhagic telangiectasia.
Granulomatosis with polyangiitis.
Capillary hemangioma.
Intracranial aneurysms.

NEOPLASTIC
Juvenile angiofibroma.
Nasopharyngeal carcinoma.
Paranasal sinus tumors.
Esthesioneuroblastoma.
Nasal melanoma.
Hematolymphoid neoplasms: Lymphoma and plasma cell neoplasms (multiple myeloma and plasmacytoma).
Others.

Summary: algorithm for diagnosis.

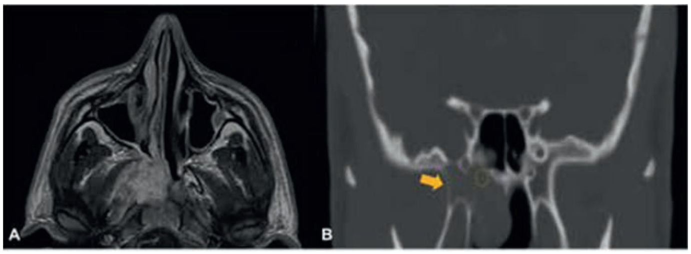

**Juvenile angiofibroma (part 1).** 26-year-old man with juvenile angiofibroma and epistaxis. A-B: There is an homogenously enhancing mass arising just posterior to he sphenopalatine foramen (asteriks in A), extending into posterior nasal cavity. Lytic destruction of the right pterygoid plates (thick arrow in B) and the right vidian canal (circle in B).

**Conclusion:** Arterial anatomy of the nasal passages is important to understanding and identifying causes of secondary epistaxis. The differential diagnosis of causes of epistaxis is vast and encompasses a wide range of etiologic factors, ranging from benign causes, such as digital trauma and foreign bodies, to life-altering entities, including systemic hematologic disorders, locally destructive tumors, and sinonasal malignancies. Patient age and comorbidities are important considerations when assessing underlying cause of epistaxis.

**Keywords:** Epistaxis; Sinonasal Anatomy, Vascularization

### Giant Orbital Rhabdomyosarcoma: Two pediatric case reports

*Authors:* *S. Reigada, C. Chaves, H. Queirós, R. Pais, B. Gomes; Centro Hospitalar e Universitário de Coimbra, Área Funcional de Neurorradiologia - Serviço de Imagem Médica, Coimbra, Portugal*

**Learning objectives:** The purpose of this work is to review the radiological aspects of orbital Rhabdomyosarcoma as well to present two atypical cases of patients that have been managed at our institute.
**Background:** Rhabdomyosarcoma (RMS) is a highly malignant tumor and is the most common soft-tissue sarcoma of the head and neck in childhood with 10% of all cases occurring in the orbit.
Orbital RMS is the most common orbital malignancy of childhood, with approximately 4 or 5 new cases per year per million children younger under 20 years of age. Survival has changed drastically over the years, from 30% in the 1960's to 90% presently, with the advent of new diagnostic and therapeutic modalities.
Radiological imaging and histopathological examinations are crucial for differential diagnosis.
**Imaging findings and procedure details:**

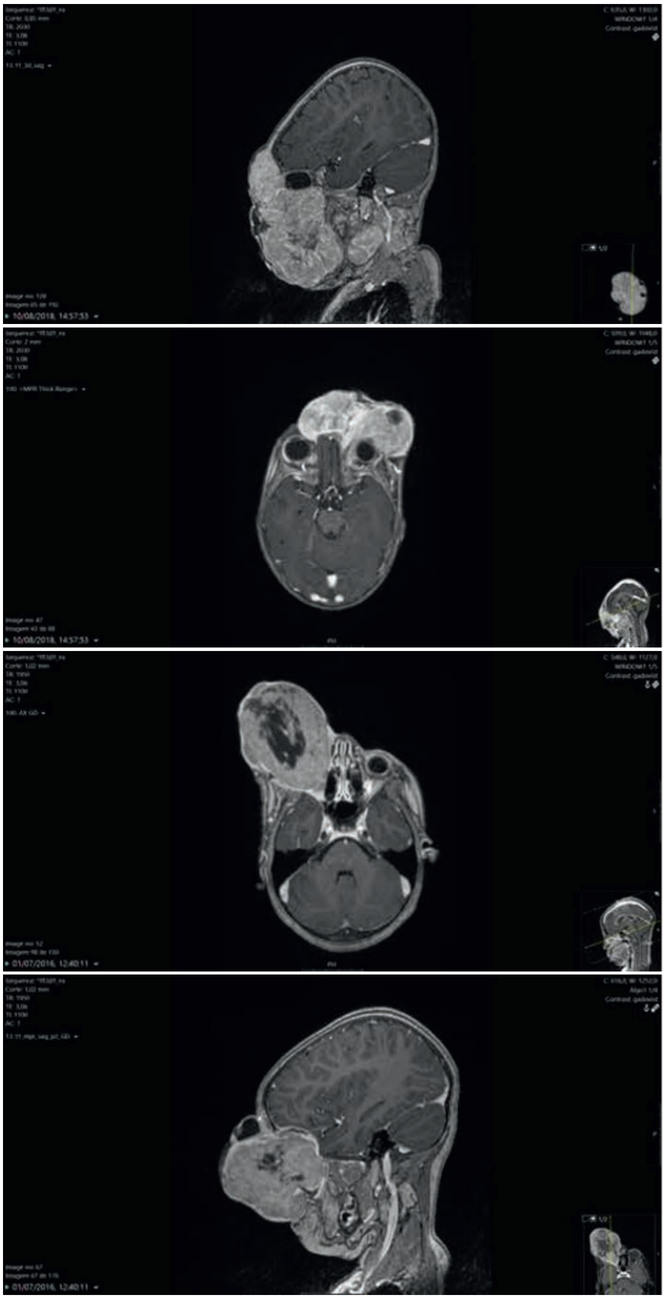

Case 1: female, from Angola. She presented at our center a 11 months-old with a left peri-orbitary rapidly growing mass. On examination she had a large lesion occupying the entire hemiface, without visualization of the left eye. The lesion had an ulcerated and necrotic appearance, with surrounding erythema and some bleeding areas.
At MRI the lesion had signal characteristics suggesting hypercellularity and a high nucleus/cytoplasm ratio, with significant and relatively homogeneous enhancement after gadolinium and some necrotic areas.
After the biopsy she was diagnosed with alveolar rhabdomyosarcoma.
She was treated with chemotherapy and surgery. After 6 months she had tumor recurrence at the nasal pyramid and underwent surgical excision. She continued treatment with chemotherapy and radiotherapy.
Case 2: female, from Guiné-Bissau. She presented at our center at 8 years-old with 2 month evolution of a large and extensive right orbital mass with total proptosis of the eye.
MRI showed a large intra and extra-orbital expansive lesion, with regular and well defined limits, intense enhancement and a cystic-necrotic component. After biopsy she was diagnosed with a parameningeal Embryonic rhabdomyosarcoma. She underwent chemotherapy, radiotherapy and two tumor excision surgeries. Due to several post-surgical complications, a definitive tarsorrhaphy was made.
**Conclusion:** The diagnose of orbital rhabdomyosarcoma is a challenge, as its rapid growth makes it very difficult to distinguish between infectious and inflammatory orbital diseases.
A multidisciplinary approach, including neurorradiology and pathology, is crucial for early diagnosis.

**Keywords:** rhabdomyosarcoma, orbital, pediatric cancer.

### Highlights of Sinonasal Computed Tomography

*Authors: E. Marín-Díez[1], J. Viera-Artiles[2], E. Marco de Lucas[2], D. Castanedo Vázquez[2]; [1]Hospital Universitario Marqués de Valdecilla, Radiología, Santandear, Spain, [2]Hospital Universitario Marqués de Valdecilla, Santandear, Spain*

**Learning objectives:**
1. Review the radiological techniques for the sinonasal region: Computed Tomography (CT), Magnetic Resonance Imaging (MRI), and Cone Beam Computed Tomography (CBCT).
2. Provide a detailed description of sinonasal radiological anatomy.
3. Structured reporting in the sinonasal CT.

**Background:**
Preoperative CT is mandatory in surgical planning before endoscopic surgery. Sinonasal CT allows the chance to identify anatomic variants that predispose patients to surgical complications. These critical areas, however, are not regularly described in the preoperative radiological report.
This educational presentation will explain normal anatomy and highlight common anatomic variants of the paranasal sinuses, emphasising those predisposing to surgical complications.

**Imaging findings and procedure details:**
IMAGING TECHNIQUES:
- CT: best modality.
- MRI: provides an excellent characterization of soft tissues.
- CBCT: first applied to dentomaxillofacial radiology.

WHEN TO PERFORM IMAGING STUDIES
Table 1 shows the main imaging studies indicated for different sinonasal diseases according to the American College of Radiology (ACR).
RADIOLOGICAL ANATOMY
1. NASAL CAVITY
- Nasal septum: parts, classifications (Cottle areas), and morphology.
- Turbinates: superior, middle (emphasis in basal lamella), and inferior.
- Ostiomeatal complex and variants.
- Uncinate process and its different insertions.

2. PARANASAL SINUS
- Ethmoid bone and the olfactory fossa (Keros classification).
- International Frontal Sinus Anatomy Classification. Frontal sinus.
- Maxillary sinus.
- Sphenoid sinus: pneumatization degree and its relations (optic nerve, carotid canal, V2, vidian nerve).
- Pterygopalatine fossa: content, perineural spread.

3. STRUCTURED REPORTING

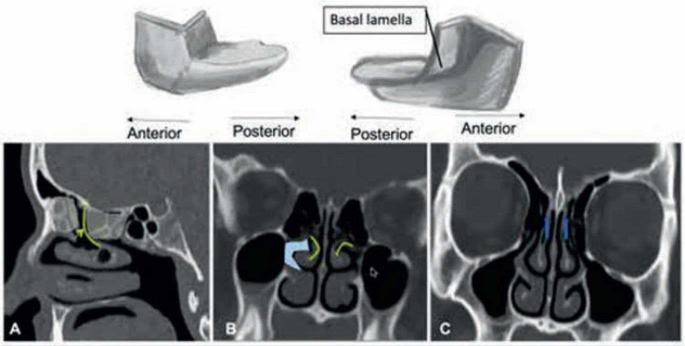

**Anatomy of the middle meatus.** The basal lamella is pointed with the green arrow in A. The blue area in B shows the middle meatus. The blue arrows in C point at the superior part of the middle meatus which inserts into the cribriform plate.

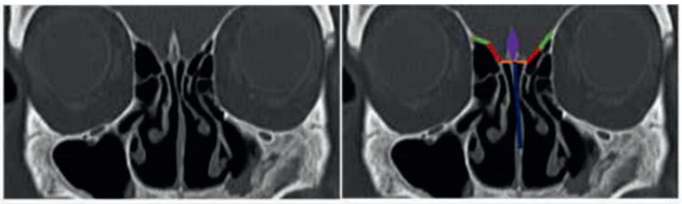

**Ethmoid bone´s anatomy:** horizontal plate in orange (cribriform plate); vertical part in purple for the Crista Galli and in blue for the vertical plate; lateral lamella in red; and fovea ethmoidalis in green (the roof).

**Conclusion:** It is mandatory for the radiologist to know the normal anatomy of sinonasal CT. The routine utilization of preoperative CT allows the opportunity to identify important anatomic variants related to surgical complications.

**Keywords:** Structured Reporting; Sinonasal; Sinonasal CT

### Horner's Syndrome. Beyond the eyes

*Authors: Y. Rodríguez, S. Medrano-Martorell, C. Pineda-Ibarra, S. González-Ortiz, N. Bargalló; Hospital Clinic de Barcelona, Neuroradiology, Barcelona, Spain*

**Learning objectives:**
Review the anatomy the oculosympathetic pathway (OSP) across the imaging findings associated with Horner's syndrome.

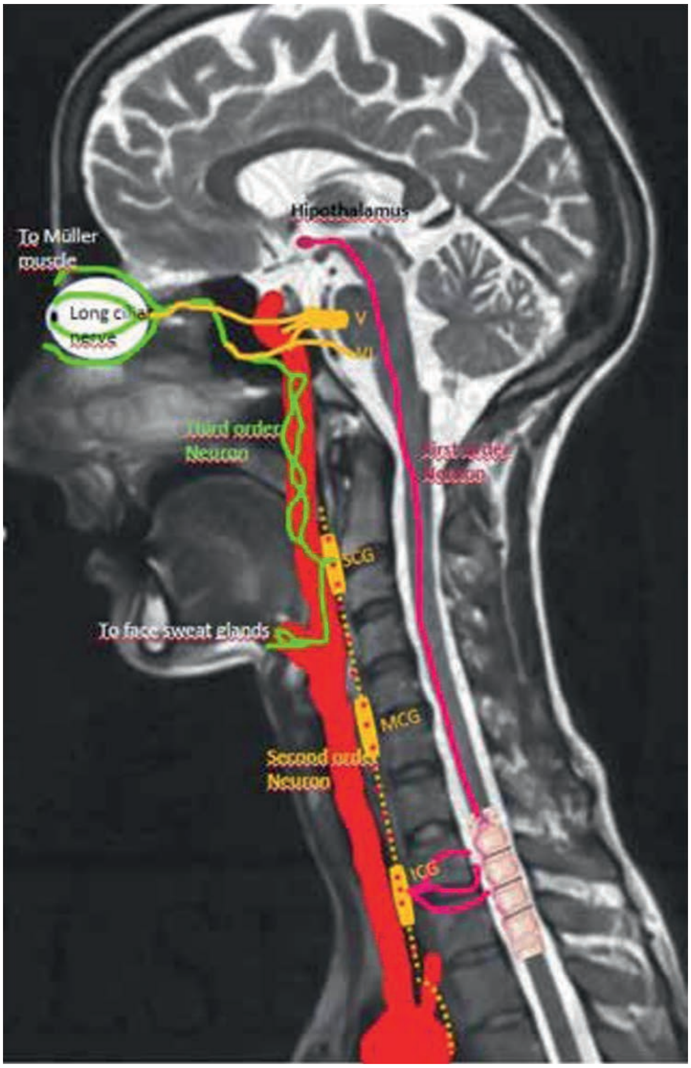

Anatomical pathway of the sympathetic supply to the pupil and eyelids. SCG, superior cervical ganglion; MCG, middle cervical ganglion; ICG, inferior cervical ganglion.

**Background:** Horner`s syndrome (HS) is characterised by the classic triad: miosis, ptosis and hemifacial anhidrosis. It results from an interruption of the sympathetic nerve supply to the eye can be affected in three points: central (first order neurons FON), preganglionic (second order neurons SON) and postganglionic (third order neurons TON) lesions. The clinical symptoms of HS may cause little if any functional impairment in some patients. Is required an adequate clinical evaluation to determine whether chest computed tomography (CT), carotid angiography, head and neck CT or cervical spine or brain magnetic resonance images (MRI) should be performed.
Is suggested to divide it into pre- and postganglionic groups based on pharmacologic testing to determine the location in the OSP with reference to the superior cervical ganglion, all this to make profitable the radiological explorations although some cases the cause of HS may not be found. T
Patients with FON HS and brain or brain stem symptoms should be evaluated with MRI of the brain, if the patient has a FON HS without brain or brain stem symptoms, the first areas evaluated with an MRI should be the cervical and upper thoracic spine.
Patients with lesions in the spine (C8-T2), nerve roots, or neck can produce SON HS symptoms, therefore is recommendable to do scans (CT or MR) of the neck that include the superior cervical ganglion (angle of the mandible/C2-C3) to T2.
If a lesion cannot be localized clinically and imaging is requested, should be do a facial scan (CT or MR) that include the orbit and the superior cervical ganglion (angle of the mandible/C2-C3).
**Imaging findings and procedure details:** We retrospectively reviewed the CT and MR images performed for Horner's Syndrome in our institution over the last 5 Years (2018-2023) and present a pictorial review of the findings.
**Conclusion:** In the presence of a Horner´s syndrome, clinicians and radiologist should be reminding the anatomy of the oculosympathetic pathway. Once a lesion is localized clinically within the OSP by a combination of physical examination and pharmacological testing, the radiologic examination can be appropriately tailored.

**Keywords:** Horner's Syndrome, Horner, sympathetic, oculosympathetic pathway.

### Illustrative and Cross Sectional Review of the Head and Neck Anatomical Spaces

*Authors: M. Abdur-Rahman[1], S. Hegde[2], I. Ansari[3], S. Qureshi[4]; [1]St George's University, London, United Kingdom, [2]Massachusetts General Hospital, Boston, United States of America, [3]American School Dubai, Dubai, United Arab Emirates, [4]SSMC / Mayo Clinic Abu Dhabi, Abu Dhabi, United Arab Emirates*

**Learning objectives:**
To review head and neck anatomical spaces on cross sectional imaging.
**Background:** Head and neck imaging tends to be met with trepidation by residents. This primarily emanates from the associated complex anatomy. Purpose of this presentation is to review the anatomical spaces in the neck. These spaces are demonstrated with anatomical illustrations, supported by cross sectional images. Pathologies within these areas are reviewed.
**Imaging findings and procedure details:** This presentation includes:
Review of the normal anatomical spaces of the neck.
Discussion and diagrammatic illustration of the division of the spaces by fascial layers.
Description of compartments, such as the perivertebral, masticator, parotid and the pharyngeal mucosal spaces.
Cross sectional review of normal and pathological imaging findings within these compartments utilizing CT and MR.
Main differential diagnoses within each compartment.
**Conclusion:** A careful and analytic assessment of the various neck anatomical compartments allows a systematic review head and neck images. After reviewing this presentation, the radiologist will be able to understand the normal anatomical spaces and then be able to assess the location of head and neck pathologies on CT and MR images.

**Keywords:** Head Neck Anatomy Cross Section

### Imaging evaluation of dysphagia after radiotherapy for treatment of head and neck cancer

*Authors: A. Forjaco, J. Lopes Dias; Centro Hospitalar Universitário Lisboa Central, Radiology, Lisbon, Portugal*

**Learning objectives:** To understand the functional consequences of radiotherapy in swallowing function; to recognise the most common pathologic findings in barium swallow studies performed in dysphagic patients; to understand the ultimate indications for swallow therapy and gastrostomy in these patients.
**Background:** Radiotherapy (with or without concurrent chemotherapy) is the workhorse for organ-preservation in the treatment of many pharyngeal and laryngeal cancers. Severe dysphagia is a late effect that may develop years after radiation-based therapy for head and neck cancer.
Swallowing function may be impaired due to oedema, neuropathy, and fibrosis. Acute toxicities such as mucositis and oedema disrupt normal swallowing during treatment but improve substantially in the months following therapy. Neuropathy and fibrosis of the oral, laryngeal, and pharyngeal musculature may persist long after the completion of treatment, and have been implicated as the primary mechanisms of long-term radiation-induced dysphagia.
**Imaging findings and procedure details:** Barium swallow studies are used as standard methods to evaluate swallowing function. Pathologic findings in radiation-induced dysphagia include: pharyngeal residue and laryngeal penetration or aspiration; pharyngeal or oesophageal strictures; functional anomalies affecting the laryngeal elevation, epiglottic deflection, tongue base retraction, anterior hyoid excursion, and pharyngeal contraction.
The most feared complications related to these alterations are nutritional compromise, and aspiration pneumonia, with consequent need of hospitalization. These are also the most common indications for gastrostomy. Swallow therapy may improve pharyngeal transit and airway protection, with the development of compensatory techniques to maintain oral intake and prevent aspiration.
**Conclusion:** Severe dysphagia is a late complication of radiotherapy-based treatment in long-term survivors of head and neck cancer. It is critical to understand the functional effects of radiotherapy in swallowing function, to recognise the pathologic findings in barium swallow studies performed in these patients, and to understand the indications for swallow therapy and gastrostomy.

**Keywords:** radiation-induced dysphagia; barium swallow studies

### Imaging features of Ludwig's angina

*Authors: T. Perillo[1], M. De Giorgi[2], M. Perrotta[1], A. Vitale[1], A. Serino[1], A. Manto[1]; [1]"Umberto I" Hospital, Department of Neuroradiology, Nocera Inferiore, Italy, [2]University of Naples Federico II, Department of Advanced Biomedical Sciences, Naples, Italy*

**Learning objectives:**
To describe main imaging findings of Ludwig's angina.
**Background:** Ludwig's angina is a life-threatening condition with diffuse cellulitis involving the soft tissues of the floor of the mouth and neck, thus possibly the submandibular, sublingual and submental spaces. The most frequent cause is tooth infection, especially of the lower molars[1]. It may have a rapid progression and cause complications such as airway obstruction and carotid artery rupture[2].
MR is superior to CT scan in evaluating soft tissue and neck compartment involvement but, especially in emergency, a contrast enhanced-CT (CECT) is the best imaging choice.
**Imaging findings and procedure details:** Imaging helps us in the search of abscesses and cellulitis in the oral cavity, evaluating its localization (one or more spaces), dimensions and morphology. Best imaging tool is the CECT with a routine cervical soft tissue neck study: soft tissue window to identify abscess location, bone window for tooth infection or osteomyelitis

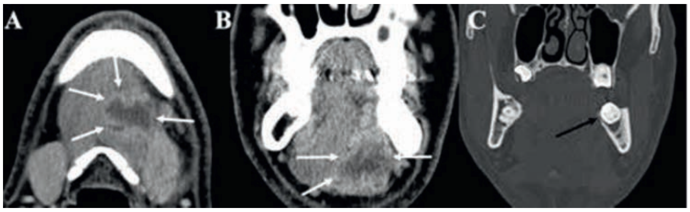

Contrast-enhanced CT with soft tissue window on the axial plane (A) and coronal reformat (B) and bone window coronal reformat show a rim-enhancing fluid collection in the left sublingual space, consistent with abcess (white arrows in A and B). There is a tooth decay of the 38 which was the cause of the infection (black arrow in C).

[3]. The abscess presents as a rim-enhancing fluid collection and can involve the mentioned spaces, having the mylohyoid muscle and midline as landmarks

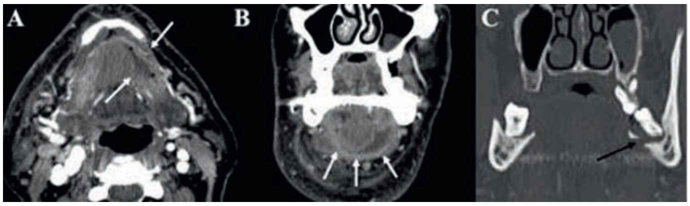

Contrast-enhanced CT with soft tissue window on the axial plane (A) and coronal reformat (B) and coronal bone reformat (C ) show and abscess in the left sublingual space (white arrows in A and B) which extends in the right sublingual space (arrows in B). The cause of the infection was a tooth decay of the 38 (black arrow in C).

; cellulitis will present with adjacent soft tissue stranding and dermal thickening. MR findings are high signal of focal fluid collection in T2 sequences and restricted diffusion for the abscess, with rim-enhancement and low signal in fatty marrow of mandible at site of infected tooth in T1 images.
Ultrasonographic findings shows a hypoechoic collection within oral cavity musculature and can favor the needle aspiration for diagnosis simultaneously.
**Conclusion:** Ludwig's angina is a life-threatening condition with trans-spatial abscess involving submandibular, sublingual and submental spaces. Imaging allows, especially with CECT, its timely identification and can evaluate its extension, also characterizing the components of the lesion. An early and punctual diagnosis in this condition can be decisive for an effective therapy.

**Keywords:** Ludwig's Angina; MR; CT; Abscess

References:
[1] Francis B. Quinn Jr, MD, (1999), Ludwig angina, Arch Otolaryngol Head Neck Surg, 125(5):599, 10.1001/archotol.125.5.599
[2] Bansal A, Miskoff J, Lis RJ, (2003), Otolaryngologic critical care, Crit Care Clin, 55-72, 19(1), 10.1016/s0749-0704(02)00062-3
[3] Feredy Gunawan, Widiana Ferriastuti , (2022), Ludwig's angina: An alarming radiology challenge, Elsevier, Radiology Case Report

### Imaging findings of head and neck vascular anomalies in adults

*Authors: I. Adic[1], A. Spasic[1,2], D. Hadnadjev Simonji[1,2], M. Stankov[1,2]; [1]Faculty of Medicine, University of Novi Sad, Novi Sad, Serbia, [2]Centre for radiology, Clinical Centre of Vojvodina, Novi Sad, Serbia*

**Learning objectives:**
- to present radiological features of head and neck vascular anomalies in adults;
- to illustrate the imaging differences between benign vascular tumors and vascular malformations in adults.

**Background:** Vascular lesions have a varied appearance and can commonly occur in the head and neck region. The International Society for the Study of Vascular Anomalies (ISSVA) classification system divides vascular anomalies into two major groups: vascular tumors and vascular malformations. Vascular tumors are lesions with neoplastic endothelial mitotic activity whereas vascular malformations only have abnormal morphogenesis.
Vascular tumors are divided into benign, locally aggressive/borderline and malignant. Hemangiomas are benign vascular tumors, of which the two most common are infantile and congenital hemangiomas. The most common borderline vascular tumor is Kaposiform hemangioendothelioma. Angiosarcomas are rare malignant vascular tumors which can occur in any age group.
Vascular anomalies are divided into simple, combined, malformations of major named vessels and malformations associated with other anomalies. Simple malformations are capillary, lymphatic, venous, arteriovenous malformations and arteriovenous fistulas. Combined malformations are two or more simple malformations present in one lesion.
**Imaging findings and procedure details:** The imaging modalities for head and neck vascular anomalies include ultrasound, computed tomography and magnetic resonance imaging. For most types of anomalies application of contrast is required to determine vessel involevement.
Ultrasound is usefull in initial exams to depict the size and vascularity of the lesion. Computed tomography allows evaluation of vascular and bone structures as well as the presence of phlebolites, while magnetic resonance imaging provides the greatest tissue specificity of the lesion.
Hemangiomas are intermediate or high T2 MRI signal with internal flow voids, usually with early postcontrast enhancement.
Lymphatic malformations are macro- or microcystic structures, with high T2 signal and fluid-fluid levels. They are no- or low flow lesions. After contrast administration they show low or no contrast enhancement.
Venous malformations are low flow lesions, with high T2 signal and tubular shape, sometimes with phlebolites. They show enhancement on delayed phase.
Arteriovenous malformations and fistulas are high flow malformations, with low T1 and T2 signal due to flow voids.
**Conclusion:** Head and neck vascular anomalies are common findings and a correct diagnosis is crucial due to the possibility of involvement of numerous organ systems. Advanced imaging modalities are used to demonstrate the extent and the nature of the lesion, which are important for treatment options.

**Keywords:** vascular anomalies, vascular malformations, vascular tumors

### Imaging of common orbital neoplasms in adults

*Authors: I. Adic[1], D. Hadnadjev Simonji[1,2], A. Spasic[1,2], M. Stankov[1,2]; [1]Faculty of Medicine, University of Novi Sad, Novi Sad, Serbia, [2]Centre for radiology, Clinical Centre of Vojvodina, Novi Sad, Serbia*

**Learning objectives:**
- to present the defining imaging features of the most common orbital neoplasms;
- to help radiologists recognize benign and malignant features of common orbital neoplasms.

**Background:** Knowledge of the anatomy of the orbit is essential when imaging orbital masses. The orbit can be anatomically divided by the rectus muscles into extraconal and intraconal compartments. The extraconal compartment consists of the lacrimal gland, fat and the bony orbital walls. The globe, optic nerve, fat and the orbital vessels are located intraconally. Orbital neoplasms can be categorized based on their location. Some neoplasms manifest in a single compartment while others can be found in multiple compartments.
Cavernous malformations are most common benign orbital lesions. They are slow growing masses, presenting with progressive, painless proptosis.
Lymphoma is the most common orbital lymphoproliferative neoplasm. Although any part of the orbit can be involved, the superolateral extraconal space is the most affected. Patients usually present with proptosis and limited eye movement.
Most common intraconal tumors are optic nerve and nerve sheath tumors. Primary optic nerve sheath meningeomas arise from intraorbital part of the optic nerve. They are less frequent than secondary meningeomas which occur intracranialy and extend into the orbit. They are slow growing, with eventual nerve atrophy due to compressive mass effect.
Primary orbital melanoma is the most common primary intraocular malignant neoplasm in adults. Symptoms include visual field defects and decreased vision.
**Imaging findings and procedure details:** Cross-sectional imaginag plays a vital role in detecting and evaluating extraocular orbital neoplasms. Computed tomography and magnetic resonance imaging are most frequently used.
Cavernous malformations are well demarcated, T2W homogeneous lesions, most commonly arising intraconally where they displace surrounding structures. On postcontrast imaging they show centripetal pattern on delayed images.
Lymphoma usually molds to orbital structures. Lesions are T1W isointense relative to intraorbital muscles and relatively T2W hyperintense to orbital fat, displaying uniform postcontrast enhancement.
Optic meningeomas typically show the expansion of the nerve sheath in a „tram-track“ configuration depicted on axial postcontrast images, with the nerve spared.
Choroidal melanomas are imaged on MRI where they show high T1W and low T2W signal, as well as the extent of extraocular disease.
**Conclusion:** Orbital neoplasms are a varied group of benign and malignant entities. Radiological imaging, especially MRI, allows precise depiction of the extent of disease. Clinical symptoms, radiological features as well as the compartement of the orbit involved allow radiologists to make a correct diagnosis.

**Keywords:** orbital neoplasm, orbit anatomy, magnetic resonance imaging

### Magnetic resonance imaging of anterior ischemic optic neuropathy

*Authors:* *R. G. Yoon, D. H. Kim; University of Eulji College of Medicine, Nowon Eulji Medical Center, Department of Radiology, Seoul, Republic of Korea (South Korea)*

**Learning objectives:**
The increasing use of magnetic resonance imaging (MRI) necessitates that radiologist understand the pathophysiology and imaging findings of anterior ischemic optic neuropathy (AION). In this educational pictorial review, we highlight the pathophysiology and imaging features on various MRI sequences for evaluating AION
**Background:** AION is thought to result from ischemia in the perilaminar region of the optic nerve, which is supplied by the posterior ciliary artery. AION is categorized into nonarteritic (NAION) or arteritic (AAION), caused by ischemic injury of the optic nerve head. The increasing number of older adults with higher risk factors for NAION, such as hypertension, diabetes, smoking, and atherosclerosis, makes it necessary to understand the pathophysiology and MRI findings of AION. Although fundoscopy and visual field testing remain the primary diagnostic tools, high-resolution MRI sequences can provide enhanced visualization of the optic nerve and surrounding structures, enabling a comprehensive evaluation of the orbit. This educational poster focuses on the application of recent high-resolution and advanced imaging techniques for evaluating AION.
**Imaging findings and procedure details:** MRI plays a role in differentiating AION from other causes of optic neuropathy, such as compressive, demyelinating, or inflammatory disease. Optic nerve head swelling is a common finding in AION due to accumulation of fluid in the optic nerve head. AION can be detected on 3-dimensional (3D) contrast-enhanced (CE) T1-weighted image (T1WI) with fat saturation and diffusion-weighted imaging (DWI), which show focal enhancement and focal diffusion restriction at the optic disc. Especially, DWI can identify early changes in the optic nerve that are not visible on T1- or T2-weighted images (WI). CE-3D-T2-FLAIR can demonstrate additional contrast enhancement which might be missed on 3D-CE-T1WI or other conventional MRI sequences. In giant cell arteritis patients, 3D-CE-T1WI allows detection of arteritic posterior ciliary artery involvement in patients with AAION. A recent study using intravoxel incoherent motion (IVIM) demonstrated that IVIM may reflect the perfusion abnormality and visual function impairment in NAION patients.
**Conclusion:** We illustrated the imaging findings of AION on various MRI sequences. These imaging sequences can provide complementary information about optic nerve damage, differentiating AION from other causes of optic neuropathy, which enable early diagnosis and prompt management. Radiologists need to be familiar with the pathophysiology of AION and the application of various high-resolution and advanced MRI sequences in the evaluation of patients with suspected AION

**Keywords:** Orbit; Ischemic Optic Neuropathy; Magnetic resonance imaging

References:
[InvRad] Nora N. Sommer, Karla M. Treitl, Eva Coppenrath, Hendrik Kooijman, Claudia Dechant, Michael Czihal, Theresa M. Kolben, Sebastian E. Beyer, Wieland H. Sommer, Tobias Saam, (2018), Three-Dimensional High-Resolution Black-Blood Magnetic Resonance Imaging for Detection of Arteritic Anterior Ischemic Optic Neuropathy in Patients With Giant Cell Arteritis, Wolters Kluwer Health, Inc, Investigative Radiology
[JMRI] Ping Lu 1 2, Yan Sha 2, Hailin Wan 2, Feng Wang 2, Guohong Tian 3, Wenlin Tang 4, (2017), Assessment of nonarteritic anterior ischemic optic neuropathy with intravoxel incoherent motion diffusion-weighted imaging using readout-segmented echo-planar imaging, parallel imaging, and 2D navigator-based reacquisition, International Society for Magnetic Resonance in Medicine

### Magnetic resonance imaging of salivary glands lesions: Pearls and pitfalls

*Authors:* *M. Fiannacca[1,2], F. Borda[3], S. Caprioli[3], N. Romano[2], A. Castaldi[2], G. Cittadini[3]; [1]Galliera Hospital, Genoa, Italy, Neuroradiology Department, Genoa, Italy, [2]Galliera Hospital, Neuroradiology, Genoa, Italy, [3]San Martino Hospital, Radiology, Genoa, Italy*

**Learning objectives:**
To explain the main features of different types of salivary gland tumors in Magnetic Resonance Imaging (MRI), based on morphological and functional analysis, making attention on the overlap between malignant and benign lesions.
**Background:** Salivary gland tumors constitute a heterogeneous group and can be divided into primary or secondary lesions and malignant or benign ones. MRI is useful to evaluate morphological and functional features of focal salivary gland lesions such as signal
intensity on T1- and T2-weighted images and functional analysis by DWI and ADC map values, dynamic contrast enhanced curves derived from perfusion weighted imaging (DCE-PWI) and analysis of contrast enhancement types. MRI is fundamental to localize the lesion and to evaluate the margins, the internal composition and the extension into adjacent different anatomical structures (vessels, nerves, bones, muscles).
MRI allows to characterize and differentiate among different lesions, even if there are also some limits on this analysis, because some benign and malignant lesions have some overlapping features that don't allow to find out the correct diagnosis. For example, among benign lesions we have pleomorphic adenoma that have typical round shape, regular margins, hypointense signal in T1W sequences, high signal in T2W images, moderate contrast enhancement, high values in ADC map (≥ 1.4 x 10-3 mm2/s), DCE-PWI curves types A or rarely C.
**Imaging findings and procedure details:** Based on literature and scientific knowledge, we herein review the main types of focal salivary gland lesions and their typical imaging findings in MRI showing different clinical cases.
**Conclusion:** Salivary gland lesions include a large and heterogeneous group of tumors, and radiologists, despite the difficulties in differential diagnosis, plays a pivotal role during the diagnostic path.

**Keywords:** MRI, salivary glands, lesions

### Masses in salivary glands region: A series of cases of common and misleading lesions

*Authors:* S. K. J. Flores Quispe; *Hospital Ca' Foncello Treviso, Department of Radiology, Treviso, Italy*

**Learning objectives:**
- To overview the wide array of glandular lesions and tumors of other origin.
- To illustrate multimodality imaging findings of a case series collected in our institution.

**Background:** Among head and neck tumors, salivary gland tumors (SGT) represent only the 1%. Most salivary gland tumor are benign with ~ 70% arising in major glands. The most common location is the parotid gland, comprising 60-75% of all cases. There is a wide array of different histology, due to the epithelial and the non-epithelial histology[1]. The 2017 WHO classification of salivary gland tumors recognizes twelve types of benign epithelial SGT, such as pleomorphic adenoma, Warthin tumor, basal cell adenoma, with histologic subtypes that differ in recurrency and/or malignant transformation. For malignant epithelial SGT, twenty different tumor types are identified, such as mucoepidermoid carcinoma, adenoid cystic carcinoma and acinic cell carcinoma. Hematolymphoid tumor include Hodgkin lymphoma, diffuse large B cell lymphoma, extranodal marginal zone B cell lymphoma.

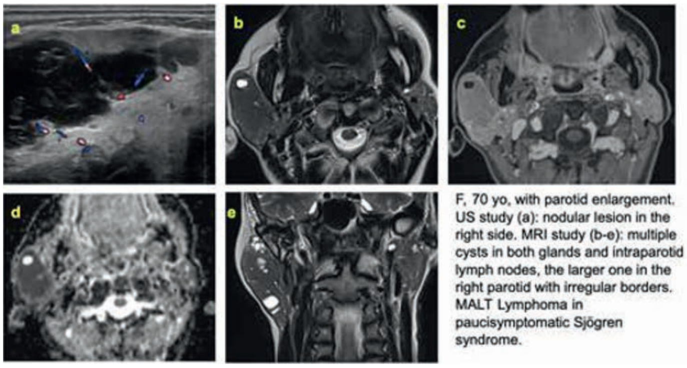

F, 70 yo, with parotid enlargement. US study (a): nodular lesion in the right side. MRI study (b-e): multiple cysts in both glands and intraparotid lymph nodes, the larger one in the right parotid with irregular borders. MALT Lymphoma in paucisymptomatic Sjögren syndrome.

Non-epithelial tumors are rare, among them mesenchymal tumors such as hemangiomas and lymphangiomas are more common in pediatric population than in adults. Nevertheless, inflammatory condition may affect salivary glands leading to a mass effect [2].
Correct diagnosis is essential in dictating the proper management.

**Imaging findings and procedure details:** Ultrasonography (US) is usually the first modality due to low cost and with high sensitivity in detecting masses in superficial tissues. Computerized tomography (CT) represents a prompt availability imaging modality in urgent cases, such as abscess, in which it may detect the underlying etiology.

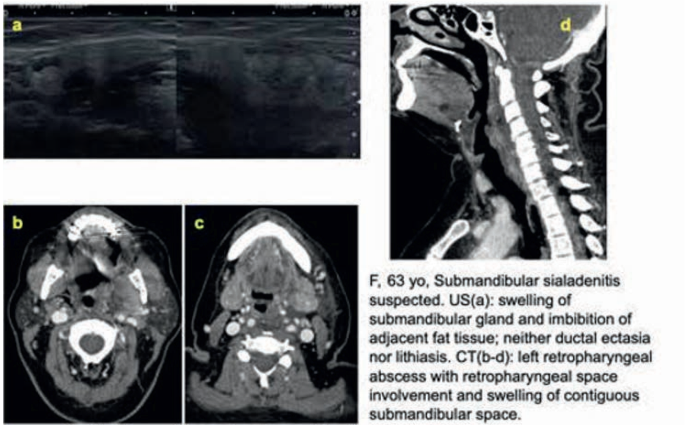

F, 63 yo, Submandibular sialadenitis suspected. US(a): swelling of submandibular gland and imbibition of adjacent fat tissue; neither ductal ectasia nor lithiasis. CT(b-d): left retropharyngeal abscess with retropharyngeal space involvement and swelling of contiguous submandibular space.

Magnetic resonance imaging (MRI) is the modality of choice, provides a superior soft tissue contrast compared to CT, permits to describe the exact localization and boundaries and in case of malignancies allows to detect local infiltration and perineural spread[3][4]. Finally, MRI specific protocols have shown results in differentiation between benign and malignant SGT (i.e. gadolinium-enhanced dynamic sequences[5]).
This poster illustrates a series of cases of common and misleading lesion.

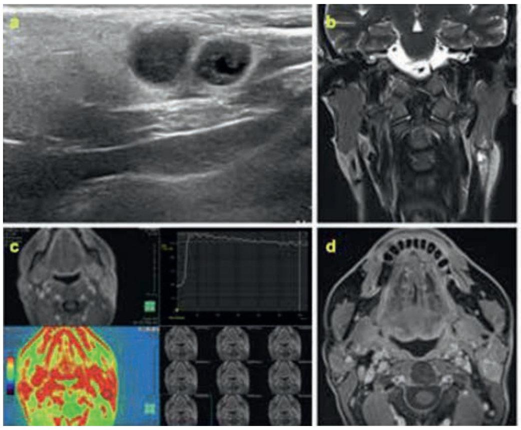

F, 62 yo, Laterocervical swelling. Story of basal cell carcinoma. US (a) detection nodular lesions. MRI (b-d): definition of malignant lesion, with Plateau pattern enhancement curve. Diagnosis of lymphadenopathy.

**Conclusion:** The knowledge of neck anatomy, pathologies that can affect the salivary glands and anamnestic and clinical data, are helpful in the correct diagnosis and proper management.

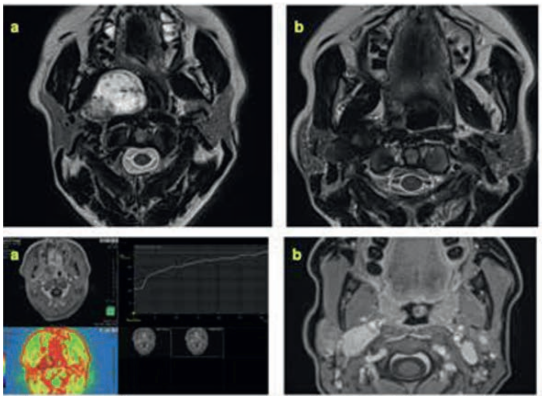

(a) pleomorphic adenoma (b)paraganglioma. the knowledge of the anatomy allows the correct localization

**Keywords:** salivary gland

References:
[1] Alsanie I, et al., Distribution and Frequency of Salivary Gland Tumours: An International Multicenter Study
[2] Allen Young; Oluwafunmilola T. Okuyemi., Malignant Salivary Gland Tumors
[3] Harriet C. Thoeny, Imaging of salivary gland tumours
[4] H Coudert, S Mirafzal, A Dissard, L Boyer, P-F Montoriol, Multiparametric magnetic resonance imaging of parotid tumors: A systematic review
[5] Hidetake Yabuuchi, Takeshi Kamitani, Koji Sagiyama, Yuzo Yamasaki, Tomoyuki Hida, Yuko Matsuura, Takuya Hino, Yuriko Murayama, Ryuji Yasumatsu , Hidetaka Yamamoto, Characterization of parotid gland tumors: added value of permeability MR imaging to DWI and DCE-MRI

### Most common extracranial otitis media complications

*Authors: M. Latinovic, D. Hadnadjev Simonji, A. Spasic; Clinical Center of Vojvodina, Center for Radiology, Novi Sad, Serbia*

**Learning objectives:**
1. When the radiologic study is required in otitis media and otomastoiditis?
2. What are the extracranial otitis media complications?
3. Present an example of TMJ abscess as a potential complication of prolonged otitis media infection with postponed nasopharyngeal abscessus formation

**Background:** Otitis media is an inflammation of the middle ear. The impaired immune systems of patients with congenital immune deficiencies, HIV infection or diabetes may be involved in the development of otitis media.

**Imaging findings and procedure details:** Infection can travel from the middle ear via the aditus ad antrum in the mastoid air cells, and mastoiditis may occure as a typical complication of acute otitis media and occurs in 0,2-2% of otitis. Acute bacterial (suppurative) otomastoiditis responds to antibiotic treatment. Radiologic study is required only when there is clinical suggestion of coalescent mastoiditis, as coalescence signifies the transition from mucoperiostal disease to bone disease, and eventually to other extracranial and intracranial complications.

Other extracranial complications include the following: subperiostal abscess, petrositis, labyrinthitis, labyrinthine fistule, facial paralisys, cholesteatoma, cholesterol granuloma, tympanosclerosis, tympanic membrane perforation, hearing loss, chronic suppuratice otitis media, infectious eczematoid dermatitis, Bezold's abscess, temporomandibular joint (TMJ) arthritis.

There is a close relationship between middle ear, mastoid cavity, glenoid fossa and TMJ. TMJ is one of the places where inflammatory disease of ear and mastoid process spreads. The bony walls of the pneumatized spaces of the mastoid are important barriers and the presence of open sutures in the glenoid fossa and delayed ossification of tympanic plate can cause the spread of middle ear and mastoid infections into the TMJ. TMJ abscess is probably the most significant radiologic sign of TMJ arthritis.

**Conclusion:** The prognosis for almost all patients with OM is excellent, the exeptions are patients in whome otitis media involves extracranial and intracranial complications (<1%). CT proved to be the diagnostic method of choice for evaluating acute otomastoiditis, locating otitic intra or extracranial complications, and assessing mastoid air cell coalescence and the possibility of subperiosteal abscess, and in combination with clinical examination should dictate following medical treatment.

### Nasal masses: CT, CBCT and MRI findings. A Pictorial review

*Authors: E. Palizzolo[1], A. Inzerillo[1], F. Bencivinni[2], A. Lo Casto[1], M. De Angelis[1], F. Pandolfo[1], G. La Tona[1]; [1]Sezione Scienze Radiologiche, BIND, Università degli studi di Palermo, Palermo, Italy, [2]UOS Radiologia Odontoiatrica e Maxillo-Facciale, Università degli Studi di Palermo AOUP P. Giaccone, Palermo, Italy*

**Learning objectives:**
A review of nasal masses studied by CT, CBCT and MRI in our Department is presented, describing characteristic imaging features, that can supply a clue for differential diagnosis, together with endoscopic and pathology correlation when available.

**Background:** Various diseases ranging from benign to malignant ones, congenital to acquired, infectious or tumoral, may arise in the nose. Different symptoms are complained by patients with a nasal mass and its diagnosis maybe challenging. Nasal endoscopy, diagnostic imaging and histopathology are employed conjointly to reach the diagnosis, that involves sometimes very uncommon lesions. CT or CBCT is usually the first imaging line, while for better definition and differential diagnosis in doubtful cases MRI with contrast agent is the choice.

**Imaging findings and procedure details:** Different types of lesions involving the nasal cavities were studied by CBCT or CT, and/or MRI with contrast agent. Referring to non neoplastic lesions, rinoliths, inverted mesiodens and septal mucocele are reported. Referring to benign and borderline tumors, lobular capillary hemangioma, lipoma, osteoma, hamartoma, haemangioma, hemangiopericytoma, juvenile angiofibroma, cemento-ossyfing fibroma, antrochoanal polyp, hemorrhagic polyp, inverted papilloma are reported. Referring to malignant neoplasms intestinal type adenocarcinoma, esthesioneuroblastoma, non-Hodgkin lymphoma, melanoma, sarcoma are reported. CBCT or CT is useful in depicting subtle bone anatomy of nasal cavity and adjacent sinuses and other anatomic structures, moreover supplying a navigation map for eventual endoscopic sinus surgery, whereas MRI is more useful to define different soft tissue component within the lesion. Contrast agent administration adds more specificity to differential diagnosis, in relation to vascular pattern.

**Conclusion:** Lesions of the nose are one of the most challenging conditions that otolaryngologists must diagnose and treat. Imaging features of nasal masses can help in the differential diagnosis before surgical removal. Nasal endoscopy with biopsy remains the gold standard for diagnosis.

### Nasal Obstruction: What the radiologist should report

*Authors: E. Marín-Díez[1], D. Castanedo Vázquez[2], E. Marco de Lucas[2], J. Viera-Artiles[2]; [1]Hospital Universitario Marqués de Valdecilla, Radiología, Santandear, Spain, [2]Hospital Universitario Marqués de Valdecilla, Santandear, Spain*

**Learning objectives:**

1. Review the technical and anatomical considerations of sinonasal Computed Tomography (CT).
2. Provide an image-based review of common and uncommon causes of nasal obstruction (NO), focusing on unilateral nasal obstructions in adults.

**Background:** We have reviewed the relevant literature covering nasal anatomy and nasal obstruction (NO) in otolaryngological and rhinological imaging.
The common causes of NO are highlighted and the diagnostic approach is explained.

**Imaging findings and procedure details:**
DEFINITION:
Nasal obstruction refers to the subjective sensation of reduced airflow through the nasal cavity and is reported to affect up to one-third of the population. It is a very common symptom, both at the primary care units and in patients referred to otolaryngologists with symptoms that are refractory to medical treatment.
IMAGING STUDIES:
Computed tomography (CT)
Magnetic resonance imaging (MRI)
ETIOLOGIC FACTORS OF NASAL OBSTRUCTION:
A. MUCOSAL CAUSES
- Chronic rhinosinusitis without polyps
- Chronic rhinosinusitis with polyps
- Allergic fungal rhinosinusitis

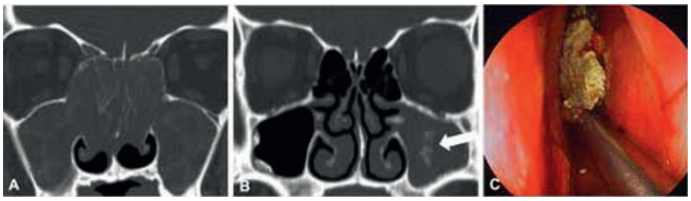

**Fungal rhinosinusitis.** A and B, coronal sinus CT. C is a nasal endoscopy. Image A shows allergic fungal rhinosinusitis where the characteristics are similar to chronic rhinosinusitis with polyps but expansion and hyperdensity are more prominent. Image B shows a sinus mycetoma, typically affecting only a single sinus. Arrow points at a soft tissue density within the sinus with foci of calcific deposits. Image C shows a sinus mycetoma or a fungus ball in nasal endoscopy.

B. STRUCTURAL CAUSES
Nasal septum
Turbinates
C. TRAUMATIC CAUSES
D. OTHER CAUSES
Non-neoplastic nasal masses
Neoplastic nasal masses

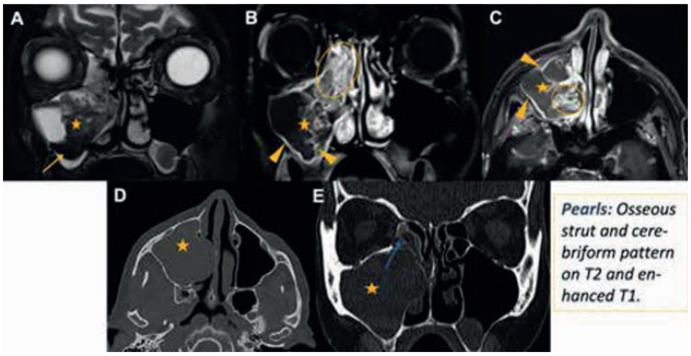

**Inverted papilloma.** A, coronal T2WI (FS); B, coronal T1WI (FS) with contrast; C, axial T1WI (FS) with contrast; D-E, axial and coronal CT scans. Right maxillary antrochoanal heterogeneous mass expanding the primary ostium into the right nasal cavity and right anterior ethmoid air cells (stars in A-E). The periphery of the lesion in the right lateral maxillary sinus is very T2 hypointense (yellow arrow in A), diffusion restricted (not shown) with edge enhancement (yellow arrowheads in B and C) and as it extend medially demonstrates more solid cribriform enhancement (yellow circles in B and C). There is a focal area of hyperostosis near the inferior margin of the lamina papyracea/floor of the right orbit compatible with bony origin of inverted papilloma (blue arrow in E).

**Conclusion:** Rhinitis and rhinosinusitis are the most common causes of nasal obstruction. Imaging is usually performed after optimal medical treatment. The principal aim of CT is determining the distribution and severity of mucosal disease and describing relevant anatomical variants that contribute to the narrowing of the sinus drainage or have implications for endoscopic surgery. Mass lesions are an uncommon cause of nasal obstruction. MRI is required to increase the specificity of the diagnosis of a mass, delimit its extent, evaluate local spread, and assess for perineural involvement.

**Keywords:** Sinonasal; Nasal Obstruction;

### Nasal septal perforation – A pictorial review

*Authors: J. Asogan Vaishnavi[1], A. Kamalasanan[2], T. Sudarshan[1]; [1]NHS Tayside Ninewells Hospital and Medical School, Clinical Radiology, Dundee, United Kingdom, [2]NHS Lanarkshire, Clinical Radiology, Glasgow, United Kingdom*

**Learning objectives:**
1. Radiological anatomy of nasal septum and its clinical relevance
2. Clinical presentation of nasal septal perforation and the role of imaging
3. Pictorial review of various causes of nasal septal perforation with a systematic approach
4. What radiologists need to know while reporting nasal septal perforation

**Background:** Nasal septal perforation, by definition, refers to a communication between the two nasal cavities via a defect in the nasal septum. With the rising prevalence of nasal septal perforation, it is imperative for radiologists to be aware of the various types of nasal perforations and their underlying causes. The main intent of this exhibit is to familiarise the readers with the various common and rare causes, with special emphasis on what to be addressed while reporting such cases, which can eventually help in decisions of management. Our educational exhibit compiles all the causes and imaging findings, including radiological manifestations in other systems, under one heading via a structured pictorial review.

**Imaging findings and procedure details:** The initial modality of choice for evaluation of nasal septal perforation is CT, by acquiring 0.6mm slices with minimal overlap and multi-planar reconstruction. Further evaluation may be done with contrast enhanced CT and/or MRI, and depends on the underlying cause. Based on the location, perforations are classified into anterior (most common), posterior and superior types. Anterior type is mostly due to trauma while the other two are commonly caused by systemic diseases. The aetiologies of nasal septal perforation include trauma (iatrogenic/digital trauma), intra-nasal drugs (cocaine/steroids/industrial chemicals), infections (fungal/tuberculosis/syphilis/leprosy), vasculitis & autoimmune diseases (Granulomatosis with polyangitis/Systemic Lupus Erythematosis/Sarcoidosis) and neoplasms (malignant tumours & granulomas). In cases where the underlying cause can have systemic involvement, correlation with other modalities such as CT Chest, etc may be warranted to aid in the diagnosis. Surgical treatment, with flaps and grafts, are usually employed in patients whose symptoms are refractory to conservative management.

While reporting, mentioning the following aspects can help in planning the treatment:
1) Perforation position - Anterior / Posterior / Superior
2) Perforation size - Two dimensional measurement
3) Underlying cause
4) Comment on remaining osseocartilaginous support of nasal septum, lateral nasal wall, nasal floor and turbinates (aids in surgical technique selection)

**Conclusion:** Imaging plays a vital role in nasal septal perforation by identifying the type of perforation, the underlying cause and finally with treatment decision.

**Keywords:** nasal septal perforation, nasal septum anatomy

### Nasopharyngeal invasion in chronic lymphocytic leukemia: A case report

*Authors: R. de Sousa[1], F. Rodrigues[1], C. Chaves[1], P. Barradas[1], B. Marques[2], S. Carvalho[1]; [1]Centro Hospitalar e Universitário de Coimbra, Neurorradiologia, Serviço de Imagem Médica, Coimbra, Portugal, [2]Centro Hospitalar e Universitário de Coimbra, Serviço de Hematologia, Coimbra, Portugal*

**Learning objectives:**
We aim to demonstrate the role of imaging in the detection of head and neck manifestations of chronic lymphocytic leukemia (CLL), namely the affection of the Waldeyer's ring.

**Background:** CLL is the most common leukemia in adults in Western countries, affecting more frequently older individuals. Although its primary manifestation is the accumulation of mature B- cells within the blood, with lymphocytosis, any lymphoid tissue may be involved including Waldeyer's ring, an extranodal site that include the palatine, tubal, lingual and pharyngeal tonsils.

Although the head and neck region is frequently involved in manifestations of CLL, leukemic infiltration besides generalized lymph node enlargement is extremely rare, even more with nasopharyngeal invasion. This new entity is named Nasal Associated Lymphoid Tissue (NALT) CLL.

**Imaging findings and procedure details:** We report a 63-year-old caucasian female, with known RAI stage 3 CLL, no del17p or TP53 mutation present, but IGHV mutated, who presented to the emergency department with a worsening holocranial headache for 1 month, refractory to analgesia, and tinnitus associated with ear fullness on the left side. Emergency computed tomography (CT) showed nasopharyngeal soft tissue thickening bilaterally with slight erosion of the adjacent clivus. The patient was hospitalized for further investigation and monitorization.

Magnetic resonance imaging (MRI) confirmed thickening of the soft tissue of the nasopharynx with deep retropharyngeal extension, hypointense on T1 and T2-WI, with no diffusion restriction and with homogeneous contrast enhancement.

Otolaryngology detected effusion in the left middle ear in relation with Eustachian tube dysfunction caused by the described lesion.

Biopsy of the nasopharyngeal lesion with immunophenotyping and pathological analysis confirmed invasion by the underlying lymphoproliferative disease.

Patient started therapy including Obinutuzumab- venetoclax, until now with no complications.

**Conclusion:** We report a symptomatic presentation of CLL, in an unusual localization, associated with auditory symptoms. Although rarely encountered, radiologists should be vigilant for the possibility of leukemic infiltration of the nasopharyngeal mucosa in CLL, as it is considered a disease-related complication, with implications, mainly, in treatment selection.

**Keywords:** CLL, chronic lymphocytic leukemia, Waldeyer's ring, hematology, Otolaryngology, nasopharynx

### Orbital infections: Periorbital and Orbital cellulitis

*Author: M. T. Figueroa Simonsen; Aalborg University Hospital, Radiology, Aalborg, Denmark*

**Learning objectives:**
- Recognize the imaging modalities used to diagnose orbital infections.
- Identify the typical radiological findings of periorbital and orbital cellulitis.

**Background:** Orbital infections are a potentially serious disease that can cause vision loss or even blindness if left untreated. It is typically caused by bacterial infection, most commonly Staphylococcus aureus or Streptococcus pyogenes. Early diagnosis and prompt treatment are essential to prevent complications. Radiological imaging plays a critical role in diagnosing and monitoring the condition.

**Imaging findings and procedure details:** Computed tomography (CT) and magnetic resonance imaging (MRI) are the most commonly used imaging modalities for periorbital and orbital cellulitis. CT scans can identify the extent of the infection, the involvement of the sinuses, and any associated abscess formation. MRI is particularly useful in cases where the infection has spread to the central nervous system. On both imaging modalities, periorbital cellulitis appears as soft tissue swelling, with possible involvement of the extraocular muscles and orbital fat. In severe cases, orbital cellulitis can lead to proptosis and optic nerve compression.

**Conclusion:** Periorbital and orbital cellulitis is a serious condition that requires prompt diagnosis and treatment. Radiological imaging is an essential tool for identifying the extent of the infection and monitoring the patient’s response to treatment. CT and MRI are the most commonly used imaging modalities and can provide valuable information on the severity of the infection and the involvement of adjacent structures. Recognizing the typical radiological findings of periorbital and orbital cellulitis is critical for an accurate diagnosis and treatment of these conditions.

**Keywords:** periorbital, orbital cellulitis, CT, MR

### Ossicular Chain Discontinuity

*Authors: C. Á. Póka[1], H. Sükösd[2]; [1]Semmelweis Egyetem, Orvosi Képalkotó Klinika, Budapest, Hungary, [2]Semmelwei Egyetem, Orvosi Képalkotó Klinika, Budapest, Hungary*

**Learning objectives:** In this educational poster, our aim is to summarize the etiology of ossicular chain discontinuity, and depict the radiomorphological features using high-resolution computed tomography.

**Background:** The middle ear’s sound-conduction apparatus consist of three ossicles, the malleus, the incus and stapes, joined by two synovial joints, the incudomallear and incudostapedial joints.
The apparatus transmits vibratory stimulus from the tympanic membrane, and amplifies them towards the surface of the oval window.
Ossicular chain discontinuity (also called ossicular chain dislocation) is loss of normal alignment between the three middle ear ossicles, which can be easily missed. Complete loss of this conductive pathway would result in a conductive hearing loss.
Discontinuity can occur following trauma, but can occur due to cholesteatoma, chronic middle ear infection or congenital disease, usually due to erosion of the ossicles.

**Imaging findings and procedure details:** This poster demostrates the most common causes and appearances of ossicular chain disruption with high resolution temporal bone CT images.

**Conclusion:** Detecting and accurately describing the subtle findings of ossicular chain disruption is essential for optimal surgical approach and result.

**Keywords:** ossicular chain discontinuity, dislocation, conductive hearing loss

### Otomastoiditis – A practical approach to ear infections

*Authors: A. Alade, X. Kowa, E. Pilavachi, A. Timmis, T. Beale; University College London Hospital, Radiology, London, United Kingdom*

**Learning objectives:** Severe ear infections can potentially be life-limiting conditions and rely heavily on imaging for diagnosis [1]. The aim of this poster is to highlight:
- Imaging features of aggressive temporal bone infections and their complications
- Understanding the range of organisms driving these infections
- Radiological differential diagnoses

**Background:** Ear infections can be broadly subdivided into otitis externa, otitis media and mastoiditis depending on the affected part(s) of the temporal bone. The number of cases of ear infections in the United Kingdom varies widely. Necrotising otitis externa (NOE) is a rare condition with an incidence of 0.001% [2]; otitis media is a far commoner condition affecting up to 80% of preschool children with concurrent upper respiratory tract infections [3]. There are significant differences in how these conditions are diagnosed, managed, and their overall morbidity and mortality. The radiologist plays a key role in advising the clinician of the necessity of time-sensitive radiological investigations, the modalities best suited to this purpose, guidance regarding optimal site(s) for tissue sampling, and the opportunity to identify potential mimics of aggressive infective processes.

**Imaging findings and procedure details:** We provide a practical approach to the radiology vetting process with an emphasis on 'red flag' symptoms and our institution's cross-sectional imaging techniques. We will discuss the role of image-guided diagnostic intervention. We will share a range of cases of aggressive ear infections highlighting the findings indicating severe disease: bone destruction, abscess formation, vascular thromboses, and other intra- and extracranial complications. Critically, we will provide tips on how the various conditions can be distinguished as patient demographics may be misleading. We will discuss the responsible organisms as the profile of microbes implicated in middle ear and mastoid infections differ from NOE. Our cases of NOE include confirmed growth of pseudomonas, fungi and other rarer organisms (actinomycosis). Shared cases will also include mimics of aggressive infections e.g., cholesteatoma, osteoradionecrosis, aggressive tumours (squamous cell carcinoma, sarcoma) and autoimmune disease.

**Conclusion:** Severe ear infections are associated with high morbidity and mortality rates. NOE in particular can be challenging to diagnose often resulting in delays commencing appropriate antimicrobial therapy. We utilise a multidisciplinary team (radiology-infectious disease-otology) approach at our institution and aim to share our experience in the management of these conditions.

**Keywords:** otology, infection, necrotising otitis externa, mastoiditis

References:

[1] Agnieszka Trojanowska, Andrzej Drop, Piotr Trojanowski, Katarzyna Rosińska-Bogusiewicz, Janusz Klatka, and Barbara Bobek-Billewicz, (2012), External and middle ear diseases: radiological diagnosis based on clinical signs and symptoms, Springer, Insights Imaging, 33–48, 3(1): , https://www.ncbi.nlm.nih.gov/pmc/articles/PMC3292638/, 2023-04-02

[2] Necrotising otitis externa: A single centre experience Mehdi Hasnaoui, Asma Ben Mabrouk, Jihene Chelli, Fatma Larbi Ammari, Rihab Lahmar Adnene Toumi, Khalifa Mighri , (2021), Necrotising otitis externa: A single centre experience, Science Direct, Journal of Otology, 22-26, Volume 16, Issue 1, https://www.sciencedirect.com/science/article/pii/S1672293020300659, 2023-04-02

[3] Amina Danishyar, John V. Ashurst, (2022), Acute Otitis Media, StatPearls Publishing LLC, StatPearls [Internet], Treasure Island (FL), https://www.ncbi.nlm.nih.gov/books/NBK470332/#_NBK470332_pubdet_, 2023-04-02

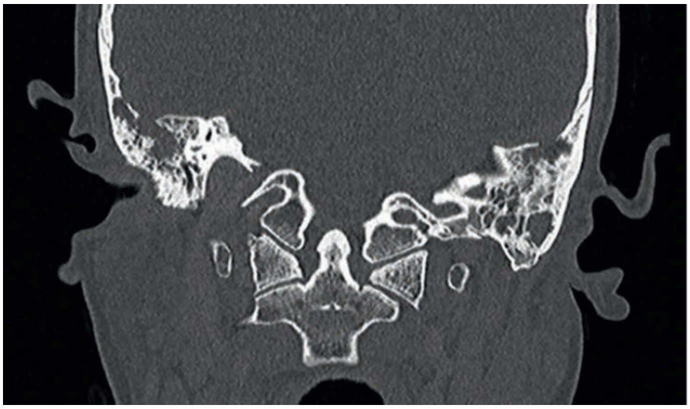

**Case 1.** Bilateral acute coalescent mastoiditis in a 41 year old male. This presentation is less common in adults. Coronal CT demonstrates complete opacification of the mastoid air cells on both sides with resorption of the mastoid trabeculae (extension of mucoperiosteal disease to involve bone) resulting in defects of the tegmen tympani.

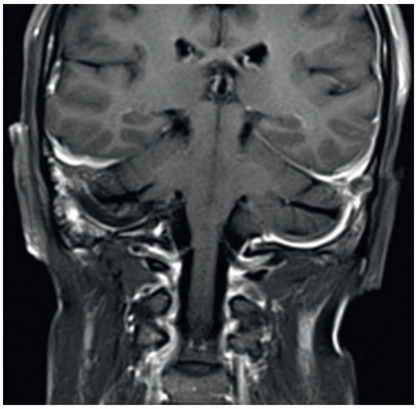

**Case 1.** Bilateral acute coalescent mastoiditis in a 41 year old male. Coronal post-contrast fat-suppressed MRI demonstrates the presence of a peripherally-enhancing subperiosteal collection at the site of an eroded posterosuperiorly located left mastoid air cell directly abutting the sinus. Extensive pachymeningeal thickening.

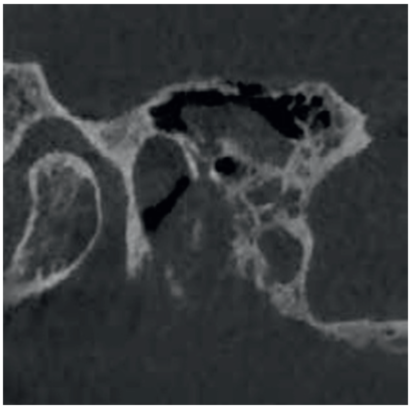

**Case 2.** NOE in a 76 year old male of no fixed abode with multiple comorbidities including hypertension and diabetes. Sagittal CBCT of the left temporal bones. In contrast to the previous case, whilst the mastoid is also opacified and eroded, the epicentre of destruction localises to the posterior wall and floor of the external auditory canal.

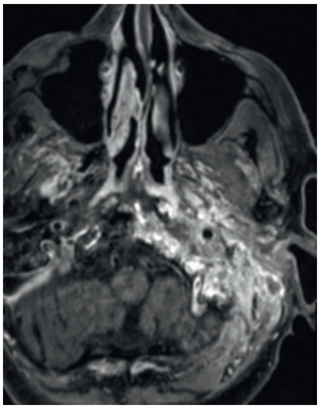

**Case 2.** Post-contrast fat-suppressed MRI shows transpatial inflammation of the skull base, parotid, carotid, parapharyngeal, prevertebral and masticator spaces. This manifested initially as facial swelling and a 7th cranial nerve palsy with the patient referred to radiology for workup of a suspected parotid mass. NOE was diagnosed on imaging.

### Parotid space: Navigating through the uncommon lesions

*Author: M. S. Swarup; Vardhman Mahavir Medical College and Safdarjung Hospital, Radiology, New Delhi, India*

**Learning objectives:**
1. To provide an image based review of various unusual and rare lesions involving the parotid space.
2. To highlight the characteristic imaging findings if any.

**Background:** The parotid gland is involved in wide variety of lesions including congenital, inflammatory and neoplastic processes. It can give rise to typical benign lesions like Pleomorphic adenoma and Warthin's tumour and common malignant lesions like Mucoepidermoid carcinoma and Adenoid cystic carcinoma. However; Parotid space is the site for development of various unusual and rare lesions, which can be correctly diagnosed on imaging.
We will attempt to present few unusual or uncommon lesions involving parotid and periparotid region. A multimodality imaging approach including ultrasonography, CT and MRI will be used. The characteristic imaging findings of each entity will be briefly discussed.

**Imaging findings and procedure details:**
CASES:
(1) Benign neoplastic lesions:
- Lipoma
- Haemangioma
- Facial nerve Schwannoma
- Pleomorphic adenoma in accessory parotid tissue

(2) Malignant neoplastic lesions:
- Hodgkin's Lymphoma involving parotid
- Metastatic involvement
- Uncommon primary salivary gland malignancies
- Rhabdomyosarcoma involving parotid

(3) Inflammatory/Infective/Auto-immune lesions:
- Benign lymphoepithelial lesion (HIV associated)
- Tubercular involvement of parotid gland / Granulomatous disease
- Sjogren's syndrome
- Kimura disease

(4) Miscellaneous lesions:
- Venous vascular malformation involving parotid
- Lymphangioma involving parotid
- Branchial cleft cyst
- Mucous retention cyst
- Epidermal inclusion cyst
- Benign intra-parotid lymph nodal enlargement
- Intra-parotid pseudo-aneurysm
- Pneumoparotitis

**Conclusion:** Parotid gland lesions are a common clinical dilemma. Imaging plays a central role in detection, characterization and extension of parotid lesions. Radiologists should be aware of these uncommon or rare parotid lesions and should be familiar with their imaging appearances. Some of the lesions do not have specific imaging appearances and radiologists must remain attentive to clinical history and FNAC/ biopsy to guide the correct diagnosis.

**Keywords:** Parotid gland, salivary gland, lymphoepithelial lesion

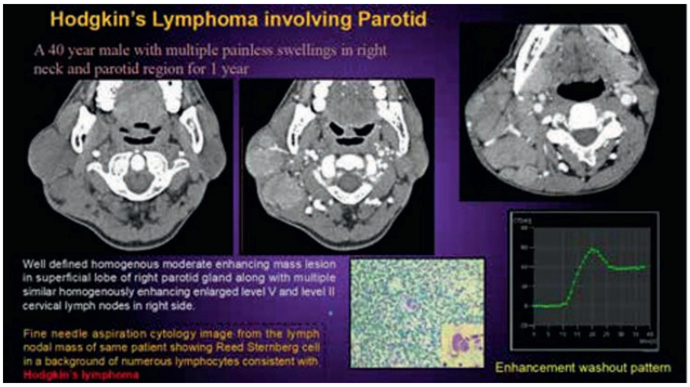

Hodgkin's Lymphoma involving Parotid

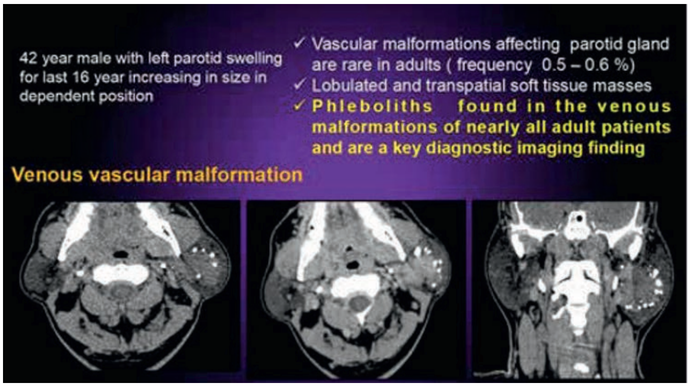

Venous vascular malformation involving parotid

### Patterns of perineural spread in nasopharyngeal carcinoma: A pictorial guide for trainees

*Authors:* *N. S. Vanjavaka, S. Kumar, B. Purohit; National Neuroscience Institute, Singapore, Neuroradiology, Singapore, Singapore*

**Learning objectives:**
1. This educational poster aims to familiarise junior trainees/residents with the various imaging patterns of perineural spread (PNS) in nasopharyngeal carcinoma (NPC).
2. To highlight commonly seen involvement of the pterygopalatine fossa, Vidian nerve, V2, V3 and 12th cranial nerve with case-based imaging examples.
3. To describe a checklist of certain crucial anatomical sites which must be scrutinized carefully while reporting a case of NPC, so as to avoid underdiagnosis of PNS.

**Background:** NPC has high prevalence in Southern China and South East Asia. At our tertiary neuroscience centre, we get routine referrals for imaging the skull base in known/suspected cases of NPC.
NPC originates in the fossa of Rosenmuller and spreads to the nearby spaces such as nasal cavity, paranasal sinuses, skull base foramina etc. Presence of PNS indicates poor prognosis, higher chances of recurrence and can also alter treatment plan.
A structured approach to evaluate certain anatomic sites, such as the pterygopalatine fossa, Vidian canal, foramen rotundum, foramen ovale, jugular foramen and hypoglossal canal is therefore essential so as to detect clinically silent PNS.

**Imaging findings and procedure details:**
We retrospectively reviewed all previously reported MRI brain and skull base studies performed for NPC at our institute, from January 2014 to January 2023. We evaluated the various patterns of PNS detected and reported in known cases of NPC.
Various imaging appearances of PNS were identified, such as obliteration of fat in neural foramina, widening and enhancement of neural foramina, thickening, nodularity and abnormal enhancement especially in the pterygopalatine fossa, along the Vidian nerve, V2, V3 and occassionally, hypoglossal nerve. Neural enhancement was well demonstrated on MRI, whereas the bony erosions and widening of neural foramina were better assessed on CT. Some of the cases also presented with denervation atrophy of muscles supplied by involved cranial nerves, again better evaluated on MRI.

**Conclusion:** It is crucial for the reporting radiologist to be aware of the likely paths and patterns of PNS in NPC. The sites of involvement and imaging appearances must be mentioned in detail in the report, so as to provide a detailed roadmap to the surgeon for adequate clinical management. Since a lot of these scans are often primarily read and reported by trainees, it is important that they are aware of the clinical importance of this entity.

**Keywords:** nasopharyngeal carcnioma, perineural spread, pterygopalatine fossa, vidian nerve, foramen rotundum, foramen ovale

### Pictorial review – Differential diagnosis of head and neck lesions based on their space of origin

*Authors:* *K. Borbély, A. Dienes, P. Maurovich-Horvat, H. Sükösd; Medical Imaging Centre, Radiology, Budapest, Hungary*

**Learning objectives:**
Our aim was to review computer tomography (CT) anatomy, anatomical relations, and content of the deep neck spaces in the suprahyoid and infrahyoid neck to be able to localize neck pathology into specific spaces which helps in generating the best differential diagnosis.

**Background:** The neck is an anatomically complex region with multiple complex pathologies including benign to malignant lesions as well as life threatening infections. The neck region can be divided into specific spaces. When a mass is identified in the neck, knowledge about the anatomy and content of these spaces narrows the spectrum of differential diagnosis, helps in identifying the extension of the lesion and aids in the accurate diagnosis. CT has a primary role in the diagnosis of neck pathologies, compared to MRI it is widely available and has the advantage of short acquisition time. Multiplanar reconstructions of axial images allows a precise assessment of structural involvement and extensions of neck pathologies.

**Imaging findings and procedure details:** The deep spaces of the neck extends from the base of the skull to the anterior superior mediastinum. It is divided into the suprahyoid and infrahyoid regions by the hyoid bone and further anatomically partitioned by the superficial, middle and deep layers of the deep cervical fascia into different spaces. There are seven suprahyoid spaces (parapharyngeal space, pharyngeal mucosal space, parotid space, masticator space, buccal space, sublingual space, submandibular space) two infrahyoid spaces (visceral space, anterior cervical space) and five spaces (carotid space, retropharyngeal space, danger space, perivertebral space, posterior cervical space) that cross the hyoid bone and extend across the entire neck. Using CT images acquired, with standardized protocols, in our department we demonstrate in detail these anatomical spaces with potential pathological processes originating from their content and their possible route of extension.

**Conclusion:** Knowledge about the anatomy of the deep neck spaces, their content and what pathology could potentially arise from them narrows the differential diagnosis and allows for the best evaluation of any lesion in the neck region. Furthermore, reporting using accepted deep neck space terminology allows for better communication between radiologists and head and neck specialists.

**Keywords:** Deep neck spaces, imaging anatomy, suprahyoid neck, infrahyoid neck

### Pictorial review of head and neck lymphomas

*Authors: E. Marín-Díez[1], M. Ravanelli[2], N. Di Meo[2], P. Rondi[2], E. Marco de Lucas[3]; [1]Hospital Universitario Marqués de Valdecilla, Radiología, Santandear, Spain, [2]Ospedale Civile Brescia, Brescia, Italy, [3]Hospital Universitario Marqués de Valdecilla, Santandear, Spain*

**Learning objectives:**
1. To explain the main imaging (MRI and CT) characteristics of head and neck lymphomas.
2. To show a pictorial review highlighting the main localizations and subtypes of head and neck lymphomas.
3. To present the differential diagnosis of the head and neck lymphoma according to the anatomical area affected.

**Background:** We have reviewed the relevant literature covering head and neck lymphomas. The most common localizations are highlighted and the diagnostic approach is explained.

**Imaging findings and procedure details:** Lymphomas represent approximately 5% of all malignant neoplasms of the head and neck.
- The extra-nodal lymphoma is the most common type in the head and neck region, followed by combined nodal and extra-nodal lymphoma. Extra-nodal lymphomas arise from soft tissue other than the lymph nodes. The head and neck region is the second most frequent anatomical site of extra-nodal lymphomas after the gastrointestinal tract. Most are non-Hodgkin's lymphomas of B-cell lineage, and overall diffuse large B-cell lymphoma is the most common type.

In the head and neck, the most common site of non-Hodgkin's lymphomas is Waldeyer's ring. We will review the image characteristics of different cases affecting:
- Tonsils.
- Parotid glands.
- Tongue base.
- Nasopharynx.
- Nasal cavity.
- Soft palate.
- Paranasal sinus.

Local staging can be achieved via contrast enhancement MRI or CT scans of the concerned head and neck region. For systemic staging, either FDG-PET/CT or CT of the neck, thorax, abdomen, and pelvis are the techniques of choice.

**Conclusion:** Head and neck lymphomas represent a heterogeneous group of diseases with highly variable clinical and radiological presentations, histology, and prognosis. The imaging pattern of disease involvement in Waldeyer's ring or other common extra-nodal sites with or without nodal involvement should warn the radiologist about the possible diagnosis of lymphoma.
Familiarity with imaging characteristics is crucial for early diagnosis and specific oncological treatment.

**Keywords:** lymphoma; headandneck

### Pitfalls in staging and in posttreatment surveillance of oral cavity cancer

*Author: M. Horta; Instituto Portugues de Oncologia de Lisboa Francisco Gentil, Radiology, Lisbon, Portugal*

**Learning objectives:** To become familiar with common imaging pitfalls in staging and in the follow up of oral cavity cancer.

**Background:** Oral cavity cancer annual incidence of OSCC in 2020 was 377,713 cases worldwide,being the 16thmost common type of cancer overall. These tumours are classified on the following subsites:upper and lower gingiva (alveolar ridges), lip, buccal mucosa, hard palate, floor of mouth, oral tongue (anterior two-thirds) and retromolar trigone. All of these tumour subsites show typical patterns of spread that the radiologist should become familiar with.

**Imaging findings and procedure details:** MR and CT are complementary modalities that play an important role in the diagnosis and pre-operative staging of oral cancer; thus being crucial tools for defining the surgical and therapeutic approach of these tumors. Ultrasound and intraoral ultrasound have shown to be valuable in the assessment of nodal disease and in the estimation of the depth of invasion. On the other hand, PET-CT has shown to be a reliable tool for locoregional surveillance of oral cancer and for management of locally advanced nodal metastases.

**Conclusion:** To help preventing diagnostic errors and in order to guide appropriate therapeutic management, radiologists should be aware of common mistakes in staging and in post treatment follow up of oral cavity cancer. Knowledge of the existence of these diagnostic pitfalls should help prevent misinterpretation in the assessment of oral cavity cancer.

**Keywords:** oral cavity cancer; MR; CT; Ultrasound

### Points of surgical interest in imaging of tongue malignancy

*Authors: A. Kamalasanan[1], T. Sudarshan[2]; [1]NHS Lanarkshire, Radiology, Glasgow, United Kingdom, [2]NHS Tayside, Radiology, Dundee, United Kingdom*

**Purpose/Objectives:** We aim to discuss imaging anatomy, pathology and TNM staging of oral and oropharyngeal tongue malignancy with discussion centred on imaging details that help surgical decision making, types of surgery and complications.
**Methods and materials:** Imaging review of anatomy of the tongue with imaging examples of TNM staging and route of spread. The maxillofacial surgeons require assessment of the imaging to decide on type of surgery and extent of disease clearance. We will discuss the indication and structures excised and preserved in each surgical procedure. Extensive disease will require free flap reconstructions. We aim to discuss what the surgeons require from radiology reports and imaging to make these surgcal decisions. Discussion of critical striuctures, margins of disease involved with their surgical relevence, surgical techniques with examples will be discussed.

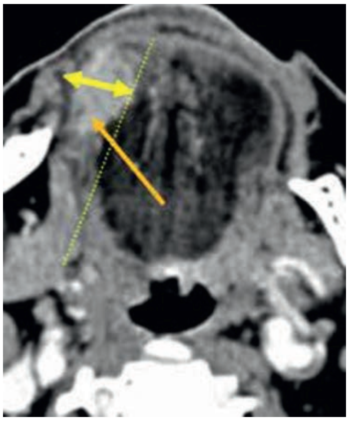

calculating the depth of invasion on imaging

**Result:** Teaching points:
Depth of invasion (DOI) is measured from the level of normal tissue at the basement membrane to deepest limit of enhancement. Radiological DOI is usually more than pathological DOI. In ulcerative lesions, DOI is larger than the lesion. In exophytic lesions DOI, is smaller than the lesion. Elective neck dissection has been proven to increase overall survival and disease free survival in patients with T2 or greater tumors, even with N0 neck . Sentinel node biopsy is done in some centers if DOI is greater than 4mm. On the sagittal plane, at least 1.5cm of normal tongue should be interposed between the tumor and vallecula for larynx preservation. Involvement of mylohyoid or geniohyoid may require *enbloc* resection of primary and neck dissection to avoid positive margin, depending on surgical expertise. There are differences in AJCC and UICC staging protocols. Despite removal from UICC TMN oral tongue staging, involvement of extrinsic muscles and lateral pharyngeal wall, pterygo-mandibular raphae should be highlighted in the report as they alter the surgical plan.
**Conclusion:** Malignancy of the tongue remains a disease with high morbidity. Accurate assessment of the disease and critical structures are needed for ideal management.

**Keywords:** Tongue, SCC, malignancy , TNM, surgery

References:
[2] Joo YH, Cho JK, Koo BS, Kwon M, Kwon SK, Kwon SY, Kim MS, Kim JK, Kim H, Nam I, Roh JL, Park YM, Park IS, Park JJ, Shin SC, Ahn SH, Won S, Ryu CH, Yoon TM, Lee G, Lee DY, Lee MC, Lee JK, Lee JC, Lim JY, Chang JW, Jang JY, Chung MK, Jung YS, Cho JG, Choi YS, Choi JS, Lee GH, Chung PS. , (2019), Guidelines for the Surgical Management of Oral Cancer: Korean Society of Thyroid-Head and Neck Surgery, Clinical and Experimental Otorhinolaryngology, https://doi.org/10.21053%2Fceo.2018.01816
[ref1] Anil K D'Cruz 1, Richa Vaish, Neeti Kapre, Mitali Dandekar, Sudeep Gupta, Rohini Hawaldar, Jai Prakash Agarwal, Gouri Pantvaidya, Devendra Chaukar, Anuja Deshmukh, Shubhada Kane, Supreeta Arya, Sarbani Ghosh-Laskar, Pankaj Chaturvedi, Prathamesh Pai, Sudhir Nair, Deepa Nair, Rajendra Badwe; Head and Neck Disease Management Group, (2015), Elective versus Therapeutic Neck Dissection in Node-Negative Oral Cancer, New England Journal of Medicine, https://doi.org/10.1056/nejmoa1506007
[ref3] Rana M, Iqbal A, Warraich R, Ruecker M, Eckardt AM, Gellrich NC., (2011), Modern surgical management of tongue carcinoma - A clinical retrospective research over a 12 years period, Head and neck Oncology, 10.1186/1758-3284-3-43
[ref4] Kato MG, Baek CH, Chaturvedi P, Gallagher R, Kowalski LP, Leemans CR, Warnakulasuriya S, Nguyen SA, Day TA, (2020), Update on oral and oropharyngeal cancer staging – International perspectives, World Journal of Otorhinolaryngology - Head and Neck Surgery, doi.org/10.1016%2Fj.wjorl.2019.06.001

### Post COVID-19 period associated complications of acute Rhinosinusitis in children: A case report

*Authors: F. Rodrigues[1], R. Sousa[1], C. Chaves[1], R. Pais[1], J. Neves[2], C. Maia[1], P. Barradas[1], F. Bartolomeu[2]; [1]Centro Hospitalar e Universitário de Coimbra, Serviço de Imagem Médica, Neurorradiologia, Coimbra, Portugal, [2]Centro Hospitalar e Universitário de Coimbra, Serviço de Otorrinolaringologia - Hospital Pediátrico, Coimbra, Portugal*

**Learning Objectives:** Our goal is to demonstrate the severity and imaging findings of a case of complicated Acute rhinosinusistis after the peak COVID-19 period taking into consideration the possibility that mask wearing period can have diminished the immune training and prowess of pediatric age individuals and that in 2022 (year of our case report) there were 6 children that had chirurgical approach comparing to 3 during the 2018-2021 period in our centre.
**Background:** We report a 16-year-old caucasian male with a previous diagnosis of rhinosinusitis 2 weeks prior to admission that was treated with amoxicillin. He presented to the emergency department with extensive left orbital swelling with closure of the upper eyelid with superior extension encompassing the Squama frontalis. He also had purulent drainage from the middle meatus bilaterally. Computed tomography (CT) showed pansinusitis with diffuse thickening of the frontal sinus mucosa and extensive edema of the left eyelid with superior orbital involvement and even slight extension of the process to the intracranial component. This case required multidisciplinary intervention by ophthalmology, neurosurgery, and otolaryngology. Following surgical intervention and a 3-week course of intravenous antibiotic therapy, with no identified microbial agent. The patient had resolution of the infection with no neurologic sequelae.
**Imaging Findings or Procedure Details:**

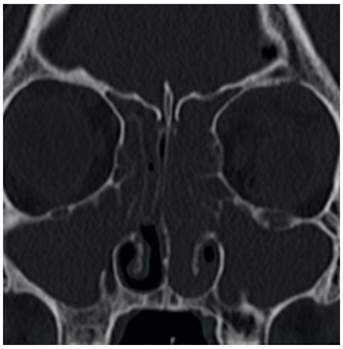

Figure 1: CT: Pansinusitis, with practically complete filling of the different sinus cavities, with only slight airing at the posterior ethmoid and left sphenoid level.

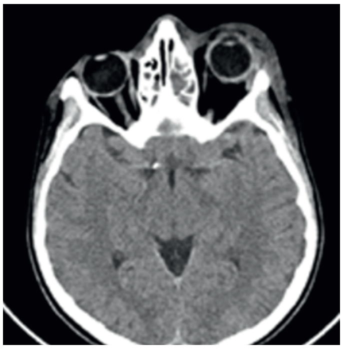

Figure 2: CT: Extensive eyelid edema on the left, with involvement of the orbit, at the level of the upper quadrants, in pre- and post-septal extra-conical topography. Extensive edema of the soft tissues of the nasal and anterior frontal region, with small gas foci.

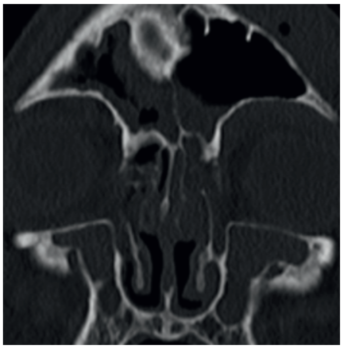

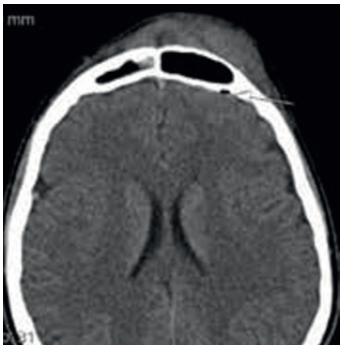

Figure 3 and 4: CT: Discrete bone thinning at the level of the left fronto-ethmoidal transition and on the medial side of the right frontal sinus at the level of the anterior wall. Extradural collection, millimeter, which contains a small gas bubble. There is extension of the process to intracranial compartment, eventually through the vascular/nervous physiological channels

**Conclusion:** This case describes rare, but potentially life-threatening, complications of acute rhinosinusitis that were more frequent in our center after the COVID-19 pandemic mask use.
Diagnosis and management of orbital and intracranial complications are challenging, and close collaboration among different specialties is required. Early imaging is critical and has a major role not only to make the diagnosis but also to indicate the best first approach, whether it is urgent surgery or optimized medical treatment.

**Keywords:** Acute rhinosinusitis, Complicated rhinosinusitis, Orbital complication, Intracranial complication, Post-Covid 19

### Post treatment MRI of nasopharyngeal carcinoma: Tips and Tricks

*Authors: H. Hafsi, M. Gharbi, S. Esseghaier, Y. Arous; Hopital militaire de Tunis, Radiology, Tunis, Tunisia*

**Learning Objectives:**
- To learn the place of MRI in post treatment evaluation of Nasopharyngeal cancer.
- To differentiate residual / recurrent nasopharyngeal tumors from fibrosis.
- To identify the complications related to treatment.

**Background:** Nasopharyngeal carcinoma is one of the most common malignancies of the head and neck. This disease arises in endemic regions and has a close relation with EBV virus. These tumors are particularly radiosensitive and chemosensitive. MRI has an important place in post treatment locoregional follow-up. We will describe some tips and tricks in making radiological diagnosis of common post treatment changes and how to differentiate them from radiation-induced complications and residual or reccurent tumors. [1]

**Imaging Findings and Procedure Details:** Diffusion-weighted imaging is the technique of choice for making radiological diagnosis of residual or recurrent tumors and differentiate them from radiation-induced changes.

The main post-irradiation changes are mucositis, salivary glands atrophy, sinusitis and otomastoiditis, radiation induced osteitis. [2]
The main radiation-induced complications can be divided into radiation-induced nervous system injuries, radiation-induced neoplasms, osteoradionecrosis. [2]

**Conclusion:** MRI is the key tool for post treatment follow up of nasopharyngeal carcinoma. Familiarization with common post treatment changes is necessary for early diagnosis radiotherapy-induced complications and residual or recurrent disease. [3]

**Keywords:** Nasopharyngeal carcinoma, magnetic resonance imaging, post radiation therapy

References:
[1] Shu Hang Ng, (2002), Posttreatment imaging of the nasopharynx
[2] JCY Lau, (2020), Post-irradiation Changes and Complications of Nasopharyngeal Carcinoma: A Review of Imaging Features
[3] M. ALTUN,, (1995), UNDIFFERENTIATED NASOPHARYNGEAL CANCER (UCNT): CURRENT DIAGNOSTIC AND THERAPEUTIC ASPECTS

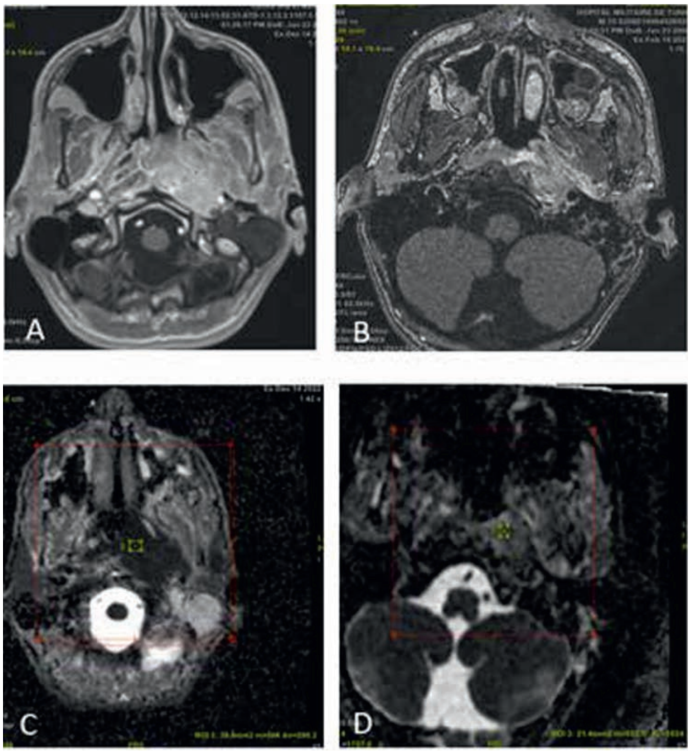

Axial enhanced T1 weighted-imaged with fat saturation before (A) and after radiochemotherapy (B) showing partial regression of the posterolateral aspect of the nasopharynx. Note the decrease of contrast enhancement. Increase of ADC value measuring 0.6x10-3 mm/s before treatment (C) and 1x10-3 mm/s after treatment (D).

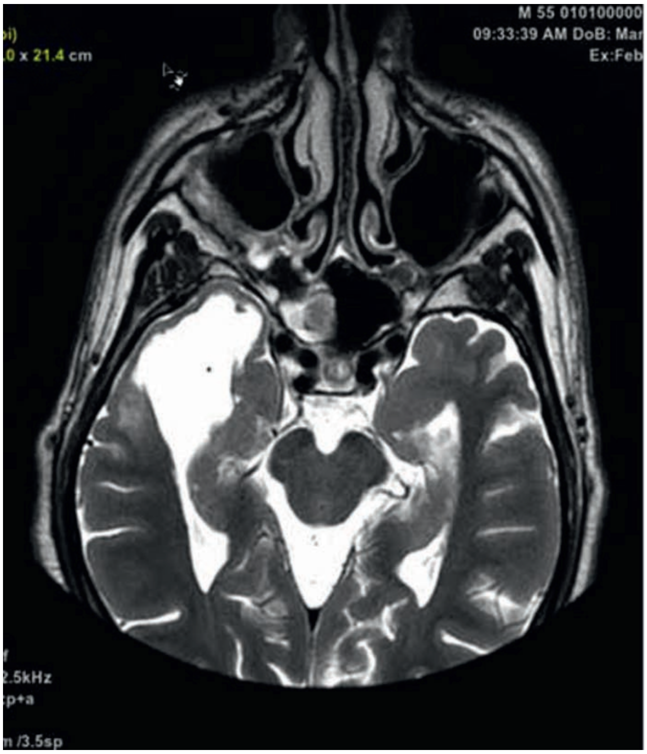

Cerebral axial T2-weighted image in patient followed up for nasopharyngeal carcinoma treated with radiochemotherapy: a subcortical right temporal cavity in communication with the ventricular system related to macrocytic encephalomalacia after right temporal radionecrosis.

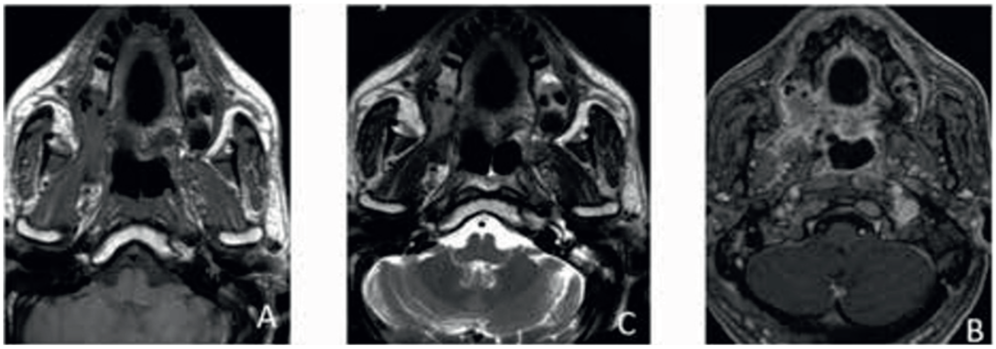

Axial T1-weighted (A), T2-weighted (B) and contrast enhanced T1-weighted (C) images with fat saturation images demonstrating abnormal and heterogenous signal with cortical disruption and enhancement of the right maxillary bone with extension to the medial lamina of the pterygoid process. The features are compatible with osteoradionecrosis.

### Preoperative arterial imaging for vascularized free fibula transplantation for mandibular reconstruction

*Authors: A. Forjaco, J. Lopes Dias; Centro Hospitalar Universitário Lisboa Central, Radiology, Lisbon, Portugal*

**Learning Objectives:**
To review the fibular osteocutaneous free flap surgical procedure and to illustrate the importance of preoperative pelvic and lower limb imaging to prevent complications and improve the chances of a successful surgical outcome.

**Background:** The fibular osteocutaneous free flap is a well-accepted method of mandibular reconstruction for head and neck cancer involving the mandible. The fibular flap includes the fibular bone and the peroneal artery that parallels the length of the bone.

The most feared donor-site complication in fibula flap harvest is foot ischemia secondary to sacrifice of the peroneal artery. Preoperative pelvic and lower limb arterial imaging is extremelly important to detect conditions in which the peroneal artery becomes the main vessel supplying the foot, such as significant arteriosclerotic disease or substantial anatomic variants.

**Imaging Findings and Procedure Details:** Evaluation with Doppler sonography, conventional angiography, CT angiography, or MR angiography is performed to completely delineate the lower limb arterial anatomy, and to detect anomalies such as significant arterial stenosis, with sensitivities in the range of 77-100 % and specificities of 87.6-99.7 %.

In the most common anatomic situation, the popliteal artery bifurcates to become the tibioperoneal trunk and the anterior tibial artery, and the tibioperoneal trunk then bifurcates and gives rise to the posterior tibial artery and the peroneal artery, which terminates just above the ankle, dividing into an anterior perforating branch and a posterior communicating branch, contributing to the vascularization of the dorsum of the foot and to the plantar foot arch.

Normal variants occur in 5-7 % of the population, and some of them may require modification of the surgical approach taken.

Contraindications to free fibula flap transfer include significant atherosclerotic disease in the abdominal aorta, iliac or femoral arteries, or popliteal artery, prior major trauma to the limb, and some specific congenital variants.

**Conclusion:** It is essential to perform a safe and comprehensive clinical and imaging evaluation of the lower limb arterial supply before surgery, to improve the chances of a successful outcome at the recipient mandibular site and to prevent complications such as foot ischemia.

**Keywords:** fibula transplantation; mandibular reconstruction; lower limb arterial anatomy

### Radiological assessment of Cranio vertebral junction abnormalities: A simplified review

*Author: M. S. Swarup; Vardhman Mahavir Medical College and Safdarjung Hospital, Radiology, New Delhi, India*

**Learning Objectives:**
1. Review of embryologic development of bones comprising the complex cranio vertebral junction (CVJ)
2. Depiction of various anatomical landmarks of CVJ region on imaging (CT and MRI)
3. Illustration of various craniometry methods (Lines and Angles) used in radiological assessment and diagnosis of CVJ anomalies
4. Multimodality image based review of various CVJ abnormalities along with salient radiological findings highlighting the importance of craniometry measurements

**Background:** Cranio vertebral junction includes posterior skull base (occiput and clivus), first two cervical vertebrae and supporting ligaments. Knowledge of CVJ anatomy and abnormalities is important due to presence of vital soft tissue structures of cervico medullary junction i.e. medulla oblongata and spinal cord. CVJ abnormalities may be congenital, developmental or secondary to any acquired disease process. Cross sectional imaging with CT and MRI plays essential role in delineation of complex anatomy and pathologies. MRI is often the modality of choice as both bony and soft tissues abnormalities are exceedingly well visualized. Multiplanar CT is better in delineating minute bony abnormalities.

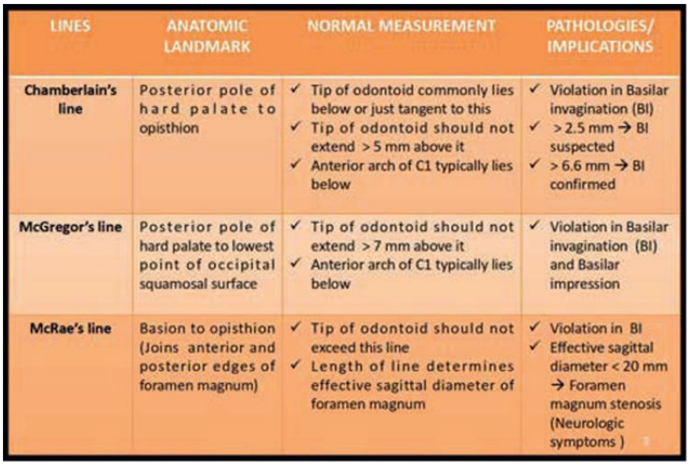

| LINES | ANATOMIC LANDMARK | NORMAL MEASUREMENT | PATHOLOGIES/ IMPLICATIONS |
|---|---|---|---|
| Chamberlain's line | Posterior pole of hard palate to opisthion | ✓ Tip of odontoid commonly lies below or just tangent to this<br>✓ Tip of odontoid should not extend > 5 mm above it<br>✓ Anterior arch of C1 typically lies below | ✓ Violation in Basilar invagination (BI)<br>✓ > 2.5 mm → BI suspected<br>✓ > 6.6 mm → BI confirmed |
| McGregor's line | Posterior pole of hard palate to lowest point of occipital squamosal surface | ✓ Tip of odontoid should not extend > 7 mm above it<br>✓ Anterior arch of C1 typically lies below | ✓ Violation in Basilar invagination (BI) and Basilar impression |
| McRae's line | Basion to opisthion (Joins anterior and posterior edges of foramen magnum) | ✓ Tip of odontoid should not exceed this line<br>✓ Length of line determines effective sagittal diameter of foramen magnum | ✓ Violation in BI<br>✓ Effective sagittal diameter < 20 mm → Foramen magnum stenosis (Neurologic symptoms) |

Craniometry lines

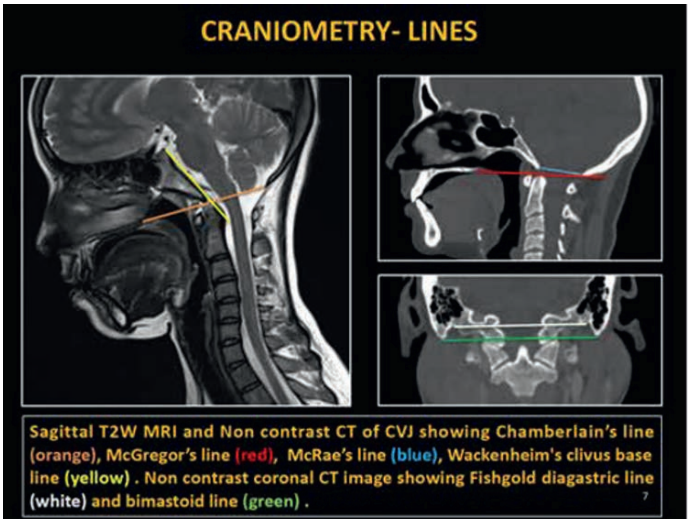

Craniometry lines

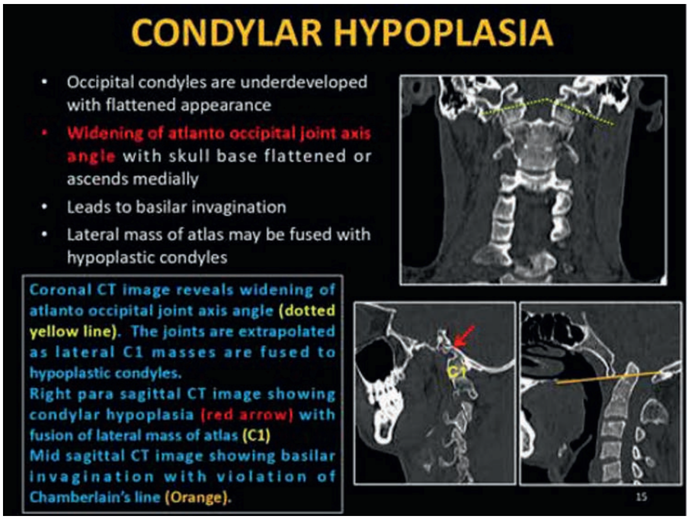

Occipital condylar hypoplasia

**Imaging Findings and Procedure Details:** The following entities will be presented as a case based review with highlighting typical imaging findings and appropiate craniometry measurements.

*I. Congenital CVJ anomalies and malformations*

*A. Occipital Anomalies:*

Condylus tertius

Condylar hypoplasia

Basiocciput hypoplasia

Atlanto-occipital assimilation

*B. Atlas anomalies:*

Posterior arch anomalies

Anterior arch anomalies

*C. Axis anomalies:*

Os odontoideum

Odontoid hypoplasia and aplasia

Ossiculum terminale

Segmentation failure C2-C3

*D. Consequent conditions:*

Platybasia

Basilar invagination

Atlanto axial instability / Subluxation

*E. CVJ anomalies associated with syndromic conditions:*

Klippel Feil syndrome

Chiari malformation

Mucopolysaccharidoses

*II. Acquired abnormalities of CVJ*

A. Trauma

B. Infection – Tuberculosis

C, Inflammatory – Rheumatoid arthritis

D. Neoplasms involving the CVJ - Metastasis and primary bony neoplasms (chordoma)

**Conclusion:** Knowledge of various Craniometry lines and angles is essential for unravelling the complex pathologic anatomy of CVJ. Identification of few anatomic landmarks and basic knowledge of development of CVJ structures is essential for evaluation of CVJ abnormalities.

**Keywords:** Cranio vertebral junction, Os odontoideum, Klippel Feil syndrome, Atlanto-occipital assimilation

### Role of Magnetic Resonance Imaging in Diplopia

*Authors: E. J. Lee[1], N. H. Kim[2]; [1]Dongguk University Ilsan Hospital, Radiology, Goyang-si, Gyeonggi-do, Republic of Korea (South Korea), [2]Dongguk University Ilsan Hospital, Neurology, Goyang-si, Gyeonggi-do, Republic of Korea (South Korea)*

**Learning Objectives:**

1. To understand the anatomy of the neural pathway governing eye movement
2. To describe MR imaging findings of lesions that affect neural pathway causing diplopia
3. To help narrow the differential diagnosis in a case of diplopia.

**Background:** A wide variety of pathologic entities can cause diplopia. Some of the diplopia may be caused by life-threatening conditions such as stroke, infection, and tumor which require careful assessment and accurate diagnosis. In patients with diplopia, a careful assessment of cranial nerves III, IV, VI, paramedian pontine reticular formation (PPRF), and medial longitudinal fasciculus (MLF) is needed. Also, familiarity with anatomy and the pathways of the cranial nerves III, IV, and VI at either the level of the brainstem, subarachnoid space, cavernous sinus, superior orbital fissure, and orbit is important to exclude life-threatening critical abnormality.

**Imaging Findings and Procedure Details:**

1. General overview of diplopia
2. Normal anatomy for the neural pathway of eye movement
3. A variety of pathologic conditions causing diplopia
   1) Brain and brain stem nuclei
      - Stroke
      - Tumor
      - Multiple sclerosis
      - Inflammation
      - Vascular malformation
   2) Subarachnoid space
      - Congenital agenesis or hypoplasia of 3rd, 4th , or 6th nerves
      - Aneurysm
      - Hemorrhage
      - Meningeal disease (meningitis, carcinomatous, lymphomatous)
      - Granulomatous inflammation (sarcoidosis, Wegener)
      - Trauma
   3) Cavernous sinus
      - inflammation
      - Infection
      - Carotid artery aneurysm
      - Tumor
      - Cavernous carotid arterial venous fistula
   4) Superior orbital fissure
      - Tumor
      - Inflammation
   5) Orbit
      - Tumor
      - Cellulitis
      - Thyroid-associated ophthalmopathy

**Conclusion:** A good understanding of the normal anatomy and function of the neural pathway governing eye movements will help in the clinical evaluation of diplopia. Familiarity with the clinical and imaging features of lesions affecting neural pathways in patients with diplopia is important for the early detection of lesions and narrowing of the differential diagnosis.

### Scalp lesions – A bit of a headache?

*Authors: J. Flexen, A. Alade, A. Timmis, X. Kowa, S. Morley, T. Beale; University College Hospitals London, London, United Kingdom*

**Learning Objectives:**
- Anatomy of the scalp
- A practical approach to imaging scalp lesions
- Differentials for scalp lesions including diagnoses to consider when the calvarium is involved
- Understanding the suitability of image-guided biopsies

**Background:** Scalp swellings are common in the general population.[1] Diagnosis of these lesions can pose a challenge because of their varied aetiologies.[1] The radiologist plays a central role in assisting clinicians in differentiating 'do not touch' entities (for example benign lesions and even normal anatomical structures) from lesions which require urgent sampling for histological and/or microbiological analysis.[1][2] A number of scalp lesions have well recognised imaging characteristics, for example epidermal inclusion cysts, subgaleal lipomas and calvarial haemangiomas. In other entities, the appearances can however be more indeterminate.[3] The aim of this poster is to provide an overview of the layered anatomy of the scalp, tips to aid anatomical localisation of lesions and key multimodality imaging features that suggest an aggressive underlying pathology.

**Imaging Findings and Procedure Details:** The superior spatial resolution of ultrasound lends itself to the detailed layered anatomy of the scalp. We will focus on the sonographic anatomy of the scalp and its identifiable layers correlating this to cross-sectional imaging.

We will exhibit ultrasound, computed tomography and magnetic resonance imaging examples of a range of scalp pathologies. We will share a range of scalp pathologies with an emphasis on the more unexpected diagnoses (calvarial granulomatosis, rare soft tissue and bone tumours, haematological malignancies) and benign mimics of aggressive entities. All cases have histopathological and/or microbiological confirmation.

**Conclusion:** The poster will be relevant to clinicians and radiologists with an interest in head and neck pathologies.

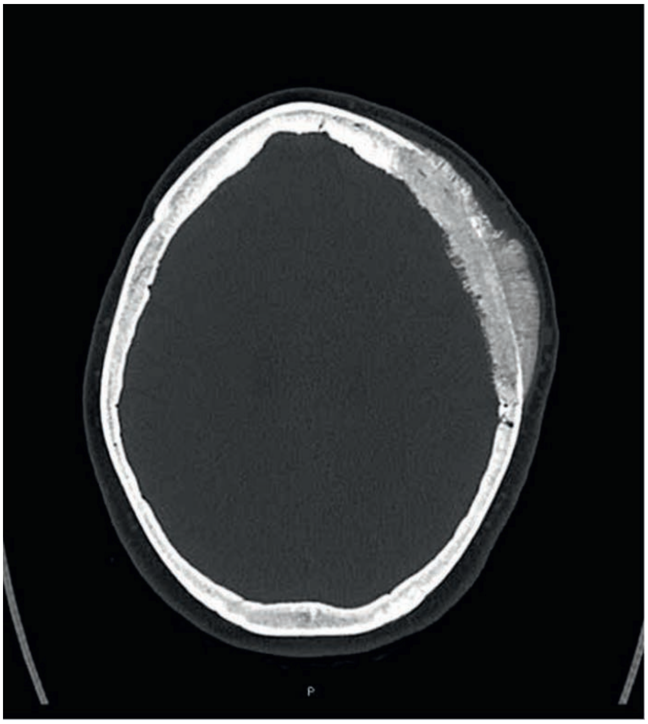

Axial CT image showing a full-thickness abnormality of the left frontoparietal convexity of the skull. The bone displays an aggressive periosteal reaction and there are accompanying intracranial and extracranial extraosseous soft tissue components. The extracranial soft tissue component was biopsied and diagnosed as an intraosseous meningioma.

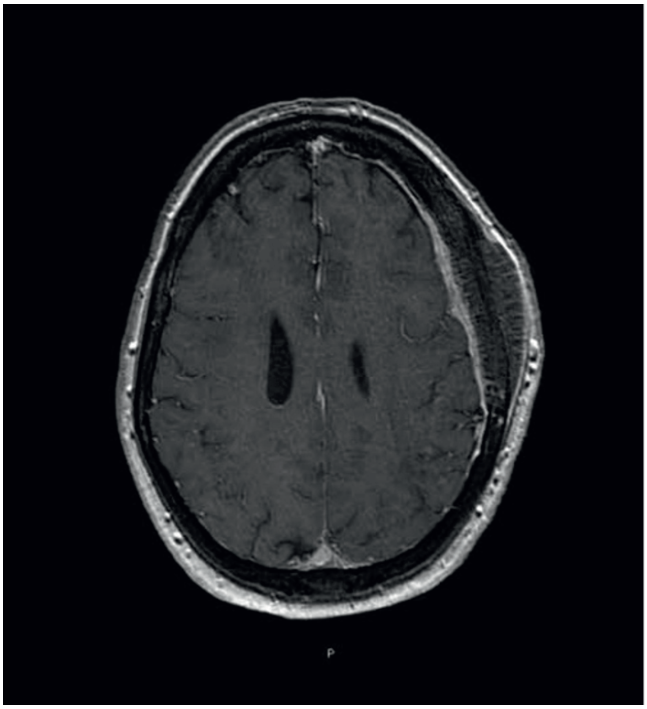

Contrast enhanced T1 weighted axial image from the same patient shows the enhancing dural component of the intraosseous meningioma.

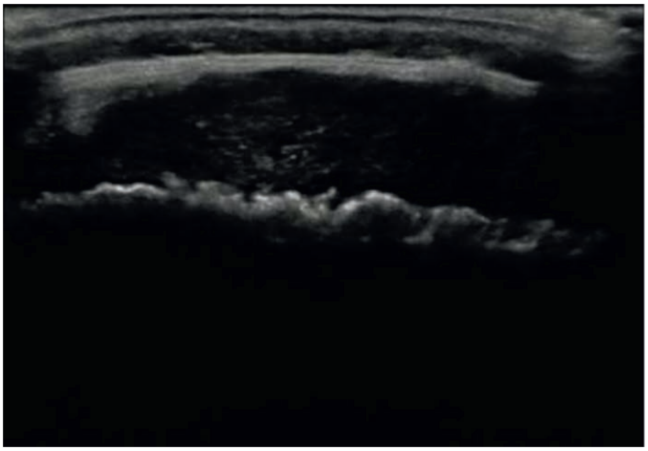

Ultrasound image from the same patient shows the extracranial soft tissue component of the lesion which was targeted with ultrasound guided core biopsy.

**Keywords:** Scalp, Calvarium, Ultrasound

References:
[1] Kawaguchi M, Kato H, Matsuo M, (2019), CT and MRI features of scalp lesions, Radiol Med, https://pubmed.ncbi.nlm.nih.gov/31270724/, 2023-03-31
[2] Carratalá RM, Cabezuelo MEC, Herrera I, Azabarte PC, Tapias SD, de la Presa RM, Tejada FXA, (2017), Nontraumatic Lesions of the Scalp: Practical Approach to Imaging Diagnosis: Neurologic/Head and Neck Imaging, Radiographics, https://pubs.rsna.org/doi/epdf/10.1148/rg.2017160112, 2023-03-31
[3] Gomez CK, Schiffman SR, Bhatt, AA, (2018), Radiological review of skull lesions , Springer, Insights into Imaging, https://insightsimaging.springeropen.com/articles/10.1007/s13244-018-0643-0, 2023-03-31

### Secondary Otalgia – More than the five T's

*Author: R. Carter; Oxford University Hospitals Foundation Trust, Radiology, Oxford, United Kingdom*

**Learning Objectives:**

1. Understand the aetiology of secondary otalgia and the nerves involved in this referred pain pathway.
2. Apply this anatomical knowledge to understand the variety and location of pathologies that should be sought for when reviewing head and neck cross-sectional imaging of patients presenting with referred otalgia.

**Background:** Secondary or referred otalgia is thought to occur due to the overlap of sensory innervation of the head and neck and the varied sensory innervation of the ear by cranial nerves V, VII, IX, and X and the upper cervical nerves C2 and C3. Although the exact mechanism cannot be proven, it is assumed that the brain is unable to localise the exact source of the pathology due to these nerves converging onto a single shared neural pathway centrally. Although the underlying aetiology of most patients who present with referred otalgia is usually benign, it can also be the heralding and only symptom of occult head and neck malignancy. CT or MRI is therefore performed in patients where no abnormality is seen on physical examination in the ENT clinic.

**Imaging Findings and Procedure Details:** Benign and malignant pathologies ranging from dental caries and biomechanical pathology of the cervical spine and temporomandibular joints to pharyngeal, sinonasal, oral cavity, salivary gland and thyroid pathologies are represented in this pictorial review to remind the radiologist to perform a systematic review for the potential sources of referred pain on CT and MRI.

**Conclusion:** The radiologist should review specific areas in the head and neck when imaging patients with secondary otalgia by understanding the overlap of innervation of the ear and other head and neck spaces by mulitiple cranial and cerivcal nerves.

**Keywords:** Referred otalgia, cranial nerves, cervical nerves, head & neck malignancy

### Solitary Fibrous Tumors (SFT) in the Head and Neck: A pictorial review

*Authors: V. Pires[1], A. Borges[2]; [1]CHUC, Radiology, Coimbra, Portugal, [2]IPO, Radiology, Lisboa, Portugal*

**Learning Objectives:**

- To review solitary fibrous tumors occurring in different Head and Neck locations.
- To illustrate the main imaging features of SFT in different imaging modalities.
- To highlight the importance of imaging in depicting aggressive features and guiding patient's management.

**Background:** SFTs are rare mesenchymal spindle cell neoplasms most commonly of pleural origin but occurring in many extrathoracic sites. 25% of extrathoracic tumors originate in the head and neck, in order of decreasing frequency from the oral cavity, sinonasal and neck regions. They have no gender predilection and the mean age of presentation is 51 years. The most frequent presenting symptoms of SFTs in the head and neck region is an indolent, painless palpable mass. SFTs are most often benign but may show locally invasive features with bone, vascular and perineural invasion. Malignant SFTs tend to to have a higher growth rate and are featured histologically by the presence of cellular atypia, nuclear pleomorphism, higher mitotic rate and focal areas of necrosis and hemorrhage.

As histopathologic features can be quite varied, immunohistochemistry is mandatory to establish the diagnosis (98% stain positive for CD34 and vimentin and 95% show nuclear expression of STAT6).

Complete surgical resection is the key feature determining the outcome and is a predictive factor for local recurrence. When negative margins cannot be achieved, adjuvant radiotherapy is indicated.

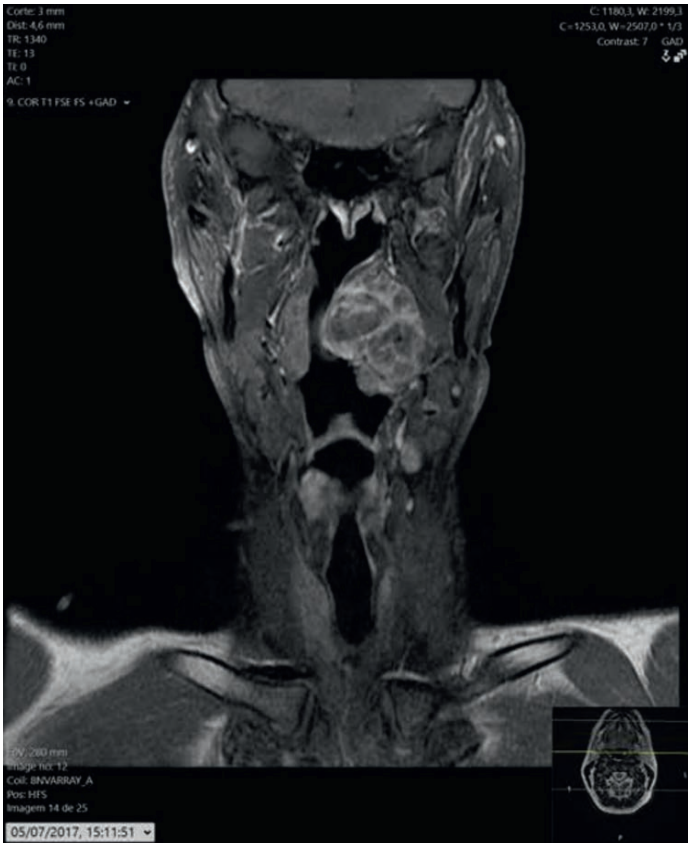

SFT of the Parapharyngeal Space

**Imaging Findings and Procedure Details:** Radiographically, whether by magnetic resonance imaging (MRI), computerized tomography (CT), or ultrasound (US), findings are non-specific. Usually, this diagnosis should be considered in cases of well-defined, heterogeneous hypervascular tumors in the head and neck region. The presence of areas of low signal intensity on T2 weighted MR images that strongly enhance after gadolinium injection are suggestive of this diagnosis and correspond to the presence of dense collagenous components. Tumors do not restrict on DWI and, on perfusion studies, demonstrate a short time to peak and a slow washout pattern time–intensity curve. Presence of locally invasive features such as bone and vascular invasion and perineural spread such raise the suspicion of malignancy as well as hypermetabolism on FDG-PET-CT.

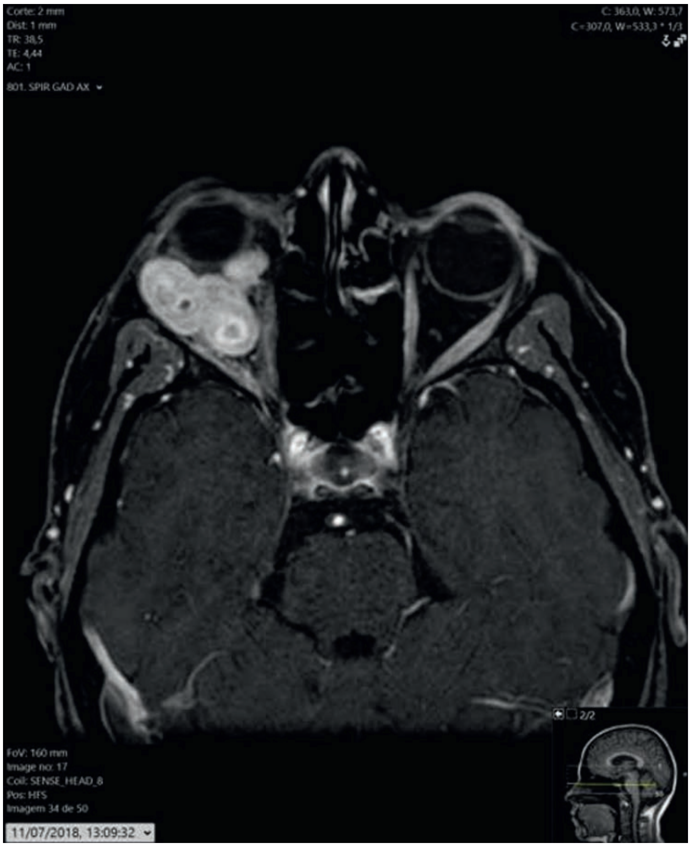

SFT of the orbit

**Conclusion:** Imaging features, although non-specific may be of use in the differential diagnosis of other commoner head and tumors and radiologists are in the best position to raise this possibility. Assessment of local spread and depiction of local invasiveness are key for surgical planning and impact patient's prognosis. Signs of radiological and histological aggressiveness require adjuvant treatment and a close follow-up.

**Keywords:** Solitary fibrous tumor; Head and Neck; Soft tissue tumor;

### Sonographic characteristics of parathyroid lesions: Pearls and pitfalls

*Authors: C. Cefai[1], A. Samuel[1], A. Betts[2], R. Schembri Higgans[2], R. Grech[1], E. Vassallo[1]; [1]Mater Dei Hospital, Medical Imaging Department, Msida, Malta, [2]Mater Dei Hospital, Pathology Department, Msida, Malta*

**Learning Objectives:**

1. Illustrate the sonographic features of histologically confirmed parathyroid (PT) lesions
2. Correlate findings with 99mTc-Sestamibi SPECT and four-dimensional CT (4DCT)
3. Outline pearls and pitfalls of parathyroid ultrasound (US)

**Background:** The PT gland is an endocrine organ responsible for calcium metabolism, situated in the visceral space adjacent to the posterior surface of the thyroid gland. There are two paired glands. The superior pair originate from the fourth pharyngeal pouch and descend with the thyroid gland, reaching its posterior third. The inferior parathyroids arise from the third pharyngeal pouch, descending with the thymus to the inferior pole of the thyroid, although they may reach the anterior mediastinum. Normal parathyroid glands are usually not detectable on US. In standard practise, US and $^{99m}$Tc-Sestamibi SPECT are complementary first-line imaging tools (Morris et al., 2022). US is efficient, well-tolerated, non-invasive, cost-effective and accessible. Nonetheless, it remains operator-dependent and unable to diagnose intrathoracic ectopic parathyroid lesions. It may sometimes be challenging to distinguish PT lesions from other pathological entities such as lymph nodes or ectopic thyroid tissue with ultrasound alone. When a lesion remains equivocal on US, particularly in the context of a negative $^{99m}$Tc-Sestamibi SPECT, or in cases of suspected ectopic PT lesions, integration with a 4DCT is indicated.

**Imaging Findings and Procedure Details:** A total of 517 neck ultrasounds performed locally between January 2017 and March 2023 were reviewed by two radiologists. Data was retrieved from PACS and anonymised. The sonographic characteristics were documented; 244 scans were reported to have suspicious PT findings; from the latter a total of 89 patients had histologically confirmed PT lesions, 81% of which were confirmed PT adenomas and 9% represented PT hyperplasia. Results were correlated with the available $^{99m}$Tc-Sestamibi SPECT and 4DCT scans. 2% of patients demonstrated overlap with other pathologies on US.

**Conclusion:** US has a pivotal role in diagnosing PT lesions in patients with clinically confirmed hyperparathyroidism but a negative $^{99m}$Tc-Sestamibi SPECT study. Good US technique is key to ensure that low-lying PT lesions are not missed. 4DCT is useful when PT lesions cannot be reliably distinguished from thyroid nodules or lymph nodes at ultrasound. Cystic PT lesions must not be confused with cystic metastases from thyroid papillary or squamous cell carcinoma and a thorough sonographic assessment of the neck is warranted to exclude any pertinent ancillary findings.

**Keywords:** Parathyroid, Ultrasound, 99mTc-Sestamibi, 4DCT, histopathology, lymph node

### Sutures, small canals, foramen and linear structures of the temporal bone – Differential of normal anatomy and pathology

*Authors: K. Döring[1], A. Giesemann[2]; [1]Hannover Medical School, Diagnostic and Interventional Neuroradiology, Hannover, Germany, [2]Hannover Medical School, Interventional and Diagnostic Neuroradiology, Hannover, Germany*

**Learning Objectives:** Excerpt of various pathologies and norm variants
**Background:** The temporal bone combines a wide variety of details in a very small space, making it one of the most complex osseous structures. Numerous anatomical variants are known. According to Goethe's maxim: "One sees only what one already knows and understands" (Goethe 1819), the knowledge and understanding of this is essential and decisive for a purposeful and good interpretation of the present imaging.
**Imaging Findings and Procedure Details:**
*Norm variants*
The petrotympanic fissure is named after Johann Heinrich Glaser (1629-1675) and therefore also bears the eponym Glaserian fissure. The petrotympanic fissure is a small fissure in the temporal bone that connects the mandibular fossa (glenoid) and the tympanic cavity and contains the chorda tympani as a nerve guidance structure. Cochlear clefts are normally seen in children. It is a normal variant without any relationship with hearing loss and should not be confused for otosclerosis, which commonly affects the area anterior to the fissula ante fenestram or the pericochlear otic capsule. If the norm variant of a stapedia artery is present, arising from a persistent meningeal artery, the spinosal foramen is either absent or very small. Normally, the spinosal foramen is located in the posteromedial part of the ala major ossis sphenoidalis posterolateral to the foramen ovale and contains the meningeal artery, the meningeal vein, and the spinosal nerve.
*Various pathologies*
The average width of the vestibular aqueduct (VA) at its midpoint measures 1-2mm. In some cases, the VA is not visible. Often in these cases there is a coincidence with Meniere's disease. On the other hand, if the VA is dilated beyond the norm, it is referred to as large vestibular aqueduct syndrome - the most common malformation of the inner ear. Normally, there is an IAC. In malformed inner ears a double or divided canal can be detected. In doubled cases of the IAC the facial nerve can take a route following the trigeminal nerve and turning sideways at the entrance of the Gasserian ganglion towards its own geniculate ganglion. Additional venous chanals can be encountered in the normal temporal bone, but are more often found in association with malformed inner ears, especially frequent in complete aplasia of the semicircular canals. This malformation is a major criteria for CHARGE syndrome. A persistant petrosquamal sinus (PSS) is usually accompanied by an additional foramen; consecutively, the jugular foramen is often hypoplastic.
**Conclusion:** Being aware of the numerous anatomical details and deviating norm variations of the temporal bone helps to recognize pathologies unerringly, to name them as such as pathology and to classify them in the clinical context and the present clinical symptomatology.

**Keywords:** temporal bone, pathologies, norm variants, DVT

### Syphilis: Heads (and Necks) Up for the Great Mimic

*Authors: L. Ellis[1], A. Clayton[1], O. Seddon[2]; [1]Cardiff and Vale University Health Board, Clinical Radiology, Cardiff, United Kingdom, [2]Cardiff and Vale University Health Board, Public Health Wales, Cardiff, United Kingdom*

**Learning Objectives:** After describing a case, to review the imaging findings that should highlight Syphilis as a differential diagnosis to malignancy in the context of head and neck imaging. Additionally, the risk factors and different stages of the disease will be discussed.
**Background:** Syphilis is an infection caused by the gram-negative spirochete Treponema pallidum[1]. Globally the disease is most prevalent in the WHO African region, however in recent years the prevalence in the UK and Europe has continued to increase[2], with new diagnoses more than doubling in the UK between 2013 and 2018[3]. Undetected, syphilis can persist for years. The initial presentation can therefore be when the disease is in its tertiary stage with the formation of gummata, including in the oral cavity[1]. This can result in syphilis mimicking other head and neck pathology such as malignancy[4].
**Imaging Findings and Procedure Details:** This poster describes a patient referred for a left retromolar trigone lesion which clinically was suspicious for a tumour. MRI demonstrated abnormal signal and contrast enhancement in the left retromolar trigone and buccal region. Reactive cervical lymph nodes were seen on ultrasound. Pathology from biopsy of the lesion was negative for malignant cells and the patient was found to have active syphilis on serology. The lesion responded to antibiotic therapy, and a diagnosis of tertiary syphilis was therefore made.
**Conclusion:** In conclusion, Syphilis can present late and therefore a patient's initial presentation can mimic malignancy in head and neck imaging. With rates of syphilis in the UK and Europe increasing, a Head and Neck Radiologist should be aware of the disease as a differential for oral lesions in patients, particularly in those with risk factors. This will potentially aid earlier diagnosis and management.

**Keywords:** Syphilis, Gumma

References:
[1] National Institute of Clinical Excellence, (2019), Syphilis: What is it?, NICE, https://cks.nice.org.uk/topics/syphilis/background-information/definition/, 2023-03-19
[2] National Institute of Clinical Excellence, (2019), Syphilis: How common is it?, NICE, https://cks.nice.org.uk/topics/syphilis/background-information/prevalence/, 2023-03-25
[3] Public Health England , (2019), Addressing the increase in syphilis in England: PHE Action, PHE, https://assets.publishing.service.gov.uk/government/uploads/system/uploads/attachment_data/file/806076/Addressing_the_increase_in_syphilis_in_England_Action_Plan_June_2019.pdf, 2023-03-21
[4] Ripoll E, Montironi C, Alós L, Pujol T, Berenguer J, Oleaga L., (2017), Oropharyngeal Syphilis: Imaging and Pathologic Findings in Two Patients., Head Neck Pathology, 399-403, 11(3), doi: 10.1007/s12105-016-0758-x. Epub 2016 Oct 3. PMID: 27699642; PMCID: PMC5550383., 2023-03-12
[5] Gaillard F, Rasuli B, Smith D, et al. , (2020), Syphilis. Reference article,, Radiopaedia.org, https://radiopaedia.org/articles/54679, 2023-03-21

### Technical highlights and challenges for a successful harvest – A guide to sentinel node mapping in head and neck melanoma

*Authors: E. Kristóf[1], N. R. Kelemen[1], P. Prekopp[2], T. Györke[1], H. Sükösd[3]; [1]Semmelweis University, Medical Imaging Centre, Department of Nuclear Medicine, Budapest, Hungary, [2]Semmelweis University, Department of Otorhinolaryngology and Head and Neck Surgery, Budapest, Hungary, [3]Semmelweis University, Medical Imaging Centre, Department of Radiology, Budapest, Hungary*

**Learning Objectives:** Sentinel lymph node biopsy (SLNB) is indicated in several types of malignancies, including melanoma, in case of no evidence for macroscopic metastasis. Managing the clinically node negative neck in head and neck melanoma patients is challenging due to the complex anatomy and the small lymph node size. Therefore, there is high demand for precise lymphoscintigraphy and for exact morphological information. We present our experiences on a group of patients with head and neck melanoma and share details about the impact of dynamic imaging and SPECT/CT on the surgical success rate.
**Background:** Despite the fact that melanoma is the least common type among malignant skin tumours, it is responsible for 80% of the mortality of all skin malignancies. Correct staging of the primary tumour is crucial, the depth of invasion and special risk factors give us the probability for nodal or distant metastases. SLNB, as part of staging, can determine the presence of microscopic metastasis in the first draining lymph nodes. In case of head and neck melanoma, the special challenge for the surgeon is that these nodes are often superficial, and sometimes along or inside the parotid gland.
**Imaging Findings and Procedure Details:** Since 2021, 40 head and neck melanoma patients came for radiocolloid injection the day before or on the day of surgery. Right after the intracutaneous injection, dynamic images are recorded to find every first node in all possible lymph drainage routes. After visualisation of possible sentinel nodes, SPECT/CT imaging is done, to depict the exact anatomical locations of the radioactive nodes. Several factors make the correct identification harder, for example: too close injection site, possible nonvisualisation of sentinel nodes, distinguishing lymphatic vessels from true nodes, periparotid or intraparotid nodes. CT reconstruction, volume rendering and good patient positioning can improve the diagnostic value. In some cases late images are also needed. We would like to share our experience about these challenges and their possible solutions, which can contribute to a better understanding and a good clinical practice.
**Conclusion:** Finding the right sentinel nodes in head and neck melanoma patients is a continuous challenge for the surgeon, but also for the nuclear medicine physician. It is crucial to know the potential lymphatic drainage of the region, to look for superficial nodes as the possible spreading route of melanoma and to give precise guidance for the surgeon as well as for follow-up.

**Keywords:** Melanoma, sentinel node, lymphoscintigraphy, hybrid imaging, SLNB

### The Assessment of the Oral Cavity on Ultrasound Examination

*Authors: J. Gao, B. Jackson, E. Pilavachi, S. Wilkinson, T. Beale; University College London Hospitals, Royal National ENT and Eastman Dental Hospitals, London, United Kingdom*

**Learning Objectives:** Review the ultrasound technique and clinical use of oral cavity assessment.
**Background:** Oral cavity pathology can be evaluated on ultrasound examination using both external and intra-oral techniques. Ultrasound examination provides high resolution imaging, that is not degraded by dental artefact, unlike traditional imaging. The technique of ultrasound assessment also allows a truly clinical approach, as the examination requires taking a short history, direct visual inspection and can provide a target for FNAC or TRU cut biopsy.
**Imaging Findings and Procedure Details:** External and Intra-oral approaches using curvilinear, linear and ‘hockey stick’ probes are used in clinical assessment. We review the technical approach of ultrasound of the oral cavity. Ultrasound provides a useful adjunct to the interpretation of cross-sectional imaging of the oral cavity or, for smaller lesions, can be used alone. We compare ultrasound to MRI, where relevant, to demonstrate the clinical use of ultrasound.
**Conclusion:** We demonstrate the clinical role of ultrasound examination of the anatomical subsites of the oral cavity with cases comparing MRI images of the same pathology.

**Keywords:** ultrasound, oral cavity, technique

### The Clinical Role of Ultrasound in Assessing the Superficial Structures of the Head and Neck

*Authors: S. Wilkinson, J. Gao, B. Jackson, E. Pilavachi, T. Beale; University College London Hospitals, Royal National ENT and Eastman Dental Hospitals, London, United Kingdom*

**Learning Objectives:** Pictorial review of the clinical role of ultrasound in assessing pathology of the nose, nasolabial, lips, cheek, and ear. We review normal ultrasound anatomy, imaging techniques and the sonographic features of pathology with respect to cross-sectional imaging findings.
**Background:** Ultrasound, due to its high resolution, is ideally suited to assessing pathology in the superficial structures of the head and neck. We demonstrate the clinical role of ultrasound in assessing superficial head and neck structures including the nose and nasolabial region, lips, cheek, and ear with normal ultrasound anatomy and pathology of these regions.
**Imaging Findings and Procedure Details:** External ultrasound images are acquired using linear and ‘hockey stick’ probes. Normal anatomical and pathological sonographic images are presented. Where relevant, these are compared to cross-sectional imaging with pathological ultrasound findings vs. MRI and / or CT.
**Conclusion:** We demonstrate the clinical use of ultrasound in assessing the structures and pathology of the superficial structures of the head and neck region, highlighting the clinical use and advantages of ultrasound.

**Keywords:** ultrasound, head and neck

### The exam technique and clinical role of ultrasound in assessing the cranial and peripheral nerves and their exiting neural foramina of the head and neck

*Authors: S. Wilkinson, E. Pilavachi, J. Gao, B. Jackson, J. Flexen, T. Beale; University College London Hospitals, Royal National ENT and Eastman Dental Hospitals, London, United Kingdom*

**Learning Objectives:** To demonstrate, by pictorial review, the technique in assessing the course of the exiting and forming nerves in the head and neck region by ultrasound assessment.
**Background:** Ultrasound is often underutilised in the assessment of the neural structures of the head and neck region. The superficial course of the nerves, in conjunction with the high resolution of ultrasound, allows clear visualisation and assessment. However, due to the complexity of anatomy in this region and the clinical use of ultrasound in the outpatient setting, hesitancy ensues due to the complexity of structures and time limitations.
**Imaging Findings and Procedure Details:** Ultrasound assessment of the facial, vagus, accessory, hypoglossal, brachial plexus, and phrenic nerves within the head and neck regions. We review examination techniques and normal anatomy. Comparison to cross-sectional imaging, where relevant, to demonstrate the clinical use of ultrasound in identifying neural structures and pathology.
**Conclusion:** Imaging via ultrasound is an underutilised tool in assessing the neural structures of the head and neck region.

**Keywords:** ultrasound, head and neck, neural structures

### The eye of the storm: The many faces of orbital metastases

*Author: A. Borges; Instituto Português de Oncologia de LIisboa, Radiology, Lisbon, Portugal*

**Learning Objectives:**
- To review the epidemiology of metastatic orbital disease
- To demonstrate the features of orbital metastases on different imaging modalities and on different orbital compartments
- To provide flowcharts for diagnostic workup according to different clinical settings

**Background:** Orbital metastases comprise 1-3% of all orbital tumors, affect 2-5% of oncologic patients, is the first site of metastatic disease in 30% and the first sign of neoplastic disease in 2-15% of patients (orbital metastasis from an unknown primary). They result from hematogenous spread, can be single, multiple and bilateral and the most common primaries are breast, lung, kidney and prostate cancers and melanoma. The anterior orbital compartment is more frequently affected. They are divided into ocular and extra-ocular: ocular metastases comprise 80% of ocular tumors whereas extra-ocular metastases are responsible for 2-8% of orbital tumors.
**Imaging Findings and Procedure Details:** Orbital metastases can present in 3 different clinical settings: patients with a known metastatic cancer, patients with a non-metastatic primary cancer or in patients without a known primary tumor. Imaging findings, whether on MRI, CT or US are non-specific. Differential diagnosis with primary orbital tumors and other orbital diseases can be challenging and require a high degree of suspicion. Whereas primary orbital tumors tend to be unilateral, intraconal and affect extra-ocular muscles, metastatic disease is more commonly extraconal, with or without involvement of the bony orbital walls, tends to affect the superolateral quadrant and can be bilateral. Imaging patterns include a diffuse infiltrating lesion, a discrete extraconal mass with or without bone destruction or a focal intraconal mass. A less common pattern results from a desmoplastic reaction, coursing with enophthalmos and palpebral retraction seen in scirrhous breast cancers. Lesions tend to be ill-defined with locally aggressive features and show restricted diffusion on DWI.
Ocular metastases affect primarily the uvea, the most vascularized ocular layer and are often accompanied by a subretinal effusion. Ultrasound has the best diagnostic accuracy but US images are of limited use for radiotherapy planning. Therefore, MR imaging, ideally with a surface coil, is preferred. Imaging features useful in the differential diagnosis with primary tumors and other ocular diseases include the presence of multiple/bilateral lesions, a broad choroidal base and hypervascularity with a peripheral pattern of contrast enhancement.
**Conclusion:** Orbital and ocular metastases can be a diagnostic challenge. Imaging is crucial in the differential diagnosis and for treatment planning. Different diagnostic workup flowcharts should be used for different clinical settings.

**Keywords:** Ocular metastases, orbital metastases, orbital lesions, metastatic disease

### The missing condyle: Not a simple open and shut case

*Authors:* *K. A. Eley[1], S. R. Watt-Smith[2], M. Cameron[3]; [1]University of Cambridge, Radiology, Cambridge, United Kingdom, [2]Eastman Dental Institute, London, United Kingdom, [3]Cambridge University Hospitals NHS Foundation Trust, Oral & Maxillofacial Surgery, Cambridge, United Kingdom*

**Learning Objectives:**
1. To describe the congenital and acquired pathologies which can result in an absent mandibular condyle.
2. To revise the relevant anatomy and embryology to guide consideration of appropriate differential diagnoses of an absent condyle.
3. To provide an overview of the important imaging findings which guide the management options and their rationale.

**Background:** The absent mandibular condyle is a rare phenomenon with a wide differential. We present the case of a 24-year-old female who presented with a 3-year history of left sided jaw pain and clicking. The main impetus for seeking referral came because a friend in veterinary practice noted significant asymmetry of the condylar regions on an impromptu ultrasound.
**Imaging Findings and Procedure Details:** Initial imaging with orthopantomogram demonstrated an entirely absent left mandibular condyle, which was confirmed on CT and MRI. The right condyle was unaffected and functioned normally. Cross-sectional imaging demonstrated soft tissue at the expected location of the condylar head and neck, slightly larger than the contour of the expected but absent bone. The condylar fossa and articular eminence were normally formed but demonstrated small areas of bone erosion. There was a non-united fracture of the coronoid process; an unusual finding which suggested possible generalised demineralisation. The muscles of mastication were symmetrical. The imaging findings remained stable over a 2-month follow up period.
Open biopsy was performed removing all the replaced soft tissue. Histology demonstrated fibrous collagenous tissue with no evidence of inflammation or malignancy. Samples of bone demonstrated remodelling changes only.
The clinical case is used to highlight the anatomy and embryology of the mandibular condyle, to aid in formulating a differential diagnosis.
The reader will be asked to consider the various pathologies including tumour (e.g. a giant cell tumour that had burned out), hormonal destruction, and trauma. The extent of condylar resorption, which was not joint centric, makes joint pathologies less likely.
An orthopantomogram 3 months following biopsy demonstrated no significant interval change in the post-operative appearances.
The diagnostic dilemma poses additional management difficulties. We review the potential options for managing the absent condyle and the important imaging findings used to guide these decisions.
**Conclusion:** The rare presentation of an absent condyle within a young female patient with an indolent course and absence of inflammation or malignancy on histology resulted in a diagnostic and management dilemma, for which a definitive histological diagnosis was not identified.

**Keywords:** Mandible; condyle; resorption; tumour.

### Tracing the Tract to the Tongue: Causes of a Hypoglossal Nerve Palsy

*Authors:* *L. Ellis[1], C. Greenall[2]; [1]Cardiff and Vale University Health Board, Clinical Radiology, Cardiff, United Kingdom, [2]Cwm Taf Morgannwg University Health Board, Radiology Department, Vale of Glamorgan, United Kingdom*

**Learning Objectives:**
To revise the anatomy of the twelfth cranial nerve and the imaging features of both acute and chronic Hypoglossal Nerve Palsy (HNP). Secondly, to review the skull base and extracranial causes for HNP specifically, as these are most likely to come under the remit of an ENT/maxillofacial radiologist. Finally, to discuss the significance of HNP in the staging of nasopharyngeal cancer.
**Background:** HNP is a rare condition that causes restricted tongue movement. Although dysphagia is a relatively common presenting complaint to ENT, dysphagia secondary to HNP is uncommon. The hypoglossal nerve passes through, and adjacent to, multiple structures at both the skull base and extracranially[1]. The nerve can therefore be affected by pathology at any of these structures during its course. Because of this, following the anatomy of the hypoglossal nerve is a useful method to identify the cause of a HNP when clincial imaging appearances of the tongue[2] are consistent with a palsy.
**Imaging Findings and Procedure Details:** This educational poster describes a patient who was referred for imaging for dysphagia and asymmetry of the oropharynx. This was initially believed to be due to a nasopharyngeal malignancy which had directly invaded the oropharynx. However, on MRI the appearance of the tongue was consistent with a HNP, and the CT demonstrated direct tumour invasion of the hypoglossal canal rather than the oropharynx. The identification of a unilateral HNP was significant as it resulted in upstaging of the tumour[3].
**Conclusion:** In conclusion, this case highlights the importance of a radiologist being able to detect HNP on head and neck imaging. The aetiologies of a HNP are numerous[4] and therefore an ENT/Maxillofacial radiologist should have an awareness of these and the anatomy of the hypoglossal nerve to best aid diagnosis and management.
References:

**Keywords:** Hypoglossal Nerve, Palsy

### Ultrasound Anatomy and Examination Technique of the Oral Cavity and Oropharynx

*Authors:* *B. Jackson, J. Gao, S. Wilkinson, E. Pilavachi, T. Beale; University College London Hospitals, Royal National ENT and Eastman Dental Hospitals, London, United Kingdom*

**Learning Objectives:** Review the ultrasound anatomy and technique of the oral cavity and oropharynx.
**Background:** Ultrasound is a useful tool to evaluate the oral cavity and oropharynx, for both clinical assessment of pathology as well as understanding the anatomy of this region. The underutilisation of ultrasound in the oral cavity and oropharynx may be due to lack of experience and understanding of the normal anatomy of these regions.
**Imaging Findings and Procedure Details:** We review the normal ultrasound anatomy of the oral cavity and oropharynx. Probe positioning and examination technique with curvilinear, linear and 'hockey stick' probes are demonstrated.
**Conclusion:** We demonstrate the normal anatomy and examination techniques that help adequately visualise these regions.

**Keywords:** ultrasound, oral cavity, technique, head and neck

### Usual and unusual lesions of cerebellopontine angle region – An image-based review with segmental approach

*Authors: M. S. Swarup[1], S. Sahoo[2], D. Tripathy[2]; [1]Vardhman Mahavir Medical College and Safdarjung Hospital, Radiology, New Delhi, India, [2]SCB Medical College and Hospital, Radiology, Cuttack, India*

**Learning Objectives:**

1. To provide an image-based review of various common and uncommon lesions of the cerebellopontine angle (CPA) region.
2. To provide a segmental imaging approach for evaluating CPA region mass lesions
3. To highlight characteristic imaging findings

**Background:** Masses in the cerebellopontine angle region are readily identified on cross-sectional images. Vestibular schwannomas and meningiomas constitute the great majority of these tumors. Besides this, a number of unusual lesions are also encountered in this region. Differential diagnosis of masses in this region can be simplified by using a segmental imaging approach i.e detection of the epicenter or the site of origin. Morphologic characteristics on cross-sectional images are also important in making a preoperative diagnosis (attenuation at computed tomography, signal intensity at magnetic resonance imaging, enhancement pattern, shape and margins, extent, mass effect, and adjacent bone reaction).

**Imaging Findings and Procedure Details:**
An image-based discussion of the following pathologies will be provided. Magnetic Resonance Imaging (MRI) is the modality of choice for the evaluation of these lesions with Computed Tomography (CT) useful for the delineation of lesions of the bony skull base.
A. Cranial nerve origin:
Vestibular schwannoma (classic appearance and variants)
Trigeminal schwannoma
B. Cerebellopontine angle cistern origin:
Epidermoid cyst
Melanocytic tumor/ melanoma
Lipoma
C. Meningeal origin:
Meningioma
Arachnoid cyst
D. Vascular origin:
Aneurysm/vascular ectasia of posterior fossa
E. Skull base origin:
Chondroid tumor (Chondrosarcoma)
Chordoma
Leukemic deposits/metastasis
Aneurysmal bone cyst
Paraganglioma (Glomus Jugulare)
F. Lesions originating from the cerebellum or fourth ventricle and extending to the CPA region:
Glioma
Ependymoma
Medulloblastoma

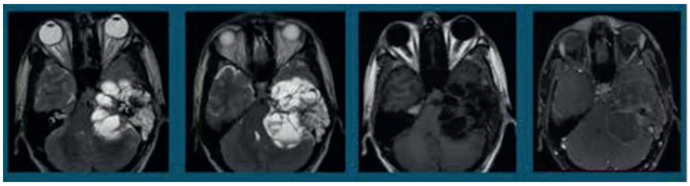

Aneurysmal bone cyst of petrous temporal bone

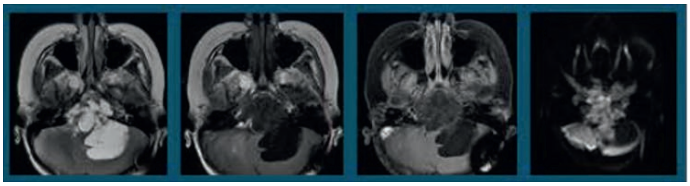

Chordoma of clivus and petrous temporal bone

**Conclusion:** The knowledge of the patient's age, the exact origin and location of the lesion, and characteristic imaging findings can help the radiologists in narrowing the differential diagnoses.

**Keywords:** cerebellopontine angle, cistern, schwannoma, magnetic resonance imaging
